# Qualitative Insights Into Patients' and Family Members’ Experiences of In-Hospital Medication Management After a Critical Care Episode

**DOI:** 10.1016/j.chstcc.2024.100072

**Published:** 2024-06

**Authors:** Richard S. Bourne, Mark Jeffries, Eleanor Meakin, Ross Norville, Darren M. Ashcroft

**Affiliations:** a Critical Care Department, Sheffield Teaching Hospitals NHS Foundation Trust, Sheffield, England; b Department of Pharmacy, Sheffield Teaching Hospitals NHS Foundation Trust, Sheffield, England; c Division of Pharmacy & Optometry, School of Health Sciences, Faculty of Biology, Medicine and Health, University of Manchester, Manchester, England; d National Institute for Health and Care Research Greater Manchester Patient Safety Research Collaboration, School of Health Sciences, Faculty of Biology, Medicine and Health, University of Manchester, Manchester, England; e Patient and Public Representative, England

**Keywords:** critical care, engagement, family, medication, patients, qualitative research

## Abstract

**Background:**

Patient recovery after a critical illness can be protracted, requiring a care continuum that extends along a patient pathway from the critical care unit, hospital ward, and into the community care setting. High-quality care on patient transfer from critical care, including medication safety, is facilitated by education for patients and families, family engagement, support systems, and health care professional (HCP)-patient communication. Currently, uncertainty exists regarding how HCPs can and should engage with critical care patients and family members about their medication.

**Research Question:**

What are the views and experiences of critical care patients and family members about their involvement in, communication about, understanding of, and decision-making related to their medication after transfer from critical care to the hospital ward?

**Study Design and Methods:**

This qualitative study used semistructured interviews, conducted with critical care patients and family members after transfer from critical care to a hospital ward in a large National Health Service hospital trust. Anonymized transcripts of interviews were analyzed thematically using a coding framework developed from understandings of patient and family engagement in medication administration.

**Results:**

Twenty-seven participants (15 patients and 12 family members of patients) completed the interviews. We identified five themes and 15 subthemes, providing an overview of patients’ and family members’ views on medication management during acute illness and ongoing recovery. Themes identified were: impact of acute illness and treatment burden on preexisting illness, preexisting knowledge and capability, beliefs about persons roles and expectations, care continuity and individualized information exchange, and engagement in practice.

**Interpretation:**

This study demonstrated that critical care patients and family members want to engage with HCPs about medication administration. HCPs must take an individualized approach to communication and timing, acknowledging the dynamic interplay between patients and family members, using multimodal forms of communication.

**Take-home Points**

**Study Question:** What are the views and experiences of patients and family members about medication management after a critical care episode?

**Results:** Five themes were identified, providing an overview of patients’ and family members’ views on medication management engagement during acute illness and then ongoing recovery.

**Interpretation:** This study demonstrated that patients and family members want to be engaged about medication management plans, requiring health care professionals to take a tailored approach, appreciating the dynamics in patient and family member roles.

The care of critically ill patients increasingly looks beyond patient survival, seeking to understand and then address challenges to optimal patient recovery and quality of life. Awareness is developing within the critical care community of the need for a care continuum that extends along a patient pathway from the critical care unit, to hospital ward, and into the community care setting.^1^

The challenges presented to critical care survivors are multifactorial and complex, including physiologic and psychological morbidity requiring integrated health and social care interventions.^1^ Medication-related problems also must be considered. A critical care episode often entails a dramatic change in a patient’s medication regimen. On admission, most chronic medicines are stopped as clinically inappropriate or unimportant,^2^ with many acute medicines commenced to treat the presenting clinical condition, to keep the patient comfortable, or both. On recovery, the patient may be exposed to problematic polypharmacy^3^: failure to restart important chronic medicines,^4^ continuation of inappropriate acute medication,^5^^,^^6^ and medication transfer errors.^7^ Problematic polypharmacy contributes to adverse drug events on the hospital ward,^8^ often persisting into the community setting,^9^ and predisposes patients to early hospital readmissions.^10^^,^^11^

Several interventions have sought to improve medication safety for critical care survivors transferring to a hospital ward and on hospital discharge.^7^ To date, none of these medication-related interventions have included patient and family engagement.^7^ Patients and family members are the only consistent element in care continuity, supporting safety in transitions in care,^12^ and care transition interventions focused on patients and family reduce the risk of hospital readmission.^13^ High-quality care on patient discharge from intensive care, including medication safety, may be facilitated by education for patients and families, family engagement and support systems, and health care professional (HCP)-patient communication.^14^^,^^15^ The value of direct discussion and information provision, including medication, have been reported previously in critical care patients undergoing transitions in care.^16^^,^^17^ In contrast, HCPs perceive barriers to routine engagement and information provision for patients and families after a critical care episode.^14^^,^^18^ HCPs’ and public representatives’ beliefs can differ on the importance of medication information provision to improve medication safety,^15^ contributing to patient reports of inadequate communication, predisposing them to anxiety or distress on transition of care.^19^

We aimed to explore the views and experiences of critical care patients and family members about their involvement, communication, understanding, and decision-making related to medication administration after transfer from critical care to the hospital ward to help inform the development of interventions to improve the care process.

## Study Design and Methods

### Setting

A single-center large hospital trust in the north of England comprising three general critical care units over two sites. All medical and surgical specialities were provided within a major trauma center. Critical care services and multiprofessional staffing resources met National Health Service (NHS) service specifications.^20^

### Study Design and Approvals

This qualitative study used semistructured interviews with patients and family members of patients who had been admitted to critical care and subsequently were transferred to a hospital ward. Interview topic guides were developed from the Continuum of Patient Engagement^21^^,^^22^ and the experiences of older people after discharge.^23^ Our research team’s patient and public advisors reviewed and contributed to the final study protocol and topic guide (e-Appendix 1). NHS ethical (London-Westminster Research Ethics Committee Identifier: 22/LO/0582) and NHS Health Research Authority (Identifier: IRAS 317565) approvals were granted for the study.

### Sampling, Eligibility, and Recruitment

Sampling for interviews was purposive and stratified to include patients (and the family members of patients) who required different levels of critical care (defined as level 2 [eg, basic organ system monitoring and support for ≥ 2 organ systems] or level 3 [eg, advanced respiratory monitoring and support alone, or advanced level monitoring and support for ≥ 2 organ systems]).^24^ Patients and family members were eligible to participate in the study after 3 days acclimatization to the hospital ward environment to minimize any additional care transition anxiety.^17^ Exclusion criteria were patients (and family members of patients) younger than 18 years, being nonfluent in English, or receiving critical care for < 24 h or undergoing a subsequent hospital ward stay of > 7 days. The 7-day exclusion was adopted to capture contemporary participant memories of the critical care and ward transfer experiences. Potential patient and family member participants were screened by critical care-trained research nurses (led by E. M.). Patients were excluded if they experienced delirium, were unable to speak (eg, tracheostomy in situ), were deteriorating clinically (increasing National Early Warning Score),^25^ or were receiving end-of-life care. A family member was a person with a close familial, social, or emotional relationship to the patient and was not restricted solely to next of kin. Family members had to confirm they were aware of the patient’s medication before or during the acute hospital admission and had to have visited their relative at least once during the patient’s stay in critical care. Eligible participants were provided with written information about the study and were given 24 h to confirm written consent to participation.

Single interviews were conducted by telephone by one author (M. J.) who is an experienced (male) social scientist researcher in medication safety and was unknown to the participants. Patients were interviewed in the hospital and family members were interviewed at home. Interviews were audio digitally recorded and transcribed verbatim without field notes. Transcripts were not returned to participants for checking. Data collection was conducted iteratively alongside analysis. Interviews continued until a range of level 2 or level 3 patients (and family members of patients) had been interviewed and data saturation was achieved. We considered data saturation achieved when further interpretation of codes and themes would not provide further insights.^26^

### Data Analysis

Analysis was thematic, providing a rich, detailed account of the different perspectives of the research participants.^27^ Thematic analysis involves familiarization with the data, the generation of codes, the interpretation and combining of codes into themes, and the reviewing of those themes. Because we developed a coding framework from understandings of patient and family engagement^21^^,^^23^ and inductively from reading and rereading early transcripts, we also followed a template approach.^28^ As such, the framework was refined and developed iteratively and inductively as coding proceeded (e-Appendix 2). M. J. led on the analysis using NVivo software version 12 (QSR International). Independent coding of a representative sample of eight participant transcripts by R. S. B. supported ratification and consolidation of the coding framework. R. S. B. and M. J. met weekly to discuss the data, to reach coding framework consensus, to interpret themes, and to confirm data saturation.^26^ Codes and coded extracts then were tabulated, grouped, and interpreted into potential themes. After discussion and further interpretation across the research team (R. S. B., M. J., E. M., and R. N.), the final themes and subthemes were agreed on (e-Appendix 2). The study is reported according to the Consolidated Criteria for Reporting Qualitative Studies.^29^

## Results

Forty-seven potential participants were provided study information, 30 consented to participation, and 27 were interviewed (Table 1).^24^ Nonparticipation reasons were clinical deterioration (one patient) and unreachable for interview within the study timescale (one patient and one family member). Interviews were conducted between December 2022 and August 2023. Mean interview duration was 21 min (range, 9-31 min) for patients and 28 min (range 17-41 min) for family members. We identified five themes and 15 subthemes, providing an overview of patients’ and family members’ views on engagement with the management of medication administration during the acute illness and ongoing recovery in hospital (Table 2).

**Table 1:** Summary Characteristics of Patient and Family Member Participants

Participant Type	Level of Critical Care^24^	Admission Type	Clinical Specialty (Primary)
Patients (n = 15)	Level 2 (n = 8) Level 3 (n = 7)	Elective (n = 8)Emergency (n = 7)	Colorectal surgery (n = 4); cardiology medicine (n = 2); hepatobiliary surgery (n = 2); plastic surgery (n = 2); upper GI surgery (n = 2); general surgery (n = 1); hepatology medicine (n = 1); vascular surgery (n = 1)
Family members of patients (n = 12)	Level 2 (n = 6) Level 3 (n = 6)	Elective (n = 4) Emergency (n = 8)	Respiratory medicine (n = 4); colorectal surgery (n = 2); cardiac surgery (n = 1); cardiology medicine (n = 1); hepatobiliary surgery (n = 1); thoracic surgery (n = 1); upper gastrointestinal surgery (n = 1); vascular surgery (n = 1)

**Table 2:** Summary of All Themes and Subthemes

Themes	Subthemes
Impact of acute illness and treatment burden on preexisting illness	Changing ability of patient to assimilate or recall information provided, or both
	Treatment burden: emotional, psychological, and physical
Preexisting knowledge and capability	Confidence in their knowledge of medicines and health care
	Impact on expectations for and level of patient and family engagement
Beliefs about persons roles and expectations	Expectations for and level of patient and family engagement
	Dynamics in patient and family roles and levels of responsibility
	High degree of trust in health care system and professionals
	Health care professionals’ willingness to engage and share medication information or decisions
Care continuity and individualized information exchange	Differences in care and resources between critical care and ward environments
	Availability of, quality of, and satisfaction with information exchange
	Timing, format, and health care professional delivery of information exchange
Engagement in practice	Information exchange between patient and health care professionals
	Acquired knowledge and understanding from engagement
	Decision-making about medicines and medication plan
	Impact of level of engagement on patient, family, or both

### Impact of Acute Illness and Treatment Burden on Preexisting Illness

Patients commonly reported having limited ability to assimilate or recall information because of sedation, delirium, sleep deprivation, or short-term memory problems (Table 3). Other barriers to effective communication included patients with a tracheostomy requiring adaptive communication methods.

“And even though I couldn’t communicate either because I had a trachea in that prevented me from speaking . . . the staff they knew that I was getting frustrated because people couldn’t lip read particularly well, so they got me a board so I could write on it and communicate that way.” (patient 24_L3)

**Table 3:** Impact of Acute Illness and Treatment Burden on Preexisting Illness

Subtheme	Additional Supporting Quotations
Changing ability of patient to assimilate or recall information provided, or both	“Well, I remember being very dazed when I first left and quite confused and I kept forgetting quite a lot of things and I gradually got better and my brain came round again.” (patient 06_L2)
	“In the end I hadn’t had enough sleep to make a sensible decision so I just . . . in the end I said well, if the consultant thinks that, let’s go home, you know.” (patient 12_L2)
	“So she’s literally, she has a 24-hour window, so she can remember what’s gone off in the past, and she knows who everybody is, and she knows of things coming up in the future, she doesn’t know anything we talked about yesterday. . . . She’ll have forgotten that.” (family 21_L3)
	“Because his hearing, he’s not wearing his hearing aids at the moment. . . . Yeah, at the moment. He doesn’t quite understand what they’re telling him.” (family 17_L2)
	“And then on his transfer to the ward, he was totally with it, you know, he was back with us. So I asked him yesterday . . . about his medication and everything, and he knew everything, all the medication that he was getting, from the nurses.” (family 23_L2)
Treatment burden: emotional, psychological, and physical	“And that [not being able to have pain relief because of contraindications] makes things more difficult for patients like myself. Because we’re often sat in pain, and there’s nothing, really, we can do about it. And it’s very frustrating, and it makes the quality of life more unbearable.” (patient 27_L3)
	“And not having the understanding of what they are being administered and why they’re being administered it, you . . . well, for me personally, I felt almost like I was a bit blind to it all, like I didn’t fully understand what he was going through, you know, it made me feel quite . . . emotional is probably the best word because you’re already in a very scary situation.” (family 25_L3)

These acute clinical factors sometimes prevented patient involvement in decisions, often relying on HCPs and family members to inform their care. On acute illness recovery, a patient’s baseline capacity to be involved in decisions often returned. Experiences of acute pain and other sequelae changed their perspectives on the importance of long-term medication within the context of the current treatment burden.

“You can’t focus . . . you can’t see a clear path through. And even discussing it with you, now, you can’t see a clear problem.” (patient 28_L3)

Patients experienced an emotional and psychological burden with decisions, and family members experienced a burden of care with emotional, psychological, and physical consequences for them, requiring management to prevent exhaustion, acknowledging that the patient’s recovery could be protracted. This treatment burden sometimes limited patients’ and family members’ abilities to engage and ask questions about medications.

### Preexisting Knowledge and Capability

Knowledge of medicines seemed to be a prerequisite to patient or family confidence, or both, in managing and discussing the medicines. Lack of knowledge hampered conversations (Table 4).

“I asked about the . . . proton pump inhibitor . . . I thought I recognized it and [patient] wasn’t sure what it was . . . everything else was straightforward really. She seemed to have been given everything she should have been given. She seemed to have been given it at the right time.” (family 16_L2)

**Table 4:** Preexisting Knowledge and Capability

Subtheme	Additional Supporting Quotations
Confidence in their knowledge of medicines and health care	“And once I realized I wasn’t taking it [metformin]. I was a bit stubborn, I stood my ground, like. I said, you’re not doing that until this is sorted. Within 12 hours, it was sorted.” (patient 01_L2)
	“He should’ve been on medicines before he went into hospital. The only one he took regularly was omeprazole for his really bad indigestion. . . . When the paramedics took him to the ambulance on Friday night, I had a scour around the room and I found several boxes, . . . all of them were unopened. That’s correct . . . he was prescribed but not taking them.” (family 20_L2)
	“So I was quite inquisitive because I do work in the NHS myself. . . . I’m not clinical but I do work in a clinical environment, so I do have a bit of an understanding, so it made it a lot easier for me. But I think like my partner’s mum, for instance, who doesn’t have any medical terminology or anything, she found it really hard to understand what all the medicines were and what they were for.” (family 18_L3)
	“I like to know what medicine my dad’s on, and I do read up a lot on it and . . . why he’s taking it, what he’s not taking it . . . because I have a science background . . . so everyone left it on me, you’re our doctor in the house, you sort it.” (family 03_L2)
Impact on expectations for and level of patient and family engagement	“Well, as far as I know, knowing the cholesterol situation won’t change or my potential glaucoma, so I would anticipate carrying on . . . but I assume there’s some sort of clinical review in a month or so, I can put it across to him then.” (family 16_L2)
	“I just accept, that is the way they work . . . . You know, because like I said, we’ve never had no dealings with hospitals.” (family 17_L2)

NHS = National Health Service.

On occasion, patients prompted or led decisions about the medications, adding value and understanding. Specific family members assumed responsibility for aspects of care such as medication based on their prior knowledge and experiences. Patient and family knowledge of any preexisting illnesses and medications had an impact on their expectations of levels of future health care engagement. Those with more limited knowledge had low expectations of engagement. Others realized that they needed to seek further information when able.

“Because I don’t know nothing about it or about the medicines, I don’t want to say anything. Does that make sense, until I know what the medicine is. That’s what I’m planning to do. That’s what me and my wife are planning to do. When I leave the hospital, I’m going to find out what medicines I’m on, what they’re for.” (patient 07_L2)

### Beliefs About People’s Roles and Expectations

Some patients were clear that they should be involved in decisions about medication, whereas others were less expectant or sure of their position (Table 5).

“I don’t need encouragement. That metformin, as soon as I realized it, I were in . . . straightaway. And if I had any doubts whatsoever what I was being given without explanation, I’d have pulled them. I’m not shy in coming forward, not by a long way.” (patient 01_L2)

**Table 5:** Beliefs About a Person’s Roles and Expectations

Subtheme	Additional Supporting Quotations
Expectations for and level of patient and family engagement	“And when it turned out in the end I did want to know. Whereas perhaps I’d thought, oh no, I’d rather not know, thanks, as I said, but as it . . . when it turned out in reality it was a case of, well actually, yes, I do want to know what you’re doing.” (patient 04_L2)
	“Rather than just speak to my dad, they’d speak to all of us. The doctor not so much tried to speak directly to my dad, me and my brother or my partner have to actively involve ourselves in that conversation, ask some questions, which I don’t mind doing.” (family 20_L2)
	“Especially when somebody is into, like, even changing all the medications, and going back to, like, obviously what this is about. . . . You feel like you’re taking them, or distracting them a little bit, which makes me feel a bit guilty.” (family 29_L3)
Dynamics in patient and family roles and levels of responsibility	“My mum’s always been very fiercely independent, she’s a retired teacher and she’s very articulate . . . I think she just finds it a little bit overwhelming now and wants me to be there to listen to possibly ask the questions, you know . . . because initially they’ll ask me to go out . . . well, my mum has to ask, and some of them almost look at me like it’s an inconvenience, why would you possibly want to come back in? I’m thinking, well, actually, it’s not me, my mum’s just said to you, she wants me in. So it’s not me being difficult, my mum says, can my daughter be in?” (family 11_L3)
	“Especially in the first couple of days when he weren’t 100 percent, if by any means I wasn’t there when the doctors came round, he would struggle to give me that information. He would say, oh, they said I might be able to go home in the next couple of days. I’m like oh right, what did they say about your antibiotics, IV, what they going to do about that?” (family 18_L3)
High degree of trust in health care system and professionals	“I suppose you put a lot of trust in people that know what they’re doing, don’t you? So you don’t really feel like you need to ask because you think they know exactly what they’re doing.” (family 15_L2)
	“So, as long a somebody explains something to me and it makes sense in my head, then I’m happy to go with their recommendation because they’re the professionals, I put my trust them, and nobody’s seen me wrong yet.” (patient 24_L3)
Health care professionals’ willingness to engage and share medication information or decisions	“Well, to begin with, I was in an induced coma, so I was on medication . . . so even though I obviously didn’t see anybody, the medical staff that treated me, whenever they did anything they would come to my ear and tell me what was happening, and almost keep me involved in everything that was happening.” (patient 24_L3)
	“Because the doctors come round in the morning and they’ll come and they’ll actually talk to you. And they’ll talk to you and say, well, this is what we want to do. . . . It gives you the confidence because, well, hold on a minute, they know what they’re going to do. They’ve started to bring me into the conversation with themselves and that’s it.” (patient 07_L2)
	“In regards to in-depth information is what I wanted, pretty poorly up until the point of speaking to the doctor . . . on the respiratory ward. I mean the guys in critical care were fairly vague with what kind of medicine they were giving.” (family 20_L2)

Patient views could change over the course of the critical care stay as they wanted greater involvement. Family members had expectations to be involved in discussions and decisions, for example, when they believed that their relative had reduced capacity either in the long term or short term, although some were hesitant, believing that their questions may impact negatively on HCPs’ ability to undertake their core tasks.

Patients with family members tended to have a dynamic relationship in which each participant played the leading role according to the acute or long-term capabilities of the patient to be involved actively in their care at the time. When the patient was critically ill, family member advocacy was strong, then reduced as the patient recovered their capabilities, until the patient led input into their own care again.

“I feel that, if they seek out the family, in order to . . . just make sure that there was certain medications that they were aware they were on beforehand. Because I know, one of the major ones . . . is she’s on citalopram.” (Family 29_L3)

In critical care, patients reported positive experiences of HCPs’ willingness to engage and take the time to explain treatment decisions effectively when the patient had the capacity to do so.

“So, I feel like . . . yeah, it was a bit strange because obviously choices were out of my hand to begin with, but when I was communicative and once I had questions, people had all the time in the world to explain to me what was happening. So, I feel like I’ve had as much communication as I possibly could have.” (patient 24_L3)

Other examples of good practice emerged in which the patient believed that they were engaged and involved in conversations about their care, although some patients wanted more detailed information about the medication they were receiving in critical care. Participants expressed a high degree of trust in HCPs’ capability to make the most informed decisions about a patient’s care.

“And it was having that trust in critical care. I’m very experienced with [name of hospital] and it’s a great hospital, the staff are fantastic. So, we’ve just trusted that they’ve made the right decisions for [name of patient] when she was unable to do so.” (family 20_L2)

### Care Continuity and Individualized Information Exchange

Patients and family members highlighted several differences in the physical environments and care team resources between critical care and the hospital ward, contributing to differences in the intensity and level of care provided (Table 6). However, it was clear that an expectation existed that continuity in care and communication thereof should be maintained. The more limited staffing on the hospital ward negatively impacted the potential for questions and information exchange and continuity of care, including analgesia provision for some. The degree of personalized care and relationships between named HCPs directly caring for the patient and the family members were reduced on the hospital ward.

“Whereas I feel like at the minute I don’t know who’s looking after [patient name], I don’t actually know who the named doctor is or who should be reviewing it. And I think because the nurses are that thinly spread on the ward, I don’t actually really know the nurse.” (family 18_L3)

**Table 6:** Care Continuity and Individualized Information Exchange

Subtheme	Additional Supporting Quotations
Differences in care and resources between critical care and ward environments	“Well, the doctors and nurses have been really brilliant up in the critical unit, and I think once they’re in the ward, it’s totally different. You see more busy, in and out and whatnot and it’s more crowded.” (family 03_L2)
	“He’s on a reducing dose of clonidine . . . but they didn’t have any and they had to order it in from the pharmacy, so that resulted in his second dose being . . . 6 hours late. Whereas in the intensive care, they have their own pharmacy on the ward so obviously that doesn’t happen.” (family 18_L3)
	“And in terms of medication, it’s very much, as I just brought up just a moment ago, it’s at set times, it’s very much . . . yeah, it’s very much like, a lot more structured in the regard of, like, taking account of, there is several more people that the nurses are between. So, with critical care, as you’ll obviously know, you have a nurse per person. But when it’s a general ward, they have to keep that kind of structure, I’ve noticed. Because they are looking after about eight, 10 different patients, so I do understand.” (family 29_L3)
	“I just wish the communication and the quality of everything between the ITU and . . . the ward, that level of care shouldn’t change. I really want that highlighting, it shouldn’t . . . And half the time, you know, you feel as though they don’t even know the person that they’re treating. And that’s the scariest part of it, especially as a relative, to watch that happen.” (family 25_L3)
	“I know that, obviously on the ward, there is a lot of consistently, like, taking people away from their job, and like, that adds to it, you feel like you’re taking people away, even to ask these questions.” (family 29_L3)
	“I’m really sorry . . . that happened to you, and this is why they usually do it that way, this is probably why you felt more pain than we’d like you to have, and we’ll change that from now on and we’ll look at our procedures.” (patient 24_L3)
Availability of, quality of, and satisfaction with information exchange	“Talk to us, just talk.” (patient 22_L3)
	“But if ever I wanted to ask a question, if ever I was concerned about something or I wasn’t sure what was going on, the staff on critical care were amazing and would always take the time to sit down and go through it all with me again . . . it was a bit strange because obviously choices were out of my hand to begin with, but when I was communicative and once I had questions, people had all the time in the world to explain to me what was happening. So, I feel like I’ve had as much communication as I possibly could have.” (patient 24_L3)
	“If you ask them a question, or you’re lucid enough to ask . . . to ask them a question, you want them to be able to answer it without having to stumble over words or without having to use chemistry which you don’t understand. You just want the plain, simple English.” (patient 05_L3)
	“But sometimes when they try to explain to you, it seems like they’re talking to you, like you’re a little child . . . . It upsets you, it gets you angry at first. Then you just, you think to yourself . . . why are they talking to you like this?” (patient 07_L2)
Timing, format, and health care professional delivery of information exchange	“Something more individualized, a summary of what you’re on and why you’re on it and then you can actually look at it and think, oh perhaps I should ask so and so then. That would help to prompt you to ask a question rather than you thinking afterwards, oh perhaps I should have asked so and so. Or when somebody says to you, oh did you ask them so and so, then you think, oh I never thought to ask them that. . . . Whereas if you’ve got it . . . if you’ve got a summary of it in front of you, you can then use it as an aid.” (patient 04_L2)
	“And all this information has been given to him in leaflet form, and, like I say, I shall read that, when he’s back home I shall read it. But I’ve left him to read it because obviously the injury’s happened to him not me. So, you know, it gives him something to look at and read.” (family 23_L2)
	“Yes, and I don’t think I probably asked searching questions. It’s a very strange thing when a relative is so seriously . . . and they’d say to me, is there anything you want to ask, and you’ve almost got . . . and I almost . . . as the weeks went by because she was in there for some time, I would almost say, is there something I should be asking? I guess, they don’t know how much information to give to relatives so I don’t know that I always ask the most pertinent of questions. You know, you’re just kind of like surviving day by day really.” (family 11_L3)
	“We’ve got kids, she’s busy, I’m busy, well, I’m ill. She takes the kids to university, they’re at uni . . . and they’re working.” (patient 07_L2)
	“But the guy was very, very clear. And he spent time with me, he didn’t just come in, bang, bang, bang, bang, bang, bang, make just 10 bullet points and go out. He came and he took his time with me.” (patient 01_L2)
	“She went through the complete list with me . . . very, very carefully . . . some of them they brought out were ours, prior to the hospital visit and some were theirs.” (family 16_L2)

ITU = intensive therapy unit.

Overall, perceptions were positive of the information provision to family members of patients who were critically ill. Patients and family identified the importance of HCPs taking the time necessary to aid communication and to promote a positive patient experience.

“So, I think when they sat us down and explained the process of what would happen to his body and what medications they’d be using and stuff like that, it did make me feel quite reassured that it was a positive thing as opposed to a negative.” (family 18_L3)

As the patients recovered, often an ardent desire to be involved in decisions emerged. Nurses, pharmacists, and other HCPs were said to engage often with patients effectively when discussing their medication, with an overview of medication changes and indications before hospital discharge valued by patients and family members. It was important to patients that information was pitched at the right level for them. Timing information delivery to fit around some family members’ busy work schedules and taking the time to provide the information and explanation in the detail to the individual also was required.

“If he’s going to give it you, new medication or give some, prescribe it or you’re going to get new medication, you should just give a couple of minutes of your time. Just say, look, this is [metformin]. You’re diabetic, you’re this, you’re that . . . you shouldn’t be taking this or . . . It’s just, things like that but you don’t get that anymore. You don’t.” (patient 07_L2)

### Engagement in Practice

Participants reported variability in information exchange around medicines, with several examples of timely, complete, and understandable exchange between patients or family members and HCPs (Table 7). Conversations about medicines were initiated by HCPs or by patients and family members. Some HCPs informed patients and family, but did not engage with them or provided incomplete, inconsistent, or unclear explanations, for example, medication commencing without explanation. This negative engagement could lead to further questions from family members or patients, sometimes leading to disagreement and conflict.

“You never get the full picture with doctors unfortunately. They can be very vague. But you have to ask the right questions otherwise they don’t answer properly, which I have found. And there has to be more than one of you because obviously two people have got different points of view, different questions, different sides of questions to ask. So, it’s easier when there’s a couple of you asking questions.” (family 20_L2)

**Table 7:** Engagement in Practice

Subtheme	Additional Supporting Quotations
Information exchange between patient and health care professionals	“Well basically when they came to give them me I said, you know, what is it you’re giving me? So, they told me what it was and why they were doing it. And I said, well I just want to . . . you know, I’m not being nosy, I just want to know. And they said, no, it’s your body, you’re entitled to know what’s going into it. So, they did tell me what they were giving me, yes. I can’t remember what they were giving me but, yes, they did tell me what they were giving me and why they were giving it to me.” (patient 04_L2)
	“And they were brilliant, they explained everything, how everything had gone. Yeah, I can’t honestly remember all the specific details as to what . . . With regards to what medicines she was on and stuff like that. . . . I mean, we already knew but they took the time to explain it all to us again. Yeah, they were really good.” (family 15_L2)
	“They would explain what they were doing and why they’d done it. But again, like I say, I don’t know if that’s just because I would ask a lot or that’s what they’d usually do, I’m not too sure. From my experience, I do feel like they did explain it a lot to me.” (family 18_L3)
	“I’m not one for shouting unless I really have to but I really blew my top, to be honest with you. It got to a point where we’d asked several times, [patient name] had been so patient. He was, at this point, throwing up, he was in absolute agony. All the progress that he’d made in the ITU had gone. He’d, at one point, asked to switch the machines off and said I don’t want to be here, I can’t do it, he was in that much agony. And as a mother, to hear your son say that, as you can imagine, that was the point that I blew my top and I demanded to see the consultant.” (family 25_L3)
Acquired knowledge and understanding from engagement	“My mum did say to me, . . . ‘I don’t know what medicine they’ve given me this morning,’ . . . so she’s not been told at the moment what medicines she’s having and what it’s doing.” (patient 10_L2)
	“And that was something that I did bring up when she was on critical care, because she was on antidepressants beforehand . . . so she was going to try not taking the antidepressants and the doctor just, nurse just said to her, if you do get low mood speak to us . . . and they’ve already made recommendations to her [general practitioner] should she wish to go back on them.” (family 21_L3)
	“And then there were a couple of other medicines which they never really mentioned the names of. I did ask and they just vaguely said, ‘oh, this is for so and so, this is for so and so.’” (family 20_L2)
	“But it’s enough pain relief that I’m able to sleep and enough pain relief that I can still feel my injury. . . . I think together I feel like I’ve been involved in the decision, we’ve come up with a really good balance.” (patient 24_L3)
Decision-making about medicines and medication plan	“Well, in the end I had to simply accept that, you know, I think someone did mention that there was, and since I’ve realized, somebody somewhere has said that there was a distinct possibility if you go too fast, you get a really . . . with lowering the blood pressure again, you really tank it. . . . And your body is not . . . what are we doing, and that’s the rationale for doing it gradually, I think. So, I accepted that at the end of the day and just went with their advice.” (patient 12_L2)
	“They have been good at, like, asking [patient] for that and letting [patient] make the choices on when he feels ready and things like that, you know, I will give them their dues in that, they have.” (family 25_L3)
	“I think a lot of the decisions haven’t really included us, or like me and my father, sorry, as such, really. It’s more, especially on the critical care unit, it’s like, as I touched on earlier, it was very much said, this is probably what’s going to happen, and these are the medications, and gave us a bit of information about it. But with the nature of the . . . they’re more giving you, like, this is probably what we’re going to give her. Rather than being included, and you’re deciding, or helping us decide.” (family 29_L3)
Impact of level of engagement on patient, family, or both	“When it came to discharge, the consultant was basically saying to me . . . and I’m sure he is a consultant he does know, that you do get postoperative swelling, you know, and it’ll resolve itself within 7, 7 to 10 days but . . . we might want to stay on or we might to think about giving you the diuretics. And then, you know, more like the support nurse thinks . . . this chap is on his own at home, he’s not got a lot of support, he’s not got a family in this area and he’s only got his neighbors and his friends and it’s going to be very difficult for him . . . . And so, you know, I think he would have definitely wanted me to have some sorting out of the edema before I went home.” (patient 12_L2)
	“Yeah. I feel like on reflection the intensive care nurses do an amazing job. They don’t reassure you in the way, they don’t say your loved one is going to be fine, because they are open and honest and they will tell you that they can’t give you that reassurance, despite how desperate you may be for it. They don’t give you any false pretences that your loved one is going to wake up tomorrow and they’re going to be fine . . . . So I think when they sat us down and explained the process of what would happen to his body and what medications they’d be using and stuff like that, it did make me feel quite reassured that it was a positive thing as opposed to a negative.” (family 18_L3)

ITU = intensive therapy unit.

The patient and family members often were unaware of changes made to medications, often reporting having to prompt HCPs when they identified potential problems or uncertainty with medications.

“I’ve not been taking the antidepressants that I take year in, year out. It was only when I challenged them that they discovered they’d not been prescribing them to me.” (patient 28_L3)

Through such prompting of engagement, patients and family members gained knowledge about the medication changes. The extent of this acquired knowledge varied from some or a little knowledge to extensive knowledge.

“I’ve got a complete schedule for everything I have to take and where I have to take it.” (patient 12_L2)

The full range of patient and family involvement in decision-making around medication was encountered. For some, no involvement in decision-making was evident, whereas for others a clear shared decision-making process occurred. The patient and family degree of decision-making seemed to be informed by the previous themes described. Patients and family members appreciated their involvement in decision-making helping them to understand risks and benefits associated with the medication.

“But it’s enough pain relief that I’m able to sleep and enough pain relief that I can still feel my injury. . . . I think together I feel like I’ve been involved in the decision, we’ve come up with a really good balance.” (patient 24_L3)

Patients and family members sought positive and high-level engagement: “Talk to us, just talk” (patient 22_L3). Higher levels of engagement were linked with more positive views about the care being provided and confidence in the health care system.

Engaging with, and provision of, information and explanation was critical to the overall patient and family experience of the care delivered.

“Despite, obviously, the situation that we were in, it does put your mind a lot more at ease when you’re aware of exactly what’s going on, and exactly the medications, and you’re kept informed every day, so, it does genuinely help.” (family 29_L3)

## Discussion

To our knowledge, this is the first qualitative study focused on exploring the views of critical care patients and their family members on their involvement in, communication about, understanding of, and decision-making related to their medications. We identified five themes including 15 subthemes that provide an indication of patients’ and family members’ views on their medications during critical illness and in-hospital recovery. These views were informed by their preexisting knowledge and capabilities around medication and beliefs around their own and HCPs’ roles. Patients and family members wanted to be engaged with decisions regarding medications. Challenges with care continuity were present, and individualized information exchanges the were needed according to the current illness status and recovery progression of the patient. The dynamic relationship with family member’s advocacy role and interplay with a patient’s self-efficacy were important. Engagement in practice demonstrated variability in quality and experience, and these findings provide an opportunity to improve medication-related patient engagement and care.

Patients and family members concurred with HCPs that when acutely ill, patients often were unable to have an active role in decisions about their medications.^18^ During this period, the patient advocacy role of family members was vital. Manias et al^30^ also identified the important role family members have in medication safety in an observational study of communication between intensive care HCPs and patients and family members. They also highlighted the attention required by HCPs to the cues from families in communicating about medications.^30^

Our findings about the importance of information exchange around medication information on transfer from critical care and variability of engagement in practice are consistent with those identified by Cullinane and Plowright,^31^ who identified insufficient information provision for patients and family members before transfer to the hospital ward.^31^ MacTavish et al,^32^ in a small study of critical care patients in a follow-up clinic (n = 47), reported that fewer than one-half of patients were aware that changes had been made to their medication despite most expressing medication concerns and the need for further written information.^32^ Eibergen et al^33^ reported that despite structured counselling about medication changes, only about one-half of the 124 noncritical care patients were able to recall the medication-related information provided.

Elsewhere others also identified the need for multimodal communication of information by patients and family members after a critical care episode. De Grood et al^34^ undertook a multicenter qualitative study of patients’, family members’, and HCPs’ views on transfer from intensive care to the hospital ward. Patients and family members identified the importance of receiving timely and accurate information, including the need for communication aids such as a written information pamphlet. Continuity of communication between HCPs and patient and family could be improved by use of standardized transfer communication tools (eg, written summary of patient course in intensive care and ongoing treatment plan).^34^ The same research team then went on to develop and pilot a patient-orientated discharge summary including medication information after transfer.^35^

Others have reported the importance of an individualized patient engagement approach on critical care to the hospital ward transition.^16^^,^^17^^,^^34^ In a randomized controlled trial (n = 178) of an intervention that provided individualized and structured patient information in verbal and written formats, Cuzco et al^36^ reported successful intervention delivery significantly reduced patient anxiety and depression scores. Medication was one of the components of the information guide provided supporting verbal provision.^36^ The intervention was delivered by research nurses, and implementation in routine clinical practice in critical care would have the known challenges.^37^ Rosgen et al^16^ undertook a qualitative analysis of HCPs’, patients’, and family members’ considerations for a transition in care bundle. They identified the need to increase engagement with patients and family members around existing transition in care tools and the need for direct discussion and follow-up transfer summaries.^16^

The study strengths include a professionally diverse research team including a patient with lived experience of intensive care, adding credibility to our interpretation and reporting of results. Interviews were conducted by a qualitative researcher experienced in medication safety and transitions in care, capturing participant views early in the hospital ward stay. We included patients and family members of both level 2 and 3 patients so that the full range of severity of illnesses were represented. We minimized any potential bias in interpretation of results by dual coding of a subset of interview transcripts, regular reflexive evaluation, and rigorous thematic analysis process. Limitations include exclusion of non-English-speaking patients and family members and that ethical permissions limited recording of participant demographics. This novel study focused on the critical care-to-hospital ward transfer interface, and further research is needed to understand subsequent patient discharge from hospital into community care. The impact of treatment burden on patients and family members may have affected medication information recall adversely, highlighting the importance of multimodal communication as identified by participants. The potential limitation of a single-center study was minimized by representing a large trust with a broad range of clinical specialties and multiple critical care units and sites. Our results may not be generalizable to other health care systems with different funding and delivery models to NHS multiprofessional critical care practice.

Our findings have several implications for clinical practice in patients recovering from a critical illness. First, patients and family members clearly want to be involved in decisions about medication. The degree of involvement will vary by an individual’s (patient and family member) preexisting knowledge and capabilities and the patient’s acute illness severity and recovery trajectory, and hence must be individualized. Patient advocacy by family members is dynamic and must be acknowledged in the direction of information exchanges. Information should be provided in written or electronic forms in addition to verbal exchanges to maximize impact. Medication safety initiatives must engage with patients, family members, and other caregiver stakeholders when codesigning effective critical care transfer and rehabilitation practices. Further research is required to inform how HCPs can deliver these practices routinely and effectively.^38^

## Interpretation

Critical care patients and family members want to engage with HCPs about medication. HCPs need to adopt a tailored approach to communication about patient medication, using multimodal forms and acknowledging the dynamic interplay between patients and family members.

## Funding/Support

This report is independent research supported by the 10.13039/501100000272National Institute for Health and Care Research (NIHR), HEE/NIHR ICA Programme Clinical Lectureship, Dr Richard Bourne [Grant NIHR300444]. M. J. and D. M. A. are funded by the NIHR Greater Manchester Patient Safety Research Collaboration [Grant NIHR204295].

## Financial/Nonfinancial Disclosures

None declared.

## Supplementary Data

e-Online Data

e-Online Data
