# Supplementary material for: Qualitative Insights Into Patients' and Family Members’ Experiences of In-Hospital Medication Management After a Critical Care Episode
Source: CHEST Crit Care. 2024 Jun;2(2):100072. doi: 10.1016/j.chstcc.2024.100072 (PMC11190841; doi:10.1016/j.chstcc.2024.100072)
Supplement: e-Online Data [file mmc1.pdf]

Patient and family views of medication management after a critical care episode

Interview Topic guide

Part A – Patients only

Interviewer introduction:

I am [….] and I am a [critical care pharmacist] [researcher at the University of Manchester. We are carrying out a study looking at medication safety processes when a critical care patient is transferred to a hospital ward. We would like to find out the views of patients who have had a stay in critical care and have been transferred to a hospital ward about their medicines.

This interview will last for about 20 minutes. During this time, I'd like to discuss your experiences of critical care and the transfer to the hospital ward, focusing on your medicines.

- If you agree, I would like to record our interview. This will make sure I have precise details of what we discuss.

- No-one will be able to identify you in any reports containing quotes from your interview.

- Please speak clearly and try to avoid naming locations and staff specifically. Any written report will have any identifiable information (names/locations) removed.

- Any discussions that take place during the study are confidential. If, during the interview, we have concerns about your safety or the safety of others, we may ask you to contact an appropriate person about this, such as your care team, other health professional or a family member. If we consider it to be serious, we may have to report this. In no other circumstances will we discuss with, your family member's hospital doctors, care team or other health professional what we have talked about in the interview or contact them on your behalf.

Unless you have any questions for me, I will start the recording and we can begin

1. Thinking about medicines you were given to take (after leaving critical care). Did you talk to anyone about those medicines (doctor, nurse, pharmacist, other healthcare staff)? Who talked to you? What was that conversation like? Did you have to prompt anyone for details of your continuing medication, or was this discussion initiated by a healthcare staff member?

2. Can you tell me about any general information you were given about those medicines when they were prescribed to you? (Prompts – what were you told about the purpose of the medicine, side effects, how to take it?) How did you feel about the ways that information was given? Do you think you got enough clear and understandable information about your medicines?

3. Thinking about medicines you were given to take (after leaving critical care). Were any of your medicines changed? Who made those changes? Do you know why they were changed? Were you told the reason for these changes and were you encouraged to ask questions? What do you understand about those medicine changes?

4. Thinking about the current plan for your medicines. What do you know about the plan for your medication? Has the plan been explained to you? Have you had a chance to ask questions?

5. Thinking about the decisions made about your medicines. In what ways do you feel you have been included and involved in the decisions about your medicines? (Prompts- In what ways have things been explained to you? In what ways have you had a chance to give your opinion? In what ways have you felt supported or not with the decisions that have been made? Did you feel able, at the time, to be involved in discussions regarding your medication? How do you think patients could be best involved?)

6. How would you have liked staff to communicate with you about your family medicines? Is there a particular way you would like to receive information? (Prompts – for example, a general information sheet or individual patient summary?)

7. Were there any problems with your medicines (either in critical care or on transferring to the hospital ward) you are aware of?

Interviewer conclusion:

Concluding questions

- Is there anything else that you would like to talk about?

- Is there anything that we have talked about you would like to go back to?

Switch off tape recorder

Many thanks for taking the time to help us with this study. Your contribution has been extremely valuable. If you wish we can send you a copy of your interview transcript, and you can also request to receive a summary of the findings of this research study, just ask us. In the meantime please feel free to contact either myself or the other researcher(s) involved if you have questions in future.

Patient and family views of medication management after a critical care episode

Interview Topic guide

Part B – Family members only

Interviewer introduction:

I am [….] and I am a [critical care pharmacist] [researcher at the University of Manchester. We are carrying out a study looking at medication safety processes when a critical care patient is transferred to a hospital ward. We would like to find out the views of the family members of patients who have had a stay in critical care and have been transferred to a hospital ward, about their family member's medicines.

This interview will last for about 20 minutes. During this time, I'd like to discuss your family member's experiences of critical care and the transfer to the hospital ward, focusing on their family member's medicines.

Before we begin, I'd like to provide some further information for the discussion:

- If you agree, I would like to record our interview. This will make sure I have precise details of what we discuss.

- No-one will be able to identify you in any reports containing quotes from your interview.

- Please speak clearly and try to avoid naming locations and staff specifically. Any written report will have any identifiable information (names/locations) removed.

- Any discussions that take place during the study are confidential. However, if you were to tell us something new that could put you or someone else at risk of harm we will ask you to contact an appropriate person about this, such as your care team, other health professional or a family member or, if we consider it to be serious, we may have to report this. In no other circumstances will we discuss with, your family member's hospital doctors, care team or other health professional what we have talked about in the interview or contact them on your behalf.

Unless you have any questions for me, I will start the recording and we can begin.

1. Thinking about medicines your family member was given to take (after leaving critical care). Did you talk to anyone about those medicines (doctor, nurse, pharmacist, other healthcare staff)? Who talked to you? What was that conversation like? Did you have to prompt anyone for details of your family member's continuing medication, or was this discussion initiated by a healthcare staff member?

2. Can you tell me about any general information you were given about those medicines when they were prescribed to your family member? (Prompts – what were you told about the purpose of the medicine, side effects, how they should take it?) How did you feel about the ways those explanations were given? Do you think you got enough information about your family member's medicines? Did you understand what was being said?

3. Thinking about medicines your family member was given to take (after leaving critical care).
   Were any of their medicines changed? Who made those changes? Do you know why they were changed? Were you encouraged to ask questions? What do you understand about those medicine changes?

4. Thinking about the current plan for your family member's medicines. What do you know about the plan for their medicines? Has the plan been explained to you? Have you had a chance to ask questions?

5. Thinking about the decisions made about your family member's medicines. In what ways do you feel you have been included and involved in the decisions about their medicines? (Prompts - In what ways have things been explained to you? In what ways have you had a chance to give your opinion? In what ways have you felt supported or not with the decisions that have been made. How do you think family members could be best involved?)

6. How would you have liked staff to communicate with you about your family member's medicines? Is there a particular way you would like to receive information? (Prompts – for example, a general information sheet or individual patient summary?)

7. Were there any problems with your family member's medicines (either in critical care or on transferring to the hospital ward) you are aware of?

Interviewer conclusion:

Concluding questions

- Is there anything else that you would like to talk about?

- Is there anything that we have talked about you would like to go back to?

Switch off tape recorder

Many thanks for taking the time to help us with this study. Your contribution has been extremely valuable. If you wish we can send you a copy of your interview transcript, and you can also request to receive a summary of the findings of this research study, just ask us. In the meantime please feel free to contact either myself or the other researcher(s) involved if you have questions in future.
