# Supplementary material for: Qualitative Insights Into Patients' and Family Members’ Experiences of In-Hospital Medication Management After a Critical Care Episode
Source: CHEST Crit Care. 2024 Jun;2(2):100072. doi: 10.1016/j.chstcc.2024.100072 (PMC11190841; doi:10.1016/j.chstcc.2024.100072)
Supplement: e-Online Data [file mmc2.pdf]

CONNECTED Patient and Family – Themes, sub-themes with Extracted Quotations

- **Codes developed from Carman et al., 2013; Tomlinson et al., 2020; (May et al., 2014) and inductively from early reading of transcripts.**
- **Codes iteratively developed through coding by MJ and RB**
- **Themes interpreted from groups of codes (from reading and re-reading of transcripts, coding and drawing from Tomlinson 2020)**
- **Themes are labelled with initial label and final label.**
- **Sub-themes are initial sub-themes prior to later merging and refinement**

**Theme 1**	**Initial Theme - Impact of acute illness and treatment burden**<br><br>**Final Theme - Impact of acute illness and treatment burden on pre-existing illness**	
	**Sub Theme**	**Quotations**
	**Ability of patient to absorb or recall information - e.g. levels of sedation, confusion**	R: The thing is I have had an infection and it's been [inaudible 0.02.46] but it is…I have been a bit sore these past two days […] I've had an infection. […] It's been, it [inaudible 0.02.57], it's a bit like, a bit vague to be honest, but I can remember them telling me.<br>I: Right, okay, so because of the infection you've been a little sort of not really [voices overlap 0.03.09].<br>R: Yes, my head has been all fuzzy **[Patient02_L2]**<br><br>Well, the doctors and nurses have been really brilliant up in the critical unit, and I think once they're in the ward, it's totally different. You see more busy, in and out and whatnot and it's more crowded and it's totally different. My dad was getting brilliant care in the critical care unit, but then once he got into the ward, he was getting more confused, [inaudible 16:01] asking what he would like, and then there's another person coming, and so, his dinner got messed up and everything because too many people. My dad does get confused a lot, so it was that we were there we asked for what meal to have or he did say, it's confusing, too many people coming asking me things and everything. Because the critical care unit was one nurse and one domestic person, yeah. So, it was confusing for him in the ward. **[Family03_L2]**<br><br>R: Oh yes, there was always, you know, the downside and normally it would give you…they would give you, this is going to be such and such and if it's a really a bad situation then it's the only thing that we've got so far in this world that's going to help you. There was…well I can't…there must have been at some time a situation like that but I can't remember to being, but there again you're normally at a situation or a point in your life where it really is ridiculously hard to understand because you don't have a clue what you've got anyway, you don't know why you're there, but you always feel, as it were, reassured that…well whatever it is they're doing it's the best thing that can be done at that particular time. **[Patient05_L3]**<br><br>I: Well, about the sort of medicines you have had?<br>R: Well, I remember being very dazed when I first left and quite confused and I kept forgetting quite a lot of things and I gradually got better and my brain came round again.<br>**[Patient06_L2]**<br><br>I: Do you feel that you've been involved in some of these decisions, say you've talked with your mum, and you've talked with the doctors, how do you feel included and involved in what's happening with those?

		R: Well, everyone has always spoken to me about everything that is happening around me and always made sure that I was aware of it, even in a confused state and that way, I was able to make executive decisions with them, but I wanted them to make them with me because of the confused state, if I'm honest. **[Patient06_L2]**<br><br>I: Were you given any, can you tell me about any general information you've been given about your medicines?<br>R: No, I'm not going to lie to you, there was some point where I couldn't, I didn't even know where I was. At that stage, like, I didn't who I was or where I was, at the time. So, you could have said anything to me.<br>I: Right, but have they…<br>R: And I could have believed it, or I couldn't believe it, I don't know.<br>I: Right. So, this is, is this when you were on ICU or is this in the last few days?<br>R: This, the last few days. **[Patient07_L2]**<br><br>I: So, first things, just thinking about the medicines you were given to take after you'd left critical care. Can you talk to me through, tell me about any conversations you've had with people about those medicines?<br>R: Well, I was pretty much out of it actually […] And being told that I'd had an operation which I'd never really even heard of before, and it's, you know, it's going to, certainly in the interim, short term period, going to be quite life changing. So I was a bit dull and just accepting things, but I do recall being in critical care and being told about the morphine and how it's regulated and these little orange and white buttons which I could, if I wanted to, increase it but it wouldn't over-do it.<br>I: Yeah, okay.<br>R: I was told, I think I was told I was going to get some antibiotics and then after that I was feeling very nauseous, so they gave me injection, not an injection, IV paracetamol and IV anti-sickness. **[Patient08_L2]**<br><br>…. in the end I hadn't had enough sleep to make a sensible decision, so I just…in the end I said well, if the consultant things that, let's go home, you know. **[Patient12_L2]**<br><br>R: I mean, I was asleep most of the time, so…<br>I: Right, yeah. I mean, this is the thing, isn't it, people are, I suppose…so, therefore, actually thinking about that, were you being woken up and told, right, we're going to put you on this now or stuff like that?<br>R: Not as far as I know. Because as far as I was aware, I was asleep for something like six days after the operation, and just gradually shuffled towards the door when I could leave for the high intensity place […] But I've got no idea of being there or what happened there or who I spoke to or anything. I was just completely out of it. **[Patient14_L3]**<br><br>That does the feed that goes into the PICC line, because I think that has to be changed every day depending on blood tests and things like that. So he's been coming round every day, but it's usually in the daytime, so I'm not usually there. So just because of that reason, I sometimes do have to ask questions. And to be honest, my mum can remember quite a lot of it because she does tell me, but if there's something that she doesn't quite understand, I do sometimes have to try and find someone to just ask. **[Family15_L3]**

		They did ask…they rang me one evening and asked for my help to talk to her because she was…this was when she was in the HDU, talk to her because she was getting paranoid delusions, so…but they hoped I could calm her down. But I actually found it quite difficult because she…it was full blown. And I offered to go in and…this was about ten o'clock at night, I offered to go in and speak to her in person, but they said they'd ring me back if that was necessary. Anyway, they rang me back at eleven…and said, she'd calmed down, she'd got to sleep and then they'd reduced her dose…I can't quite remember what she was on at that time, the infusion level down. And then she was okay. I mean, I did know what it was at the time but…and I'd not had a lot of sleep myself for the last two days, so…but I am beginning to get things **[Family16_L2]**<br><br>R: And probably from there, I was pretty much out of it, until later on that night. I remember speaking to the missus and me saying, I'm just waiting for them to take me down to theatre. And that was on – let me think – something like – you might be able to check this – something like Thursday the 24th of March. Yeah? […] And I woke up on, I think it was, Easter Sunday. […] I was out pretty much in kind of like from …for about two and a half weeks, basically.[…] Completely… Well, when I say completely out of it, I was heavily sedated, but to the point where I wouldn't have known what kind of medicines or anything they were giving me. **[Family19_L3]**<br><br>I: Right, yeah. So, and within that, so you're sort of at this, sort of stepped back a little bit now, because you're sister's more able to discuss that with the nursing and medical staff […] But are you then following up anything with any questions to people as well?<br>R: I mean I raised, when she was asleep when I went the other day, I did raise to the staff about her memory, that's my main concern is how her memory's been affected. So there's not a lot with regards to her medication that I'm concerned about, so I've kind of left that to her to deal with. But my main concern is her memory, so I've been talking to people about that, more than anything.<br>I: Right, because you said she can't remember, is it…?<br>R: So she's literally, she has a 24 hour window, so she can remember what's gone off in the past, and she knows who everybody is, and she knows of things coming up in the future, but she doesn't know anything we talked about yesterday. **[Family21_L3]**<br><br>R: Well, to begin with, I was in an induced coma, so I was on medication […] so even though I obviously didn't see anybody, the medical staff that treated me, whenever they did anything they would come to my ear and tell me what was happening, and almost keep me involved in everything that was happening. So, when I eventually waked I knew exactly where I was and it wasn't a shock to the system, and I was already aware that I was on certain medications. But what I didn't realise was that…obviously I was on some quite strong medication and when I had been in the coma, I was having some strange dreams. And I didn't fully realise until I'd stopped taking the stronger medication how it had altered, almost altered my personality in a way, because I remember being really quite argumentative with the staff that was trying to look after me, and that's the last thing I ever am. […] actually when I did realise and people explained to me and I was…because my brain…I think there's no brain damage but I think obviously I've been sleep deprived, there's a lot going on, my body's trying to repair itself, it's a lot of information I was given that I didn't necessarily take first time. **[Patient24_L3]**<br><br>I: Yes, indeed. In terms of the medicines you were given after you left critical care, do you know about any changes that have happened to your medicines and do you know why they were changed and what's gone on with that?<br>R: Well, no, because…and I think it's probably been explained to me but like I said my brain…I was…they gave me ketamine for a while, but I now know that on my notes it says I'm allergic to it, I think, but I'm not…I don't think I was, but it just gave me really severe hallucinations. So, I think probably a lot of stuff was explained to me while I was a bit not quite with it. […] But I am now wary about being given anything too strong because it wasn't a nice experience, like hallucinating or whatever. **[Patient24_L3]**

	**Impact of engagement or lack of engagement upon family or patient**	Well, the doctors and nurses have been really brilliant up in the critical unit, and I think once they're in the ward, it's totally different. You see more busy, in and out and whatnot and it's more crowded and it's totally different. My dad was getting brilliant care in the critical care unit, but then once he got into the ward, he was getting more confused, [inaudible 16:01] asking what he would like, and then there's another person coming, and so, his dinner got messed up and everything because too many people. My dad does get confused a lot, so it was that we were there we asked for what meal to have or he did say, it's confusing, too many people coming asking me things and everything. Because the critical care unit was one nurse and one domestic person, yeah. So, it was confusing for him in the ward. **[Family03_L2]**<br><br>R: Well, everyone has always spoken to me about everything that is happening around me and always made sure that I was aware of it, even in a confused state and that way, I was able to make executive decisions with them, but I wanted them to make them with me because of the confused state, if I'm honest.<br>I: Right, okay, that's really helpful and so you feel that there's been a discussion, you feel that…you said you feel you've been included in all the decision-making, has that been important to you then?<br>R: Yes, I like having a sense of control.<br>I: Okay, so do you think it's important to have a sense of control around your medicines you're taking, is that because of what has happened to you?<br>R: Yes, because it shocked me so much when I got carried away, I'm way more strict on that side of things now […] I'm never too late or too early, I'm just on the minute.<br>I: Right, in taking it at the right time? **[Patient06_L2]**<br><br>R: ...actually, I do remember now. I was asked about paracetamol, and I said yes, and I was asked if I wanted it IV or by tablet. And I said, well, IV would work quicker, wouldn't it? And they said, yes. So I said, right, IV please. And it still hadn't come an hour and a half later. […] So I had to actually, physically ask for it. […] Whereas I reckon if I'd have been taking it by pill I could have just been given a little pill box and a cup of water […]<br>I: So actually it delayed things a bit, that?<br>R: Yeah. **[Patient08_L2]**<br><br>I: Is that having...what's good about that sort of communication then, around...?<br>R: Well, just take the stoma nurse, such as, let's face it, this is a, you know, this has been a very concerning week of mine, and she was lovely and I used to have, prior to this, an extremely active life, I might be 71 but I was a fell walker, I've done quite a lot of the Lakeland and Snowdonia peaks in the last seven or eight years. And I don't want that to stop, in fact I'm, to be honest with you, in two weeks I should have been in Anglesey walking some of the Anglesey coastal footpath and the...what's the name of it, what's the name of the mountain in the middle of Anglesey, what do you call it?<br>I: Oh, gosh, can't remember, yeah, go on.<br>R: And all that's on hold anyway. And she said, well, look, I've worked with policemen, rock climbers, fire fighters, and I've got them all back. And that was reassuring, she might have been a little...exaggeration, but, you know, I'm 71 so I don't expect to, you know, be a 40 year old rock climber. But she was reassuring that, you know, it's going to be slow, but I might get there. **[Patient08_L2]**

		R: So I would go in just once a day, but I would stay for several hours. It was a half hour journey into hospital so when Mum was really poorly I would ring…if I rang first thing in the morning about six o'clock, then I would be able to talk to the member of staff who cared for her for 12 hours over the night so that was important to me. Then I would probably ring up about maybe ten o'clock something like that and then I would probably go onto the ward at one o'clock and then stay for a few hours. It depended on how Mum was and then that changed as Mum, kind of like, got better I would come up…you know, and then I'd ring. It was almost a dread, the phone call and I would ring when I got home because you never quite knew what the information [audio breaking up 24:01]. What I found and I found this actually from seeing other relatives as well, we talked about it being an emotional rollercoaster because the staff would give you one piece of information and at that point in time that was accurate but because she was so poorly, in a few hours later it could change. So we got to the stage where we weren't actually letting, apart from immediate family, we stopped letting other people know because it was just exhausting because it was changing from one thing to another that we soon learnt, yes, and to brace ourselves. We, kind of, thought, oh, we were turning a corner, and then you thought, actually, we've still got a really long way to go on this journey. **[Family11_L3]**<br><br>Well, it only sort of became apparent to me some way into the event that the normal blood pressure medications, including the diuretic that I was taking, they all stopped doing operate…I didn't know that at all...that they were stopped. And it was when I kind of realised that I was blowing up a bit like the Michelin man that I kind of had the discussion with one of the doctors I think it was about, you know, whether that had changed. I'm sure…but yes, it's definitely been discontinued, you will go back on them, but it will be a gradual process, and I'm thinking well, the rate this thing is going, you know, I won't be able to get out of the bed, you know, I'll be a barrage balloon.<br>**[Patient12_L2]**<br><br>Well, I mean, I think when it came to discharge, the consultant was basically saying to me…and I'm sure he is a consultant he does know, that you do get post operative swelling, you know, and it'll resolve itself within seven, seven to 10 days but, you know, if you are concerned we can do something about it in terms of, you know, we might want to stay on or we might to think about giving you the diuretics. And then, you know, more like the support nurse thinks…it may be he was thinking more about well, this chap is on his own at home, he's not got a lot of support, he's not got a family in this area and he's only got his neighbours and his friends and it's going to be very difficult for him in this, sort of having this double whammy if you like, of the post operative issues you always have and this oedema. And so, you know, I think he would have definitely wanted me to have some sorting out of the oedema before I went home. **[Patient12_L2]**<br><br>But…yes, if you're trying to pick out a…an area of slight lack of communication, I didn't know why it had been started. I still haven't been told why it was started. I've been given a pack of 28 which I presume we're going to be taking for that time and I don't know when it's going to stop. Well it will obviously stop whenever I run out of that pack and if the doctor says…GP when say…well I will request should she need it. Another member of the public might just ask for it again. The only difference being I will ask, does she need this anymore, can someone check how long she's supposed to be on it. But that's the only difference between me and, say, you know, a non-pharmacist. **[Family16_L2]**<br><br>I: Yeah, indeed, indeed. But you said those conversations were reassuring, you said.

R: Yeah. I feel like on reflection the intensive care nurses do an amazing job. They don't reassure you in the way, they don't say your loved one is going to be fine, because they are open and honest and they will tell you that they can't give you that reassurance, despite how desperate you may be for it. They don't give you any false pretences that your loved one is going to wake up tomorrow and they're going to be fine, because they can't tell you that information, which I understood from the beginning. But I think they reassure you in the way when, like, for instance, he was put on a ventilator and for me that was the worst thing that could happen, just being put in an induced coma, I thought that was meaning he's not going to wake up again sort of thing. I didn't understand the actual process of it. Because you only hear about your friend's aunty who was on a life support machine and they had to turn it off because she was unresponsive. You don't hear about the amazing things that the intensive care people actually do to save lives. So I think when they sat us down and explained the process of what would happen to his body and what medications they'd be using and stuff like that, it did make me feel quite reassured that it was a positive thing as opposed to a negative.
[Family18_L3]

I: From your experiences of the last few weeks generally how do you think family members can be best involved?
R: I think on reflection, I think…I did express this to the nurses, I think for me as the partner having very strict visiting times, I think that's not very good for someone who is in intensive care just because I feel like every hour something can change, and I think if your loved one is having a bad morning, for instance, and you have to leave them because you haven't got a visit booked, I think the best way to keep informed is to keep that conversation…to keep being around, to keep being involved. But I think having a direct line to the room is a really positive thing. You know, you can ring up in the night and they'll tell you how your loved one is and the information that you want to find out. But yeah, I think, throughout my experience, our experience as a family, I feel like they have done an amazing job, and I do feel like we have been informed a lot. I couldn't praise the intensive care staff any more if I tried, to be honest. The nurses have done an amazing job. They're really polite, pleasant. I think it's just been a big shock moving to the ward from that environment. And again it's not a problem. I think it is just because even though without knowing, because they don't reassure you in the way that you want them to reassure you in regards to your family member's going to be fine, I think just getting that information, that knowledge and you feel quite comforted that the nurses are very experienced and they know exactly what they're talking about, I think that's very reassuring in itself. But then, I think when you're on the ward and you don't see a nurse for quite a while and they're really busy and there's a lot going on, it's quite hustle and bustle, I think that then does give you a bit of an uneasy feeling. I think for the first days especially, when they do get taken from an intensive care environment, if they're not still 100 per cent, obviously which they're not going to be, I think it is a bit uneasy for the family. **[Family18_L3]**

I: Yeah, precisely. So there's almost like…well, I'm not saying reluctance. Is that too strong a word? But it's busy I suppose and it's…
R: Yeah, I think in obviously in intensive care that nurse is looking after that one patient. Because of how critical it is they have to know all the information about that patient, what is going on that day, what the doctors think and the stats and everything. So I think because of that they just know and it gives you that reassurance that… Not that the others don't know what they're doing, I don't mean to sound disrespectful, but it gives you the reassurance that your family member's getting looked after by this person who knows what is going on inside out, whereas at the minute for me it's more like the basic care needs are being met. I know that if my partner needs to go to the toilet at night someone is going to assist him. But I feel like in intensive care I knew that if my partner's heart rate plummeted that he would get assisted right then and there. **[Family18_L3]**

I: Have you been given any general information in terms of written information about any of these medicines? Is there anything sort of like a general information sheet, or a summary sheet, or anything like that?

		R: I've had a… They did a diary for me which is being documented every day, you know, what nurses were looking after me, and what happened to me that day, you know, and did I have any ups and downs, that sort of thing […] And that's…[inaudible 0:21:22], kind of thing, gives you quite a lot of information and insights, what was going off on that particular day. […] And I've also had some leaflets, although I can't remember which ones they are or where they are. But I have had some other leaflets. **[Family19_L3]**<br><br>I: And do you think you're getting enough information then as a consequence?<br>R: You never get the full picture with doctors unfortunately. They can be very vague. But you have to ask the right questions otherwise they don't answer properly, which I have found. And there has to be more than one of you because obviously two people have got different points of view, different questions, different sides of questions to ask. So it's easier when there's a couple of you asking questions.<br>I: So you think that's a…yeah, that reluctance is because they think you won't understand or because they are being guarded?<br>R: Possibly. I definitely think a bit more transparency when it comes to these things is needed. I would certainly say that. **[Family20_L2]**<br><br>R: And she was having paracetamol, she'd got a pain in her chest, this was before we knew she'd broken her sternum so they were giving her a paracetamol, and then she was taking paracetamol orally once she'd come round a little bit, because she didn't want any more of the oramorph.<br>I: And so, 'cause the oramorph was making her delirious was it, or…?<br>R: Yes, well I think the after effects of just coming round was, but that just made it a lot worse.<br>I: Yeah. And how do you feel about any of those explanations that you've been given?<br>R: Yeah, it was fine. I think it would have been nice to have spoken to somebody about the oramorph beforehand, but if it's happening in the night, obviously, it's not something they're going to phone up and chat to you about, they're just going to make that decision, which I totally understand. **[Family21_L3]**<br><br>I: So I suppose the next question is – [brief interruption] – in terms of those medicines then, would you have liked to have had conversations with people?<br>R: Yes, definitely, because these last couple of days, these last three days especially, my mood has been very low and I've been very teary. And at first I couldn't understand why, and then I was talking to (name of nurse) this morning and it turns out that I haven't got any alternative or anything, it's just been stopped. **[Patient22_L3]**<br><br>I: In what ways would it have been good to have been involved?<br>R: Well, it's just nice to know what you're taking, what it's for, and why you're not taking what you were taking before. You know, okay, yeah, we understand that you were on an antidepressant before you came in, but this is why we've stopped it, do you think that you might need something to replace it? How has your mood been, is it any better?<br>I: Yeah, absolutely, those sorts of questions then would have helped, would they?<br>R: Yeah.<br>I: How do you think patients can be best involved?<br>R: Talk to us, just talk… **[Patient22_L3]**<br><br>R: Yeah, I feel like everybody's…we've all got one goal in mind and that's to get us better. They will listen if I've got concerns, if they don't have the answer they'll go and find somebody that does. Yeah, it's been a breath of fresh of air being on the ward. Yeah, everybody's very respectful, very mindful that you are an individual with feelings and thoughts. And if you've got questions and, like I say, if they don't have the answers they'll always find out for you. **[Patient24_L3]**

		I: Yes, that's something which a lot of people have said to me as well. Do you think patients…how do you think patients can be involved in decisions around their medicines? What's the best way for them to be involved in that?<br>R: I think it's just telling people what it is, what it does, what it will enable them to do, what side effects there might be, what alternatives there might be. So, it's just having all the information to hand I think and maybe also, especially for the likes of me that wasn't amazingly compos mentis to begin with when I was coming around from the strong medication, having somebody there with them, whether it be somebody from the medical staff, a nurse that could then maybe explain it in slightly different terms or better still a member of family that will retain that information and be able to remind them of the conversations you had. Yeah, having somebody other than just the patient, because I think sometimes we're not always there, sometimes our brains not on…[…] Sometimes we're sleep deprived. On the wards I think we're all just so very sleep deprived. So, it might take a bit more time to get information into our heads than on a regular day anyway. **[Patient24_L3]**<br><br>R: Yeah. And that took several hours it took for me to…and, you know, I'm not one for shouting unless I really have to but I really blew my top, to be honest with you. It got to a point where we'd asked several times, (name) had been so patient. He was, at this point, throwing up, he was in absolute agony. All the progress that he'd made in the ITU had gone. He'd, at one point, asked to switch the machines off and said I don't want to be here, I can't do it, he was in that much agony. And as a mother, to hear your son say that, as you can imagine, that was the point that I blew my top and I demanded to see the consultant. And finally, after hours and hours and hours of waiting, they finally prescribed him…the first thing was paracetamol but then got him something stronger, which was they put him on…I believe was oxycodone which, it was the button that was…and then that started making him sick. **[Family25_L3]**<br><br>R: To be fair, it was a mixed emotion, if I'm being honest. You know, inside I'm broken because my son is in agony and there is nothing that I can do. I'm angry because I know there's people that can do something. I know that they're stretched and I understand and totally appreciate that, however, I just never felt that they were on our side. So it always felt like a bit of a fight. I had a consultant's words to me, when I…because to me, they weren't checking (name), they weren't…I'm very clued up on healthcare, so I work in the healthcare industry. So, you know, they'd done no pressure care with (name), they'd not come in to check for anything. His feet were overhanging off the bottom of the bed, they'd not offered to take him up the bed. There were so many things going on that it just built up over this time period of (name) being in the state that he was in. I said to the consultant, it's institutional abuse, which it was. And he said, I completely agree. And that to me was just like the icing on the cake because then he said, however, we are short staffed. But, you know, for me, how is that the patient's fault? Not just my son, I don't doubt other patients are probably in the same boat, however, you know, it shouldn't be that way for anybody. So it was such a mixed emotion, to be honest. It's quite heartbreaking as a mother to watch your son in that much pain to the point that you feel like you have to blow your top just to be felt like you've been heard. **[Family25_L3]**<br><br>R: To be fair, we had to ask. So we…I personally wasn't made aware by the doctors or the nurses that the feeding tube was being changed to the NG tube, for example. However, (name) was conscious and awake and, you know, I assume that they would have had that conversation with (name). But I don't think it was necessarily a big change because I think they just…instead of using it to pump food down, it actually, sort of, did a bit of reverse a bit and started actually using it for suction of the bile. So in terms of his eating and things like that, you know, they've not been the best at communicating whether (name) can eat and drink. They stopped his fluids the other day. And she just went to walk out and said, oh, (name), we're going to stop fluids now and we'll see how we go. And she just, sort of, went to walk out and I had to stop her and say, can I ask, but why are we stopping the fluids and does this now mean that (name) can drink? You know, before that, you were telling him just enough to wet his mouth, is he now supposed to drink independently, is that the plan? And then what happens if (name) starts to be sick again, what's our next action then, can we plan for both positive and negative so we're prepared? And she said, oh, we'll have to look at that if (name) starts to be sick again. And in my head, I'm thinking, well, that means he's going to be sat there suffering, can we have something in place that's ready, so if it happens, we can act. **[Family25_L3]**

		I: Yeah. You've actually used a phrase there which is I find it really interesting about 'depth of explanation'. You know, is that what would be useful to have that, sort of, further…?<br>R: I think so. I think knowledge is power. I think if you understand what you're facing and what the plan is, you can better prepare for it, you can better understand what to expect for both negative and positive. You don't panic if something bad, you know, goes, if you're prepared for the potential of that. Instead, you get slapped with something negative that happens and actually you think you've just jumped back 10 steps but really in reality, it's quite normal. But you don't know that it's quite normal until it happens, if that makes sense.<br>**[Family25_L3]**<br><br>R: Yeah. We have, yeah. I mean, we go through everything so thoroughly anyway, it really is about taking…for me, I just always say take your time, even if it's an urgent decision, take your time. (name)'s made decisions throughout this whole process and come through to the ward and now does not remember making decisions. So we've had to reapproach them, you know. So everything that we do now, we, sort of, go through with a fine toothcomb. In terms of giving them the feedback, no one's ever really asked us anything about any of it, to be honest, other than just general, oh, was that okay, does that dressing look alright, okay, do you want anything else? No one's ever sat down and said, can I just ask how you're feeling, you know, how do you feel about making that decision, do you feel like you understand that decision? No one's ever done any of that. **[Family25_L3]**<br><br>I: Yeah. How do you think family members can be best involved in this? Because I've spoken to quite a few people now doing this project and we're beginning to build up an idea of what might that role, if you like, be. But what do you think, you know, in terms of when you've had someone being in critical care and then transferred down the ward, how do you think family members can be best involved?<br>R: I think first and foremost, they need to be reminded that it's not going to be like it is in ICU. It was a massive…I want to say downgrade but I don't mean it to sound like it, if that makes sense. But you go from this immaculate care, one-on-one, everybody's got so much time and patience and willing to give you them answers. To, you get transferred down to a ward, half of them don't even seem to know anything about you, don't know what you've been through as a family, as the patient. And then you're re-living every moment again and you're already exhausted emotionally and physically. I think that would make a massive difference is having the staff, you know, understanding the patient before they get there, rather than when they are there. **[Family25_L3]**<br><br>R: I've had conversations about my pain relief. […] About, trying to make it stronger. They've told me, they can't give me anything that's stronger, and I believe that's because I've got renal failure anyway, and I'm not allowed a lot of pain relief medication, because, you know, because of kidney failure […] And it would be dangerous to take anything stronger […] And that makes things more difficult for patients like myself. […] Because we're often sat in pain, and there's nothing, really, we can do about it. And it's very frustrating, and it makes the quality of life more unbearable. **[Patient27_L3]**<br><br>I: Right. And how did you feel about that, then?<br>R: Well, to be quite honest, at the moment, it's just got me more confused. […] And more upset, and I'm just trying to just hold onto the fact, you're on your proper medication now, you're building up your proper allowance, so you might not feel as down, and disheartened, and depressed. […] That's the only way I can deal with it, at the moment. […] I can't get angry; I can't scream and shout about it […] I just need it to be out of the way, I just don't want it to be an additional problem. **[Patient28_L3]**

		R: I was always kept up to date with it. I know that, during the stay, it was quite, with the situation that she was in, there was quite a few different medications that were on and off, yeah. But every single time, they were putting her on something, or they were taking her off something, I was informed. And I was allowed to, like, just ask, any time, really. I was up to date with any progress that was made. As far as like the medication wise, and the general health wise, and I can't really say any more than that, because I mean, they were brilliant, I was just able to ask any time.[…] And being well informed, obviously. Despite, obviously, the situation that we were in, it does put your mind a lot more at ease when you're aware of exactly what's going on, and exactly the medications, and you're kept informed every day, so, it does genuinely help. **[Family29_L3]**<br><br>And because of that, she's ended up having scar tissue, and they would have never really known to put her on the medication, that actually helps her airways, unless I'd have mentioned it. So, I think, when there's an extended, or a potential period in critical care, or even on a general ward, just in my opinion, I feel like it would just be nice to sit down with the family, and ask exactly what she's been on, exactly what the potential emergency scenarios have been, that could be contributing to some of the issues that the patient is having currently. Because it just makes things a lot smoother, really. **[Family29_L3]**
	**Patient health_ severity, fluctuations, post-discharge deterioration**	I: In terms of the medicines as well, thinking about that, have you been given, sort of, general information about those? You were told this, about the heart medicine needing to be balanced out with the kidney medicine but was there some, I don't know, some general information and general explanations around side-effects and that sort of thing?<br>R: Well, I guess, when they had got this water retention they had to say to us, look, because we've had to give her this drug to control her heart this is what's led to this and this is what the kidney doesn't like. Then the sister's explained to us, so we're giving something now to tickle the kidney, I mean, she would have said the name of it but I can't remember [inaudible 09:56] that would have made sense to me, she said, we're now giving another drug to try to encourage the kidney to start working again. **[Family11_L3]**<br><br>So I would go in just once a day, but I would stay for several hours. It was a half hour journey into hospital so when Mum was really poorly I would ring…if I rang first thing in the morning about six o'clock, then I would be able to talk to the member of staff who cared for her for 12 hours over the night so that was important to me. Then I would probably ring up about maybe ten o'clock something like that and then I would probably go onto the ward at one o'clock and then stay for a few hours. It depended on how Mum was and then that changed as Mum, kind of like, got better I would come up…you know, and then I'd ring. It was almost a dread, the phone call and I would ring when I got home because you never quite knew what the information [audio breaking up 24:01]. What I found and I found this actually from seeing other relatives as well, we talked about it being an emotional rollercoaster because the staff would give you one piece of information and at that point in time that was accurate but because she was so poorly, in a few hours later it could change. So we got to the stage where we weren't actually letting, apart from immediate family, we stopped letting other people know because it was just exhausting because it was changing from one thing to another that we soon learnt, yes, and to brace ourselves. We, kind of, thought, oh, we were turning a corner, and then you thought, actually, we've still got a really long way to go on this journey. **[Family11_L3]**<br><br>R: Well, it only sort of became apparent to me some way into the event that the normal blood pressure medications, including the diuretic that I was taking, they all stopped doing operate…I didn't know that at all…that they were stopped. And it was when I kind of realised that I was blowing up a bit like the Michelin man that I kind of had the discussion with one of the doctors I think it was about, you know, whether that had changed. I'm sure…but yes, it's definitely been discontinued, you will go back on them, but it will be a gradual process, and I'm thinking well, the rate this thing is going, you know, I won't be able to get out of the bed, you know, I'll be a barrage balloon. **[Patient12_L2]**

R: And there was also a conversation, a three-way conversation, no…a four way conversation between myself, the upper GI support nurse, the junior doctor and the consultant on the day I was due to go out, and it was a question as to whether I…as far as the consultant was concerned, I was medically fit to discharge. The support nurse, I think, thought that I should stay another night and try and get this oedema sorted out a bit more and be happier before I went home. I wish I'd taken his advice, but in the end I went with the consultant saying, you know, there's no real reason for you to be here and the rest as they say is history. I wished I had stayed because I think the junior doctor was talking about, you know, putting a drip up and getting some diuretics into me, even there and then, but that's history and 28 hours in Northern General is not much fun I can tell you, especially when you spend nine hours lying on a trolley in A&E. **[Patient12_L2]**

R: I think given that I had all the tests again that I'd had in order to escape the first time while I was, you know, going through the A&E process, the unbelievable A&E process, the amount that it costs the NHS to issue that Furosemide prescription must be phenomenal, given that I had a 999 ambulance…but that's a new first as well, I've never been in one of those before…was at the Northern General, all the processes, all the tests, the overnight bed, only for me to say, oh, you know, oh, we think you are…the oedematous yes, and we think you need Furosemide and I said yes, I do, thank you very much and would you like to take it here or go home, and guess what the answer to that was? **[Patient12_L2]**

I: Well, you talked there about, you know, having a conversation with the GP. When you were given this schedule for the plan as it were, for medicines and the reintroduction, who was it who did that in hospital?
R: Excuse me. I did have a…it was one of the ward doctors, I think, who came with the blood pressure medication, would certainly be in that case, who came with the notion of this reintroduction. I can't remember whether that was before or after the oedema showed his head, but…and that was, you know, fine by me at the time. Well, it would have been if I was feeling…it depends, the day before I was discharged when I was feeling on top of the world or the day when I was discharged when I was feeling dreadful. But it was kind of presented to me as, this is how we do it, this is how we reintroduce and I know what it was. But the oedema was apparent and it was only when the oedema became apparent and that's probably…I don't know whether it happened in particular this way, but I think it did, I was walking thinking blimey, my legs are getting bigger and bigger and I was walking around the ward trying to get mobilised [inaudible 0.27.18] walking up and down the corridor thinking, I wonder whether there's a set of weighing scales anywhere around here. Well, there was and my weight before the operation was 82 kilograms, I got on the scales and I'm 91 kilograms. So, I thought well, a) they've cut a lot of me away, b) I haven't been eating and c) this must mean that the rest of the difference is all water, you know. Well, I did mention this to the nurse and I think it was at that stage that the doctor came in and said oh, we will be reintroducing the Spironolactone which is the diuretic straight away, have some of this now. And I don't know if it was the same time, but they did say, I think that, you know, we started the [inaudible 0.28.12], we re-started the [inaudible 0.28.12] at half dose for the tacky. And then the schedule for reintroducing the rest of the Atenolol and the whole of the Lisinopril was on the discharge letter. **[Patient12_L2]**

R: I mean, I was asleep most of the time, so…I: Right, yeah. I mean, this is the thing, isn't it, people are, I suppose…so, therefore, actually thinking about that, were you being woken up and told, right, we're going to put you on this now or stuff like that?
R: Not as far as I know. Because as far as I was aware, I was asleep for something like six days after the operation, and just gradually shuffled towards the door when I could leave for the high intensity place.[…] But I've got no idea of being there or what happened there or who I spoke to or anything. I was just completely out of it. **[Patient14_L3]**

I: Right, okay, right. And you said you had a good explanation about that.

		R: Yeah. We had already been told a lot before it, before she actually had it in, which was on the ward before her surgery, before Critical Care. But the people in Critical Care again explained to us that she'd still be on it for a while because she still can't eat straightaway. […] That's just one detail I can remember. And she was on a lot of other medications which… She's on antiseizure medication as well, which they made sure that they gave her through a canula because she couldn't have it orally, because obviously she couldn't swallow anything. They explained to us about that as well, so they had to change that form of medication to a fluid rather than a tablet form because she couldn't take that. That all got explained to us. **[Family15_L3]**<br><br>R: Yeah. I feel like on reflection the intensive care nurses do an amazing job. They don't reassure you in the way, they don't say your loved one is going to be fine, because they are open and honest and they will tell you that they can't give you that reassurance, despite how desperate you may be for it. They don't give you any false pretences that your loved one is going to wake up tomorrow and they're going to be fine, because they can't tell you that information, which I understood from the beginning. But I think they reassure you in the way when, like, for instance, he was put on a ventilator and for me that was the worst thing that could happen, just being put in an induced coma, I thought that was meaning he's not going to wake up again sort of thing. I didn't understand the actual process of it. Because you only hear about your friend's aunty who was on a life support machine and they had to turn it off because she was unresponsive. You don't hear about the amazing things that the intensive care people actually do to save lives. So I think when they sat us down and explained the process of what would happen to his body and what medications they'd be using and stuff like that, it did make me feel quite reassured that it was a positive thing as opposed to a negative. **[Family18_L3]**<br><br>R: Yeah. Well when we first get there my dad's chances weren't very good. So we were actually pulled into a room, well we were taken into a room, by the consultant and she explained what they were doing in detail. What they were going to try and do, what medications they were going to try and give him to try to stop him getting a chest infection, you know, the antibiotics and everything. She explained all that and she...and then, when he was in critical care, that was straight away, that was as soon as he was transferred to critical care, and then, from then on, we went every day. Me, my sister and my brother went every day and as questions came up, they answered us, they answered them. But they'd already been through the fact that, you know, what they were trying to achieve, you know, in critical [care 00:06:55]. But to be quite honest, they saved his life, to cut a long story short. Critical care saved his life without, you know, without any doubt. **[Family23_L2]**<br><br>But if ever I wanted to ask a question, if ever I was concerned about something or I wasn't sure what was going on, the staff on critical care were amazing and would always take the time to sit down and go through it all with me again. And even though I couldn't communicate either because I had a trachea in that prevented me from speaking, even to the point where the staff they knew that I was getting frustrated because people couldn't lip read particularly well, so they got me a board so I could write on it and communicate that way. So, I feel like…yeah, it was a bit strange because obviously choices were out of my hand to begin with, but when I was communicative and once I had questions, people had all the time in the world to explain to me what was happening. So, I feel like I've had as much communication as I possibly could have.<br>**[Patient24_L3]**

		You said there you feel you've been involved in the decisions, with whom have those…you know the conversations around those decisions…who have you been talking to or who's been talking to you about that? R: Yeah, it's just the nursing staff that administer the medications. Obviously they knew what I was on when I came from critical care and they just, is that working for you? Are you happy with that? Do you want this? Do you want that? So, they asked me every single time, it's not a, here's your medication you must have it. We progressed it a little bit in that speech and language therapists weren't completely happy with my swallow, so rather than trying to swallow tablets now, I get like a…what do they call them? Like an effervescent tablet, isn't it? […] So, everything's liquid form now. But, yeah, it feels like a choice, like it's a, you must take this. This is what it is…I also have an injection in my tummy once a day as well, I think that's a blood thinner because I'm not moving around as much. So, that's an option. There's also an option to have…oh what do you call them when you have the face masks with the…a ventilator, essentially like an inhaler. That's also my decision but they always try and give it me when I've got visitors, so I didn't have that for the first few days. But that was to clear stuff off my chest and every day I feel my voice is stronger. So I had this conversation with the nurses and I think they all tend to agree with me and we'll kind of like come to an agreement that it's not really necessary. So, yeah. **[Patient24_L3]**<br><br>The pain relief…in terms of like, pain relief and medication on that side of things. We had a bit of an issue when he first went from the ITU down to the ward, where (name) had no pain relief, they'd not had the doctors re-prescribe it from the different system. So (name) went almost…I think it was about 15 hours without anything and he was in agony, absolute agony. And when we were asking and asking and asking regarding where is his medication and why has this occurred, all we got was, I don't know. **[Family25_L3]**<br><br>R: Yeah. And that took several hours it took for me to…and, you know, I'm not one for shouting unless I really have to but I really blew my top, to be honest with you. It got to a point where we'd asked several times, (name) had been so patient. He was, at this point, throwing up, he was in absolute agony. All the progress that he'd made in the ITU had gone. He'd, at one point, asked to switch the machines off and said I don't want to be here, I can't do it, he was in that much agony. And as a mother, to hear your son say that, as you can imagine, that was the point that I blew my top and I demanded to see the consultant. And finally, after hours and hours and hours of waiting, they finally prescribed him…the first thing was paracetamol but then got him something stronger, which was they put him on…I believe was oxycodone which, it was the button that was…and then that started making him sick.<br>**[Family25_L3]**<br><br>I: Was there any other changes made that you were aware of at any point in his time there?<br>R: Yeah. So I think he went from [oxycodeine 0:13:22] to oxycodone. The oxycodone is what was making him sick on the ward. And that changed from the ITU to the ward. Because he had the button in the ITU, however, it wasn't affecting him. It was when he got transferred down and it went from oxyco…deine to ..done …that…[…] …he suddenly became very sick. He also had the BiPAP machine on when he first was woken out of intensive care. And that went to the [inaudible 0:14:00] the nasal cannula oxygen as opposed to full BiPAP mask. That was a big change. I think the rest of it pretty much through that has stayed the same. He does have the NG tube for the aspiration, that's a change since the ITU and the ward. That's because he keeps getting a build up of bile in his stomach. That originally was his feeding tube in the ITU and then they changed it to become his aspiration tube.<br>**[Family25_L3]**<br><br>I: Absolutely. And in terms of, sort of, general information you were given around medicines, were you told, sort of, things like, you know, purpose, side-effects, things like that? You know, what were those explanations like, or was it just, you know, well we're putting her on this medicine, or whatever?

		R: I feel like it depended, definitely, on the medication, or the nature of it. Because you see, my mum, before she went in, she does have quite a few different medications.[…] And some of them do end up monitoring COPD and asthma […] some of the medication she was given, due to the nature of what she was going through, she was in an induced coma. And, obviously, within that situation, she couldn't take the inhaler she normally had. **[Family29_L3]**<br>R: It was helpful, yeah, yeah, yes, it was helpful because with (name) being in a non-conscious state, he was unconscious for quite a long time. With the airpipe in his throat, they had to knock him out, he was not very well, shall I say.<br>I: No, no, no. How long has been…'cause he's obviously now done onto the ward.<br>R: He's in renal at the moment. He was in critical care for over two weeks and then they'd taken him and put him in the renal unit which he's been in there approximately about a week. And he is medically better but he can't…he's having problems standing up and walking because he's that weak.<br>I: Yeah, yeah, I suppose, yeah, absolutely.<br>R: He's lost three-and-a-half stone in weight since he's been in there.<br>I: Good grief, in that length of time, gosh.<br>R: In three weeks he's lost three-and-a-half stone in weight.<br>I: It sounds like you've been through it quite a bit, he has, definitely. **[Family30_L3]**
	**Priorities - what matters most_ What information (medicines, care, well-being, diagnosis, prognosis) matters to patients (and F&F)**	R: Basically, I was originally on antidepressants and questionable pain relief and then I got put on safe pain relief and told that the antidepressants definitely weren't working because if I wanted to drink again, it probably…yes.<br>I: Yes, okay, take your time.<br>R: Basically, one of my mum's friends was on antidepressants and she said she didn't really get the urge to drink when she was on them, but I still did. So, it feels as if it wasn't really working well with my body.<br>I: Right, okay, and are they taking you off the antidepressants now or have they talked to you about those?<br>R: It's mostly been advice not to take them, but by choice I'm not going to take them, there is no point taking something if it's not going to work, that's just going to put my liver at further risk.<br>I: Yes, precisely, absolutely so then you've made that decision then?<br>R: Me and my mum together. **[Patient06_L2]**<br><br>I: Is that having...what's good about that sort of communication then, around...?<br>R: Well, just take the stoma nurse, such as, let's face it, this is a, you know, this has been a very concerning week of mine, and she was lovely and I used to have, prior to this, an extremely active life, I might be 71 but I was a fell walker, I've done quite a lot of the Lakeland and Snowdonia peaks in the last seven or eight years. And I don't want that to stop, in fact I'm, to be honest with you, in two weeks I should have been in Anglesey walking some of the Anglesey coastal footpath and the...what's the name of it, what's the name of the mountain in the middle of Anglesey, what do you call it?<br>I: Oh, gosh, can't remember, yeah, go on.<br>R: And all that's on hold anyway. And she said, well, look, I've worked with policemen, rock climbers, fire fighters, and I've got them all back. And that was reassuring, she might have been a little...exaggeration, but, you know, I'm 71 so I don't expect to, you know, be a 40 year old rock climber. But she was reassuring that, you know, it's going to be slow, but I might get there. **[Patient08_L2]**<br><br>I: Yes, absolutely, and because things change that makes it more difficult. You said there you used the phrase, important to me, and I'm just thinking, what's most important to you about your mum's medicines or about supporting her with her medicines, if you like? What's the most important thing? Is it about…? I'm also really intrigued that you're not interested in the names of the tablets, which is fascinating. What's the most important thing about the medicines for your mum?

		R: I guess, I've just trusted the doctors in that the drugs that they're giving her now haven't got any long-term negative effects in which case I would have expected to have been informed on that, if there was a choice. I guess, if it's a life changing drug and there's no other option, I wouldn't be particularly bothered if that had been…if my mum had received something like that. If it was something that there was a choice with, you know, and it would affect her quality-of-life long term, you know. Does that make sense? **[Family11_L3]**<br><br>I: …whereas where there are things you've got to make that decision about. Actually over the whole thing you've talked to me about, the other question I was thinking of there is what's been the most important thing to you anyway, throughout all this?<br>R: I guess, as a relative, I think the personal care that…I think I just take for granted that higher up the doctors know what they're doing, they know what they're doing with the medical care but for me, it's the personal care that my mum's being washed daily, that they're taking time with her hair, that they're talking to her as she's being given the medicines. That was quite interesting, which staff, even when my mum was really heavily sedated which staff when they were putting things in her line were actually talking to her and saying, (patient's name), I'm just going to do this now, (patient's name), I'm just doing that now, I'm just going to do this now, (patient's name), I'm just going to do blah, blah, blah, that was really important to me and not every member of staff did that. Actually it's really reassuring as a relative to actually know, this is what I'm doing now, this is why I'm…do you know. **[Family11_L3]**<br><br>Pretty much constantly I had a critical-care nurse, so dedicated one-on-one nursing. Sometimes the doctor would come in and say, are you in any pain; and you'd go, oh, yeah, it's quite…right bad at the moment. And they'd talk about what was the best thing to give me, between themselves, and then say, we're going to give you a shot. But I didn't think to remember what they were going to give me, do you know what I mean? […] At that point you're just kind of thinking, no, just give me something to take pain away, you know. **[Family19_L3]**<br><br>I: And would you, I mean, what drives that, just get on with it, is it, do you trust them to just do the right thing? Or is it that you, at the moment, things are just too much, and you don't want to be dealing with other things?<br>R: It's too much, I can't be doing with it, I just can't. I can't…I: Yeah. So, you…go on, sorry, carry on.<br>R: I can't be doing with it. In fact, actually talking, and trying to recollect this now with you, it's putting a great strain on me, because everything is mixed up in my head […] And that is the problem.[…] And I don't know if other patients have experienced that. You can't focus, you can't get clear, you can't see a clear path through. And even discussing it with you, now, you can't see a clear problem. **[Patient28_L3]**<br><br>I: Right, okay. And does that mean you've had to take more on in talking to the doctors and nurses then?<br>R: Yes, I've had to take more on what with (name) being on his own, a lot more own. Because me and my wife are finding it hard because we've got my aunty to look after who's sick and bedridden and we're up here, up at hospital and my son is living in assisted accommodation so we have to see to him as well [inaudible 15:09].<br>I: Yeah, it is, it's hard work, isn't it, with all that's going on, it's not easy for family members, is it, in these situations. R: Yeah.<br>**[Family30_L3]**
	**Problems with medicines - Patient perspectives and how that's communicated**	R: …actually, I do remember now. I was asked about paracetamol, and I said yes, and I was asked if I wanted it IV or by tablet. And I said, well, IV would work quicker, wouldn't it? And they said, yes. So I said, right, IV please. And it still hadn't come an hour and a half later. […] So I had to actually, physically ask for it. […] Whereas I reckon if I'd have been taking it by pill I could have just been given a little pill box and a cup of water.<br>I: Yeah. I see, because they would have had to make the bag up, presumably.<br>R: Yeah.<br>I: Yeah, absolutely. So actually it delayed things a bit, that?<br>R: Yeah. **[Patient08_L2]**

I: R: Up until last night I was all over the place, one night on intensive care, I take statins and they gave me three tablets when I used to take one. I asked them that, the nurse said that's what you've been prescribed, but it turned out that they'd overdosed me because when the nursing team did the handover in the morning, the nursing staff queried that, why did she give me that amount, and somebody had misread the amounts on the tablets.
I: So there's a bit of inconsistency there as well, isn't there, then?
R: Yes.
I: Both in what you were being told and what you were being prescribed.
R: Yes.
I: How do you feel about that inconsistency?
R: I didn't feel safe, to be honest. I mean, it was a junior nursing staff, two junior nurses on shift at that time, and I thought I don't want to spend another night on here.
I: Right, okay. And how did you feel in the morning when they said, oh, no, it's better, or was that when you realised that there'd been…?
R: That's when I realised, you know? […] No, as I say, I queried it at the time, and they said that's what's been prescribed to me, but I felt a bit aggrieved that I'd been overdosed prescription-wise because somebody had misread the quantity or the size of the tablet. **[Patient10_L2]**

R: Well, they've not mentioned it. I found it in the bottom of the bag.
I: Right, okay. But no-ones told you any reason why you're on this.
R: Nobody told me they were going to put it in, and no, I've no idea what it's for, but it has got my name on the packet.
I: Right. So, it's for you, it's the right one. What do you feel about the fact that no-ones talked to you about that?R: I would have preferred if they had.
I: Right. And do you think you might try and find out from someone or…?
R: I'll try and find out what it's for before I use it.
I: Yeah, okay. There should be the information leaflet in there, shouldn't there, which might help, I suppose.
R: Yeah.
I: So, you would've preferred…?
R: I'd forgotten all about it.
I: Yeah. You would have preferred to have known why…?
R: Yeah. Well, it's slightly disturbing just to find this thing at the bottom of the bag and wonder what it's for, but I did check it and it was for me. **[Patient14_L3]**

I: Yeah, precisely. So it's more of a welfare thing at the moment, you're getting the IV, you're getting the medicines, you're getting better, but perhaps not that same sort of one to one as it were. Have there been any problems with his medicines at all, particularly in this change from critical care down to the ward, that you're aware of?
R: No, I don't think there's been any problems. I think the only slight problem that there was is because he's on a reducing dose of clonidine, the second day I think he needed the reduced dose, he's supposed to have two in the morning, one in the afternoon and one in the evening, and that's been the schedule, but they didn't have any and they had to order it in from the pharmacy, so that resulted in his second dose being, I think, six hours late. Whereas in the intensive care, they have their own pharmacy on the ward so obviously that doesn't happen. But apart from that, that's been the only little hiccup. **[Family18_L3]**

I: Yeah, indeed. I think, (name) I'm coming to the end of the questions that I need to ask you. One final thing, apart from what we've talked through, have there been any other problems with his medicines in terms of anything that's gone wrong as it were or anything?

R: No. I think the medicines that he's been given have…apart from he had a few nose bleeds from the blood thinners, which is understandable. That is the only thing, but I don't think that's something that goes wrong. I think that's a very, very common side effect of a blood thinner. But other than that, no. Everything's been spot on. Everything that he's been given so far is doing the job it's supposed to. **[Family20_L2]**

I: So I suppose the next question is – [brief interruption] – in terms of those medicines then, would you have liked to have had conversations with people?
R: Yes, definitely, because these last couple of days, these last three days especially, my mood has been very low and I've been very teary. And at first I couldn't understand why, and then I was talking to (name of nurse) this morning and it turns out that I haven't got any alternative or anything, it's just been stopped.
I: Right, okay. So do you know when they took you off that, was it before you were in critical care or has that been since?
R: No, it wasn't, because the only place I've come to since I've had my cardiac arrest was critical care, so they must have stopped it in there.**[Patient22_L3]**

I: Right, yeah. So they stopped his pain relief…his pain relief was in critical care but then when he got transferred to the ward, they'd stopped it but didn't restart it?
R: Yes.
I: I see.
R: Yes. So I think the way that it was explained is that they have a different system in the ITU. So once (name) was moved from one ward to the other, it all needed re-prescribing onto this new system, but it hadn't been done before we left the ITU. However, nobody communicated that with us and I'm assuming between themselves as well, until (name)'s pain relief had ran out from the one that he currently went down with. And it was only when (name) started to experience pain and we asked what pain relief had he got and they said he'd not been prescribed any. So they were unable to give him anything, they couldn't even give him paracetamol.
I: Right. Oh, because nothing had actually been put on the system as prescribed?
R: Yeah. **[Family25_L3]**

R: Yeah. And that took several hours it took for me to…and, you know, I'm not one for shouting unless I really have to but I really blew my top, to be honest with you. It got to a point where we'd asked several times, (name) had been so patient. He was, at this point, throwing up, he was in absolute agony. All the progress that he'd made in the ITU had gone. He'd, at one point, asked to switch the machines off and said I don't want to be here, I can't do it, he was in that much agony. And as a mother, to hear your son say that, as you can imagine, that was the point that I blew my top and I demanded to see the consultant. And finally, after hours and hours and hours of waiting, they finally prescribed him…the first thing was paracetamol but then got him something stronger, which was they put him on…I believe was oxycodone which, it was the button that was…and then that started making him sick. **[Family25_L3]**

I: Right, okay. He had control over that, is that what you're saying, with the button?
R: Yes, it was a button. It had…every time he pressed the button, it would release so much of a dose. Not enough that (name) could overdose, it was controlled, they were very clear on that. So even if (name) pressed it before the timeframe ran out, he wouldn't ever get the dose unless the time had passed. But that began to make him really sick. And that again, we had complications with. So trying to get them to change the oxycodone onto something that wouldn't make (name) sick was a bit of a lengthy progress…sorry, I can't even get my words out now… process again. So eventually, he got swapped to morphine and that's been a more successful pain relief for (name). Since then, they've been very good at administering that as and when required. **[Family25_L3]**

		I: When you talked about having to go to the consultant and be fairly forceful, as it were, you know, what was that like, what did you feel about having to do that?<br>R: To be fair, it was a mixed emotion, if I'm being honest. You know, inside I'm broken because my son is in agony and there is nothing that I can do. I'm angry because I know there's people that can do something. I know that they're stretched and I understand and totally appreciate that, however, I just never felt that they were on our side. So it always felt like a bit of a fight. I had a consultant's words to me, when I…because to me, they weren't checking (name), they weren't…I'm very clued up on healthcare, so I work in the healthcare industry. So, you know, they'd done no pressure care with (name), they'd not come in to check for anything. His feet were overhanging off the bottom of the bed, they'd not offered to take him up the bed. There were so many things going on that it just built up over this time period of (name) being in the state that he was in. I said to the consultant, it's institutional abuse, which it was. And he said, I completely agree. And that to me was just like the icing on the cake because then he said, however, we are short staffed. But, you know, for me, how is that the patient's fault? Not just my son, I don't doubt other patients are probably in the same boat, however, you know, it shouldn't be that way for anybody. So it was such a mixed emotion, to be honest. It's quite heartbreaking as a mother to watch your son in that much pain to the point that you feel like you have to blow your top just to be felt like you've been heard. **[Family25_L3]**<br><br>I: Was there any other changes made that you were aware of at any point in his time there?<br>R: Yeah. So I think he went from [oxycodeine 0:13:22] to oxycodone. The oxycodone is what was making him sick on the ward. And that changed from the ITU to the ward. Because he had the button in the ITU, however, it wasn't affecting him. It was when he got transferred down and it went from oxyco…deine to ..done …that…[…] he suddenly became very sick. He also had the BiPAP machine on when he first was woken out of intensive care. And that went to the [inaudible 0:14:00] the nasal cannula oxygen as opposed to full BiPAP mask. That was a big change. I think the rest of it pretty much through that has stayed the same. He does have the NG tube for the aspiration, that's a change since the ITU and the ward. That's because he keeps getting a build up of bile in his stomach. That originally was his feeding tube in the ITU and then they changed it to become his aspiration tube. **[Family25_L3]**<br>I: … for both the patient and for relatives, I think. Apart from the pain medicine, were there any other…or have there been any other problems with his medicines whilst he's been there or particularly around that transfer?<br>R: No, not really. As for the rest of them, they've been fine. It was more his…to be fair, they stopped everything on the day, he'd not got his fluids that day, he'd not got his pain relief, his anti-sickness. He'd literally got nothing. Like I say, he'd not even got paracetamol, nothing. The room that he went to has got a massive heat pipe radiating heat off it, it's like a little boxroom type thing. It's still on a bay but his bay has got this heat pipe inside it just generating heat. And he was overheating, and he already had a temperature. And then they put him in this bay and then didn't give him any medicine at all. And it's things like that, you just think really, you know? And he can't drink, you're not saying he's not allowed to drink but yet you've not given him no fluids, that's how long without fluids? Then the pressure kicks in. Then seeing your son deteriorate on top of major surgery and the life-threatening experience that you've just gone through and thought you'd come out the other side, it's so very overwhelming. **[Family25_L3]**

		Right. Now, the only thing that I have against this is, when I went into the hospital, I brought in, I've got, you know how you get your prescription from the doctor? […] Well, what I did, I thought, right, well they want prescription medicine, so I took part of all my prescription medicines, from what I got from the GP, in with me, along the prescription list. […] And I said, everything is in that bag.[…] Now, I don't quite know the circumstances, and the events surrounding the circumstances, however, let's just say that, who allocated that medication, I don't know. […] Now, this is the confusing part for me. Some of the medication was in my travel bag, and they got my inhalers from there. […] And a couple of days after I thought, well I have been, in effect, taking medication, but I've not been taking my antidepressants, as far as I'm aware, since I came into hospital. […] So, when the pharmacist did come round, you know, and have a look at what was happening, you know, and checking your medication, I drew it to their attention, and straightaway, they put me back on the antidepressants, which I'd been on for years. […] Now, when I first came down here, you can imagine, I felt shocking […] Because, you see, as well as everything that had happened, I've not been taking the antidepressants that I take year in, year out. It was only when I challenged them, that they discovered they'd not been prescribing them to me. **[Patient28_L3]**
	**Treatment burden - Emotional, psychological (e.g. overwhelmed) and physical (e.g. pain)**	R: Basically, I was originally on antidepressants and questionable pain relief and then I got put on safe pain relief and told that the antidepressants definitely weren't working because if I wanted to drink again, it probably…yes.<br>I: Yes, okay, take your time.<br>R: Basically, one of my mum's friends was on antidepressants and she said she didn't really get the urge to drink when she was on them, but I still did. So, it feels as if it wasn't really working well with my body.<br>I: Right, okay, and are they taking you off the antidepressants now or have they talked to you about those?<br>R: It's mostly been advice not to take them, but by choice I'm not going to take them, there is no point taking something if it's not going to work, that's just going to put my liver at further risk.<br>I: Yes, precisely, absolutely so then you've made that decision then?<br>R: Me and my mum together.<br>I: Right, okay, that's good and…<br>R: I was also prescribed them for anxiety, not depression and they don't really take the anxiety away. **[Patient06_L2]**<br><br>I: Right, okay, that's really helpful and so you feel that there's been a discussion, you feel that…you said you feel you've been included in all the decision-making, has that been important to you then?<br>R: Yes, I like having a sense of control.<br>I: Okay, so do you think it's important to have a sense of control around your medicines you're taking, is that because of what has happened to you?<br>R: Yes, because it shocked me so much when I got carried away, I'm way more strict on that side of things now.I: Yes, absolutely.<br>R: I'm never too late or too early, I'm just on the minute.<br>**[Patient06_L2]**<br><br>I: Right, have you, so you've not been taking…R: And my water tablets. They've given me some extra water tablets, they gave me some more 'cause I've got so much water in my chest which I'm not supposed to have. That's for my heart and they're changing this, they're changing that and it's just getting a bit over the, too much for me [voices overlap 06:19]. I: Okay. So, you, they have been changed but you haven't, you don't really know why or the reasons for them? Do you understand any of the reasons why these medicines have been changed?<br>R: I understand some of them but I'm not 100 per cent sure and at this moment in time, I just want to listen to them, become better and go home. **[Patient07_L2]**<br>I: Right, okay. Do you think, has there been anybody in hospital who's talked to you about them? This plan?<br>R: The hospital, I've got to admit, they've done alright, they've done, they've tried to explain it to me, though, in the way that they see fit. But sometimes when they try to explain to you, it seems like they're talking to you, like you're a little child.

		I: Oh, right. Okay. What does that make you [voices overlap 11:07], yeah, what does that make you feel?<br>R: It upsets you, it gets you angry at first. Then you just, you think to yourself, they must think, I just think, why are they talking to you like this? Then you just talk to the wife. The wife settles you down and that's how it was in my family. I over-exaggerate. I get angry and then the wife calms me down. **[Patient07_L2]**<br><br>I: And to have that knowledge about that, yes. That's interesting, actually, how you've been, and I'll pick something up about that in a bit, actually. Do you know what the current plan is for your medicines?<br>R: Up until last night I was all over the place, one night on intensive care, I take statins and they gave me three tablets when I used to take one. I asked them that, the nurse said that's what you've been prescribed, but it turned out that they'd overdosed me because when the nursing team did the handover in the morning, the nursing staff queried that, why did she give me that amount, and somebody had misread the amounts on the tablets.<br>I: So there's a bit of inconsistency there as well, isn't there, then?<br>R: Yes.<br>I: Both in what you were being told and what you were being prescribed.<br>R: Yes.<br>I: How do you feel about that inconsistency?<br>R: I didn't feel safe, to be honest. I mean, it was a junior nursing staff, two junior nurses on shift at that time, and I thought I don't want to spend another night on here. **[Patient10_L2]**<br><br>R: Yes, and I don't think I probably asked searching questions. It's a very strange thing when a relative is so seriously…and they'd say to me, is there anything you want to ask, and you've almost got…and I almost…as the weeks went by because she was in there for some time, I would almost say, is there something I should be asking? I guess, they don't know how much information to give to relatives so I don't know that I always ask the most pertinent of questions. You know, you're just kind of like surviving day by day really. **[Family11_L3]**<br><br>R: Yes, so Mum was admitted on the 26th for her operation to take place on the 27th and that was like…I can't always get the medical terminology right but it was to replace one of the valves in her heart and so she was expected to stay in hospital between five to seven days. That's obviously if everything happened really smoothly and she was like on CICU for, oh, it must have been at least a fortnight. So this was like the longest possible time and with a breathing tube down for an awful long time. So when they took it out, she just couldn't cope so then they put it back in again and so most of the time she was heavily sedated which is why there was all this messing around with drugs and everything. Then they took it out again and again, she just struggled, and her heart was all over the place and because she was then panicking because she couldn't breathe, that then set her heart off and they were just playing this balancing game all the time and she was like gasping for breath. So then after that they put a tracheotomy in and I have to say from then onwards we didn't really look back. She then went from there onto critical care and her path for that from, I think, she had it in and six days later it was kind of out again because they transferred her with like a voice box [06:53] or whatever you call it.<br>I: Right, okay. So she's had quite a journey of it, really, hasn't she?<br>R: She's had quite a journey, yes, bearing in mind presumably you've got her age, you know, she's 79. **[Family11_L3]**

		One thing that I do notice is that when they do do their rounds, she likes me to be there and fortunately I have been there because they do their rounds in the morning. This is only what happens on this ward and they tend to be a team of people that come. So you've got your main consultant and you've got presumably your trainee doctors and so they all kind of like arrive on troupe because they're kind of like learning and she almost panics and says to me, (name) you will stay in the room. I think it's just…my mum's always been very fiercely independent, she's a retired teacher and she's very articulate and things like that, I think she just finds it a little bit overwhelming now and wants me to be there to listen to possibly ask the questions, you know. Yes, I don't know. **[Family11_L3]**<br><br>I: Again, that's interesting, isn't it, because maybe they…had they start…as you say, because that could have been this morning, could have been them restarting those?<br>R: It could have been, yes. I know obviously on CICU when she was in there for all that time and they were struggling with her heart, I know that the other thing that they were struggling with was her blood pressure so all the time she was having drugs to control the blood pressure and the heart and again, I was very much aware…I didn't know the names of drugs but I was very much aware that they were trying to balance these drugs and the side-effects. Also I was aware when they did sedation release on Mum and I would say to Mum, Mum, are you in pain and then she could, kind of, squeeze my hand and tell me. I think this might have been at the time when the tube was out. It all gets a bit confusing because I remember asking the nurse and saying, can she have pain relief? I remember them giving her pain relief. I also remember asking a few hours later and I remember them saying to me, oh, she can't have any more pain relief at the moment because we've got…she can't have too much pain relief because it would set off something else and I really can't remember what that was but I was very aware that they were playing a balancing game all the time. **[Family11_L3]**<br><br>I: One of the things you've talked through this is saying you're very prepared to ask questions, but do you think the prompt should come from…should it be down to the patient or the family members to say, well, what's happening? Is it actually better if it comes down and they say, what would you like to know or do you know?<br>R: I felt that when I was on ICU that I got the right information. Yes, I think I did because I think emotionally at that point there's probably only so much that you can cope with when you've got somebody you love so much that is in so much…I felt that what they gave me was just about probably what I could absorb. I probably…now, you know, and I guess only because of us having this conversation about my mum saying, oh, what are these tablets I was having this morning, so I guess, maybe, I don't know, maybe more information now. I'm not sure. I don't know really but, no, I think during her time in ICU, I thought the staff had got that right, I do. **[Family11_L3]**<br><br>R: So I would go in just once a day, but I would stay for several hours. It was a half hour journey into hospital so when Mum was really poorly I would ring…if I rang first thing in the morning about six o'clock, then I would be able to talk to the member of staff who cared for her for 12 hours over the night so that was important to me. Then I would probably ring up about maybe ten o'clock something like that and then I would probably go onto the ward at one o'clock and then stay for a few hours. It depended on how Mum was and then that changed as Mum, kind of like, got better I would come up…you know, and then I'd ring. It was almost a dread, the phone call and I would ring when I got home because you never quite knew what the information [audio breaking up 24:01]. What I found and I found this actually from seeing other relatives as well, we talked about it being an emotional rollercoaster because the staff would give you one piece of information and at that point in time that was accurate but because she was so poorly, in a few hours later it could change. So we got to the stage where we weren't actually letting, apart from immediate family, we stopped letting other people know because it was just exhausting because it was changing from one thing to another that we soon learnt, yes, and to brace ourselves. We, kind of, thought, oh, we were turning a corner, and then you thought, actually, we've still got a really long way to go on this journey. **[Family11_L3]**

		R: And there was also a conversation, a three-way conversation, no…a four way conversation between myself, the upper GI support nurse, the junior doctor and the consultant on the day I was due to go out, and it was a question as to whether I…as far as the consultant was concerned, I was medically fit to discharge. The support nurse, I think, thought that I should stay another night and try and get this oedema sorted out a bit more and be happier before I went home. I wish I'd taken his advice, but in the end I went with the consultant saying, you know, there's no real reason for you to be here and the rest as they say is history. I wished I had stayed because I think the junior doctor was talking about, you know, putting a drip up and getting some diuretics into me, even there and then, but that's history and 28 hours in Northern General is not much fun I can tell you, especially when you spend nine hours lying on a trolley in A&E.<br>I: Right, so that's what happened when you went back?<br>R: Yes. **[Patient12_L2]**<br><br>R: I think given that I had all the tests again that I'd had in order to escape the first time while I was, you know, going through the A&E process, the unbelievable A&E process, the amount that it costs the NHS to issue that Furosemide prescription must be phenomenal, given that I had a 999 ambulance…but that's a new first as well, I've never been in one of those before…was at the Northern General, all the processes, all the tests, the overnight bed, only for me to say, oh, you know, oh, we think you are…the oedematous yes, and we think you need Furosemide and I said yes, I do, thank you very much and would you like to take it here or go home, and guess what the answer to that was? **[Patient12_L2]**<br><br>R: Well, living on my own as I do, obviously I did have a lot of trepidation about the operation I was having because it is a big one.<br>I: Right, yes.<br>R: But when I was getting over it and, you know, getting mobilised and what have you, I felt really, really good, then I was, you know, walking up and down the corridors and all sorts. And then slowly but surely this oedema came on and it suddenly overtook the kind of sense of recovering of the rest of it. Well, I began to get a bit worried that I wouldn't be able to… I mean the day before I was discharged, the support nurse came, one of the support nurses came and said, you won't need carers at home because the option had been kind of setup, although it was only phone us if you need us and I said, no, I won't, I can tell that. And then by the next day, I wasn't entirely certain whether I would or not. When I arrived home, it was an absolute challenge when I arrived home, because it was, like, you know, everyone [inaudible 0.16.01], not only being half out of it, but also having an extra hand tied behind your back by the extra fluid which is about a stone and a half of water. So, you know, then I was very worried really, I thought I do really need, you know, I'm going to be struggling this much, that's why I went back, went as soon as I possibly could to the GP to try and get something sorted out. **[Patient12_L2]**<br><br>I: Yeah, right. Do you think that could have been better, or different, if they'd been more forthcoming, if you like?<br>R: Well, I don't know. Because sometimes, it can be rather frightening, at first […] Because he was covered with wires, and everything, and he'd got drips all over. And at first, it were overwhelming, the first night he was took in.[…] And I think, if they did tell us upfront, then, it wouldn't have gone in. **[Family17_L2]**<br><br>R: Because the first couple of days, it were overwhelming […] Because I'd never been in critical care, to see what was happening.<br>I: No, no, and it's a very different environment, isn't it?<br>R: Yeah, it's traumatic when you first see it.<br>**[Family17_L2]**<br>I: Yeah, yeah. And how do you feel about that (name), that you're the one that's having to do that, that asking?<br>R: In a way, I ask the questions that worries me. If they actually told me what was happening, I think I'd be inundated with knowledge.<br>I: Yes, it would be…R: And I probably would get confused.<br>**[Family17_L2]**<br>R: Well, I've never been in an intensive care unit.

		I: No, no.<br>R: And so, when we got there that early hours of morning, my daughter and I, it was just overwhelming. A doctor came out that night and told us what was happening, and why was he put in there. And he told us that, like, 48 hours, either way, you know. **[Family17_L2]**<br><br>R: But obviously I think in those sort of situations when it is your loved one and it is very intensive and there's a lot going on, a lot of different information and every day's different, it is a lot to take in anyway for anyone, isn't it? **[Family18_L3]**<br><br>I: Yes. That's really interesting actually because this is not something which…it's something I've heard before in some respects. So actually I'd like to explore a bit. In what way does the amount of information you're getting…was it right or was it too much or was there not enough? Or is there a limit to how much you can absorb in some respects?<br>R: Yeah, I think the problem is when somebody's in critical care every day is different. I think the nurses did a very good job on making me understand the process of intensive care and the fact that one day he could have a really good day and it seemed very positive but then the next day something could happen it could be not a very good day. But they made me feel very reassured in letting me know that is very common. A lot of the nurses all use the same phrase which is two steps forward, three steps back, and that gave us a bit of reassurance that when he did have a bad day it was just a bad day and tomorrow's a new day sort of thing. So I think that was very helpful. But I think it is a lot of information and I think when he was on maybe six or seven different medications a day on IV drips, he was on dialysis machine, ventilator, he was on a lot of different things, a lot of different medicines, they kept trying different antibiotics. So I think every day there was a lot of information and there was always something that had changed or something new that needed to be added or this needed be up a few milligrams and this needed to be reduced. It is a lot to take in. **[Family18_L3]**<br><br>Yes, she actually asked loads of questions. If I'd have been awake, they would probably have put a piece of tape across my mouth because I'm so nosy, I've got to know everything [voices overlap 0:14:28] […] Do you know, I'm…[so once 0:14:33] I'm completely out of it, I was incapacitated to a point where I couldn't ask questions. […] But they gave as much information to (name of patient's spouse) as I think, you know, she could take in. **[Family19_L3]**<br><br>R: I mean, what do they give you in critical care, because the hallucinations, the…it gets you to the point I never want to step foot in that ward ever again.<br>I: No, I think it's also I think…I mean, I don't know whether that would be the medicines or just being ill and all sorts of things, I know it can sometimes be very difficult for people, can't it, being in critical care, so it is [inaudible 19:06]…I understand it's really challenging for patients to have been through that. One of the things…<br>R: The hallucinations are horrendous<br>I: Yeah, right, right. Do you think that was down to the medicines or do you think that was down to just being unwell or…?<br>R: I've no idea.<br>I: You don't know. Has anyone talked to you about that?<br>R: I couldn't – no.<br>I: So no one's discussed the…<br>R: No. […] They just laugh at you, because I dialled 999 and told police that I was being held against my will.<br>I: Oh right, right, yeah.<br>R: I was…to the point where my sister took my phone off me and I've only just been allowed it back, because I was proper cuckoo. **[Patient22_L3]**

R: Yeah. Well when we first get there my dad's chances weren't very good. So we were actually pulled into a room, well we were taken into a room, by the consultant and she explained what they were doing in detail. What they were going to try and do, what medications they were going to try and give him to try to stop him getting a chest infection, you know, the antibiotics and everything. She explained all that and she...and then, when he was in critical care, that was straight away, that was as soon as he was transferred to critical care, and then, from then on, we went every day. Me, my sister and my brother went every day and as questions came up, they answered us, they answered them. But they'd already been through the fact that, you know, what they were trying to achieve, you know, in critical [care 00:06:55]. But to be quite honest, they saved his life, to cut a long story short. Critical care saved his life without, you know, without any doubt.
[Family23_L2]

R: [Inaudible 00:11:01] you see, the thing is, because he was heal...I mean, when my mum died, like I said, she had advanced Alzheimer's, it wasn't a shock. But when you're somebody...is involved in an accident, to a certain extent you go into shock as well, your body goes into shock. I mean, I started shaking after I had the conversation with the paramedic, you know, because that's how you react, it's not something you're expecting to hear, [voices overlap 00:11:30].
I: No, absolutely.
R: You, yourself go into shock, for a little while anyway, once you start processing what's happened. **[Family23_L2]**

R: But if ever I wanted to ask a question, if ever I was concerned about something or I wasn't sure what was going on, the staff on critical care were amazing and would always take the time to sit down and go through it all with me again. And even though I couldn't communicate either because I had a trachea in that prevented me from speaking, even to the point where the staff they knew that I was getting frustrated because people couldn't lip read particularly well, so they got me a board so I could write on it and communicate that way. So, I feel like…yeah, it was a bit strange because obviously choices were out of my hand to begin with, but when I was communicative and once I had questions, people had all the time in the world to explain to me what was happening. So, I feel like I've had as much communication as I possibly could have.
I: And you said they facilitated even when you couldn't speak with the board as well, which is fascinating, isn't it? Very helpful obviously.
R: It was such a small gesture that was made by…I think…she wasn't a nurse she as one of the support workers, such a simple gesture, I just said to her before I left critical care unit, I know it took you minutes to go and find this thing but, I said, you made a world of a difference, because I didn't realise…obviously my role in a job and I'm also a volunteer, there's a lot of communication involved, and I didn't realise how restricted it is when you can't just talk. And that simple gesture of her taking two minutes to go and find that board to give to me, it made…well, it made my month basically, it gave me my month back and I just said to her, that was amazing and thank you so much, you have no idea the difference you've made. I definitely let everybody know on the ward that they did fantastic. **[Patient24_L3]**

I: Yes, that's something which a lot of people have said to me as well. Do you think patients…how do you think patients can be involved in decisions around their medicines? What's the best way for them to be involved in that?
R: I think it's just telling people what it is, what it does, what it will enable them to do, what side effects there might be, what alternatives there might be. So, it's just having all the information to hand I think and maybe also, especially for the likes of me that wasn't amazingly compos mentis to begin with when I was coming around from the strong medication, having somebody there with them, whether it be somebody from the medical staff, a nurse that could then maybe explain it in slightly different terms or better still a member of family that will retain that information and be able to remind them of the conversations you had. Yeah, having somebody other than just the patient, because I think sometimes we're not always there, sometimes our brains not on…
I: No, that's really interesting.
R: Sometimes we're sleep deprived. On the wards I think we're all just so very sleep deprived. So, it might take a bit more time to get information into our heads than on a regular day anyway. **[Patient24_L3]**

		R: Yeah. And that took several hours it took for me to…and, you know, I'm not one for shouting unless I really have to but I really blew my top, to be honest with you. It got to a point where we'd asked several times, (name) had been so patient. He was, at this point, throwing up, he was in absolute agony. All the progress that he'd made in the ITU had gone. He'd, at one point, asked to switch the machines off and said I don't want to be here, I can't do it, he was in that much agony. And as a mother, to hear your son say that, as you can imagine, that was the point that I blew my top and I demanded to see the consultant. And finally, after hours and hours and hours of waiting, they finally prescribed him…the first thing was paracetamol but then got him something stronger, which was they put him on…I believe was oxycodone which, it was the button that was…and then that started making him sick. **[Family25_L3]**<br><br>I: When you talked about having to go to the consultant and be fairly forceful, as it were, you know, what was that like, what did you feel about having to do that?<br>R: To be fair, it was a mixed emotion, if I'm being honest. You know, inside I'm broken because my son is in agony and there is nothing that I can do. I'm angry because I know there's people that can do something. I know that they're stretched and I understand and totally appreciate that, however, I just never felt that they were on our side. So it always felt like a bit of a fight. I had a consultant's words to me, when I…because to me, they weren't checking (name), they weren't…I'm very clued up on healthcare, so I work in the healthcare industry. So, you know, they'd done no pressure care with (name), they'd not come in to check for anything. His feet were overhanging off the bottom of the bed, they'd not offered to take him up the bed. There were so many things going on that it just built up over this time period of (name) being in the state that he was in. I said to the consultant, it's institutional abuse, which it was. And he said, I completely agree. And that to me was just like the icing on the cake because then he said, however, we are short staffed. But, you know, for me, how is that the patient's fault? Not just my son, I don't doubt other patients are probably in the same boat, however, you know, it shouldn't be that way for anybody. So it was such a mixed emotion, to be honest. It's quite heartbreaking as a mother to watch your son in that much pain to the point that you feel like you have to blow your top just to be felt like you've been heard. **[Family25_L3]**<br><br>I: And that was around some of the antibiotics as well. What was that like, you know, having to do that?<br>R: To be fair, you almost expect people to tell you, you know. And I appreciate that the patient might not be asking the questions but, you know, watching one of your family members having all these things pumped into them, it can be quite daunting. And not having the understanding of what they are being administered and why they're being administered it, you…well, for me personally, I felt almost like I was a bit blind to it all, like I didn't fully understand what he was going through, you know, it made me feel quite…emotional is probably the best word because you're already in a very scary situation. So just having the information helps you find the answers, you know, in there, you can, sort of, piece together what is going on. Does (name) have an infection? No, he doesn't but we're doing this as prevention. Well, that's great, at least they don't have to worry about an infection. **[Family25_L3]**<br><br>I: Yeah. How do you think family members can be best involved in this? Because I've spoken to quite a few people now doing this project and we're beginning to build up an idea of what might that role, if you like, be. But what do you think, you know, in terms of when you've had someone being in critical care and then transferred down the ward, how do you think family members can be best involved?

		R: I think first and foremost, they need to be reminded that it's not going to be like it is in ICU. It was a massive…I want to say downgrade but I don't mean it to sound like it, if that makes sense. But you go from this immaculate care, one-on-one, everybody's got so much time and patience and willing to give you them answers. To, you get transferred down to a ward, half of them don't even seem to know anything about you, don't know what you've been through as a family, as the patient. And then you're re-living every moment again and you're already exhausted emotionally and physically. I think that would make a massive difference is having the staff, you know, understanding the patient before they get there, rather than when they are there. I also think, you know, having them explain that it's not going to be that one-to-one care because I think that quality of one-to-one care, that it's almost like being in a royal palace, they're going in and staying in, I don't know, some cheap hotel down the road, you know, it's never going to be the same level, is it, of quality of care? I'm not saying it isn't but that's how it feels. And the process in between that as well, like, they're not…they don't educate you on the difference that it's going to make in terms of their physical care and things like that as well. **[Family25_L3]**<br><br>I: … for both the patient and for relatives, I think. Apart from the pain medicine, were there any other…or have there been any other problems with his medicines whilst he's been there or particularly around that transfer?<br>R: No, not really. As for the rest of them, they've been fine. It was more his…to be fair, they stopped everything on the day, he'd not got his fluids that day, he'd not got his pain relief, his anti-sickness. He'd literally got nothing. Like I say, he'd not even got paracetamol, nothing. The room that he went to has got a massive heat pipe radiating heat off it, it's like a little boxroom type thing. It's still on a bay but his bay has got this heat pipe inside it just generating heat. And he was overheating, and he already had a temperature. And then they put him in this bay and then didn't give him any medicine at all. And it's things like that, you just think really, you know? And he can't drink, you're not saying he's not allowed to drink but yet you've not given him no fluids, that's how long without fluids? Then the pressure kicks in. Then seeing your son deteriorate on top of major surgery and the life-threatening experience that you've just gone through and thought you'd come out the other side, it's so very overwhelming. **[Family25_L3]**<br><br>I: Right, I'm with you.<br>R: And that makes things more difficult for patients like myself.<br>I: Yeah, absolutely.<br>R: Because we're often sat in pain, and there's nothing, really, we can do about it. And it's very frustrating, and it makes the quality of life more unbearable.<br>I: Yeah, yeah.<br>R: Like, I'd really like to see pain relief for renal patients being developed, so that we have something stronger to take, when we're in intensive care, and we're in pain, you know, [inaudible 0:01:52], et cetera. **[Patient27_L3]**<br><br>I: Right. And how did you feel about that, then?<br>R: Well, to be quite honest, at the moment, it's just got me more confused. […] And more upset, and I'm just trying to just hold onto the fact, you're on your proper medication now, you're building up your proper allowance, so you might not feel as down, and disheartened, and depressed. […] That's the only way I can deal with it, at the moment […] R: I can't get angry; I can't scream and shout about it. […] I just need it to be out of the way, I just don't want it to be an additional problem. **[Patient28_L3]**<br><br>I: Right, okay. And does that mean you've had to take more on in talking to the doctors and nurses then?<br>R: Yes, I've had to take more on what with (name) being on his own, a lot more own. Because me and my wife are finding it hard because we've got my aunty to look after who's sick and bedridden and we're up here, up at hospital and my son is living in assisted accommodation so we have to see to him as well [inaudible 15:09].<br>**[Family30_L3]**

Theme 2	**Initial Theme - Knowledge and expectations**<br><br>Final Theme - Pre-existing knowledge and capability	
	**Sub Theme**	**Quotations**
	**Expectations (or experience of) of likelihood that HCPs will withhold information, disagreements, conflicts or other negative engagement**	It wasn't until I… I was given my drug regime. You know, I was given, like, a couple of paracetamol because I had trouble with my temperature there, because I had a bit of a temperature, it was quite a serious operation, and I just said, well, why aren't I having this? She said, because… No, I said, the only thing that were in the letter – I said, it's in my […], if you pass me the bag, I'll show you the letter. It said, do not take metformin on the day of the operation. Afterwards, I said, I'm back on my normal meds. […] And once I realised I wasn't taking it […] I was a bit stubborn, I stood my ground, like. I said, you're not doing that until this is sorted. Within 12 twelve hours it was sorted. **[Patient01_L2]**<br><br>R: …I just, you get frustrated, that's it. That's the only thing you do, you just get frustrated.<br>I: So, why do you get frustrated? What are you frustrated about?<br>R: I don't know. It's still about the medication. And it's, nothing's getting done, even though you're in hospital and you want to sort it out, but you can't actually sort it out until you get out of hospital. And you don't know how long you're going to be in hospital for. And then, it's just one of those things and when anyone comes up to you, the doctor might just turn round and say, sorry, you can't stop that. You've talked to the doctor that gave you them in the first place […] if that makes any sense? So, if you, so, that's why, I'm just waiting now, keeping quite 'til I leave hospital. **[Patient07_L2]**<br><br>I: In terms of the way people would talk to you then, how do you feel about how you were…how these things were discussed with you and how the conversations went?<br>R: In regards to in-depth information is what I wanted, pretty poorly up until the point of speaking to the doctor in respiratory, on the respiratory ward. I mean the guys in critical care were fairly vague with what kind of medicine they were giving. They didn't give any names really.<br>I: Right, that's interesting. Why do you think that was? Did you ask questions further or…<br>R: Oh yeah, always ask questions, yeah. I don't know, whether they didn't know the names of them or not, I don't know. I couldn't tell you. […] Or they just didn't want to divulge the information. **[Family20_L2]**<br><br>I: And do you think you're getting enough information then as a consequence?<br>R: You never get the full picture with doctors unfortunately. They can be very vague. But you have to ask the right questions otherwise they don't answer properly, which I have found. And there has to be more than one of you because obviously two people have got different points of view, different questions, different sides of questions to ask. So it's easier when there's a couple of you asking questions<br>.I: So you think that's a…yeah, that reluctance is because they think you won't understand or because they are being guarded?<br>R: Possibly. I definitely think a bit more transparency when it comes to these things is needed. I would certainly say that. **[Family20_L2]**<br><br>I: Right. And…so you know that's happened, but they didn't discuss with you why…<br>R: No.I: …why they were swapping that over to something else at all.<br>R: No, no idea why.<br>I: Right, okay. And so you haven't…?<br>R: To be fair, they don't talk to you at all unless they have to, do they? **[Patient22_L3]**

		I: Who's been in hospital. Thinking about them and thinking about after they've left…the medicines they were given after they left critical care. Can you just, you know, tell me about the sort of conversations you've had with any of the hospital staff about the medicines and stuff like that?<br>R: I mean, it's been a bit tricky, they've not actually been that great at communicating unless we've been pushing them for information, to be honest. So they are very good at coming in and saying to (name), we have, for example, your blood thinner to give you, we're just going to do it in your right arm, as such. Not necessarily, (name), can we do it in your right arm? They will tell us what it is and things like that. However, most of the time, they don't really explain what it's for. […] Yeah, so they might say, I don't, (name), we've got your antibiotics but they don't specifically say these…or haven't specifically said that the antibiotics are for general infection, protection against infection, whether he is currently fighting an infection, you know, it's been a little bit like that. **[Family25_L3]**<br><br>R: And that took several hours it took for me to…and, you know, I'm not one for shouting unless I really have to but I really blew my top, to be honest with you. It got to a point where we'd asked several times, (name) had been so patient. He was, at this point, throwing up, he was in absolute agony. All the progress that he'd made in the ITU had gone. He'd, at one point, asked to switch the machines off and said I don't want to be here, I can't do it, he was in that much agony. And as a mother, to hear your son say that, as you can imagine, that was the point that I blew my top and I demanded to see the consultant. And finally, after hours and hours and hours of waiting, they finally prescribed him…the first thing was paracetamol but then got him something stronger, which was they put him on…I believe was oxycodone which, it was the button that was…and then that started making him sick. **[Family25_L3]**
	**Expectations as to what is normal or usual**	Well you're definitely not in the early stages are you when you're in the HDU or whatever it is, but as for the transfer between the epidural and everything else, I felt really hundred percent that I was given the option basically, you know, there was this thing called Tramadol and I…[inaudible 0.18.55] but I think I'd rather avoid it if I possibly could, you know, that [inaudible 0.18.59]. I've still come home with some oramorph that's, you know, I think that's par for the course isn't it, that's a standard thing. I mean, I haven't used it, I don't suppose I will use it. **[Patient12_L2]**<br><br>R: Well, as far as I know, knowing the cholesterol situation won't change or my potential glaucoma, so I would anticipate carrying on, but I assume…I haven't been told anything yet, but I assume there's some sort of clinical review in a month or so, I can put it across to him then.<br>I: Right, okay. So, has that been…?<br>R: That hasn't been arranged, no.<br>I: No, but have you been told that that's going to happen?<br>R: No. **[Patient14_L3]**<br><br>R: Because the first couple of days, it were overwhelming. […] Because I'd never been in critical care, to see what was happening.<br>I: No, no, and it's a very different environment, isn't it?<br>R: Yeah, it's traumatic when you first see it. **[Family17_L2]**<br><br>R: I don't know, because I've never had much dealings with hospitals, so I wouldn't know.<br>I: Yeah. Yeah, do you think this is sort of how things work normally, then, or do you think this might be what usually happens, as it were?<br>R: I just accept, that is the way they work […] You know, because like I said, we've never had no dealings with hospitals. **[Family17_L2]**<br><br>I: And that was around some of the antibiotics as well. What was that like, you know, having to do that?

		R: To be fair, you almost expect people to tell you, you know. And I appreciate that the patient might not be asking the questions but, you know, watching one of your family members having all these things pumped into them, it can be quite daunting. And not having the understanding of what they are being administered and why they're being administered it, you…well, for me personally, I felt almost like I was a bit blind to it all, like I didn't fully understand what he was going through, you know, it made me feel quite…emotional is probably the best word because you're already in a very scary situation. So just having the information helps you find the answers, you know, in there, you can, sort of, piece together what is going on. Does (name) have an infection? No, he doesn't but we're doing this as prevention. Well, that's great, at least they don't have to worry about an infection. **[Family25_L3]**<br><br>I: Yeah. You've actually used a phrase there which is I find it really interesting about 'depth of explanation'. You know, is that what would be useful to have that, sort of, further…?R: I think so. I think knowledge is power. I think if you understand what you're facing and what the plan is, you can better prepare for it, you can better understand what to expect for both negative and positive. You don't panic if something bad, you know, goes, if you're prepared for the potential of that. Instead, you get slapped with something negative that happens and actually you think you've just jumped back 10 steps but really in reality, it's quite normal. But you don't know that it's quite normal until it happens, if that makes sense.<br>**[Family25_L3]**
	**Expectations of positive engagement around medicines and medication plan**	I: And in terms of medicines, do you know if there's a plan for your medicines when you're discharged?<br>R: No, not up to yet, I haven't been explained as to whether they'll be [inaudible 12:58] medications or not yet.<br>I: Right, do you think that's something that you would like to happen, to have that conversation?<br>R: Yes, I mean, in the past that conversation's normally happened on discharge.<br>I: Right, okay, so on occasions when you've been in hospital before, you've had a conversation with…<br>R: They've decided it's safer or better for you to move you from this medication to that medication. **[Patient10_L2]**<br><br>Well, I mean, I've got a complete schedule for everything I have to take and where I have to take it. And some of the tablets say reintroduce full Atenolol on 6 March or whatever it is and, you know, start half the dose of Lisinopril on 7 March, or whatever it is, you know, it's all down. There is a schedule, but I do need…I've got to have my staples taken out today, so I try to get round the 08:00 phone call thing and say, well can I please come and talk to someone a-sap about what's going on, because although the Furosemide is working, I've still got plenty of fluid on board and you can probably tell from the way I'm breathing, it's not particularly clever, but what I don't want to happen is the same things happened last time, I do not want to be taken in an ambulance ever again in a hurry. So, I need to manage that a bit better I think, and just make sure it is a session with the GP as opposed to the paramedics. **[Patient12_L2]**<br><br>I: Right, okay. And a couple of more questions really. One of those is around a plan. Do you know what the plan is for your mum's medicines going forward from now?<br>R: Not really, not yet, because when she had the surgery, they weren't exactly sure what they were looking at. Because they'd found a mass in her stomach and they said it could be a cluster of ulcers, because she had an ulcer in her stomach about six weeks I think before this. And so they were either looking at that or they were looking at a tumour or cancer, however you want to say it. And that is what they found. So when they did the surgery, they took biopsies and those results have not come back yet. So I think it all depends on them results from them biopsies as to what kind of treatment she'll have in the future.<br>I: So at the moment things are a bit up in the air and you're just waiting for that, those test results?<br>R: Yeah, it were the surgeon we spoke to and he said they could take about two weeks to come back, and it's been a week yesterday since the surgery, so we're hopefully waiting to get them next week. I think also, with it being what they found with it being cancer, they said she will at some point be referred to an oncologist. And then that will get decided exactly what they're dealing with, I suppose. **[Family15_L3]**

		So far, it seems to have worked. So that was a new thing that was sent out and I did know what it was but she did explain it to me. And she got some Oramorph which she has taken in the past, but not recently. Yeah, they gave me a complete list of all the medication she was on. They went through it I thought quite thoroughly. I know [they have to know everything 30:31] before she started but she did go through it properly and carefully and, yes, on discharge. Yes, I thought it was very good. **[Family16_L2]**<br><br>I: Firstly, do people know you work in the NHS and does it change the way they talk to you?<br>R: I think it does a little because I think they get this sort of like mutual ground with you, but they'd spend a lot more time explaining stuff to me I feel than my partner's mum. I think that's mainly because she didn't understand, if that makes sense. I don't think it's a reflection on them trying, I think it's more the recipient. So when she didn't really understand, it's a bit flogging a dead horse sort of thing, isn't it. Like fentanyl, she didn't know what it was, she didn't know what the purpose was, and it didn't matter how many times they told her this is for that, she never quite understood, if that makes sense.[…] So I think it's more maybe if you can understand the information they probably will spend more time explaining it rather than someone that doesn't understand. **[Family18_L3]**<br><br>Yeah, I mean, I think probably past experience with, I've not had a bad experience with the NHS, I know some people have. They looked after my mum really well when she was very ill, and I guess it's just the fact that they're the professionals, they should know their job, and what's best for that patient and that patient's needs. […] Yeah, you know, I can't come along and say, I think she needs this, that and the other when I don't really know exactly what's wrong with her.<br>**[Family21_L3]**<br><br>Following that, things improved dramatically. So suddenly, everything changed. (name) started getting his medication. They've been quite prompt with that since, so they're not dawdling. If (name) asks for some more pain relief, you know, and he's due his pain relief, then yes, they'll administer it. If he's not, they're, you know, speaking with the consultant quite quickly and promptly to ensure that (name) does get something that he can have. So yeah, since then things have turned around quite dramatically. **[Family25_L3]**
	**Patient (or F&F) confidence in their knowledge**	R: It wasn't until I… I was given my drug regime. You know, I was given, like, a couple of paracetamol because I had trouble with my temperature there, because I had a bit of a temperature, it was quite a serious operation, and I just said, well, why aren't I having this? She said, because… No, I said, the only thing that were in the letter – I said, it's in my [inaudible 00:02:54], if you pass me the bag, I'll show you the letter. It said, do not take metformin on the day of the operation. Afterwards, I said, I'm back on my normal meds.<br>I: Right, yes, right<br>R: And once I realised I wasn't taking it [inaudible 00:03:08]. I was a bit stubborn, I stood my ground, like. I said, you're not doing that until this is sorted. Within 12 twelve hours it was sorted. **[Patient01_L2]**<br><br>I like to know what medicine my dad's on, and I do read up a lot on it and [voices overlap 09:07], why he's taking it, what he's not taking it or…and also because I have a science background, I did a BSc in biomedical sciences, so I'm quite…I like to know what's going on [voices overlap 09:18]. So, I think I'm more interested, so everyone left it on me, you're our doctor in the house, you sort it, so it's like one of those things.<br>**[Family03_L2]**<br><br>I: Right, and thinking about…I mean, were you on any medication before you went into hospital?<br>R: What for pain control?<br>I: Oh, just anything?<br>R: Oh blimey, yes about six or seven, now I'm on about 14 at the moment, I think and counting. They seem to…I may well have replaced oesophageal cancer with some kind of heart failure, I'm not sure the way things are going at the moment, but yes, I was on about seven different things.

		I: Right, okay. And do you know if there's been any changes around those medicines made while you were in hospital?<br>R: Yes, I do, and I know exactly what they are **[Patient12_L2]**<br><br>I: No, right. How do you think patients can be best involved in their decisions about their medicines?<br>R: I think it's quite tricky these days isn't it, because a lot of people have got some knowledge of medicines and I'm very much the exception to the rule, I think I've got quite a lot of knowledge of medicines, and a little knowledge of them is probably a bad thing, stuff looked up on the internet or what have you. I don't know, it's just everyone's different, aren't they? I don't think there's a sort of general rule that you could apply. I suppose what we've got now which is let's go and see this person and see, you know, whether they are able to engage in the decision themselves or whether they are quite happy to let the doctors know best, I don't really know.<br>I: Yes, well, it's almost you say, yes, it's almost like an individual, personal, case by case basis, isn't it.<br>R: Yes, I mean I think most people when they go into hospital they feel as though, like, you know, do whatever they want, these guys know best, you know.<br>I: Yes.<br>R: But as and when it became a decision for me, if there was certainly any decisions being made for me, I was certainly allowed to make it myself **[Patient12_L2]**<br><br>No. I don't know whether she was or not. Unfortunately, she…some years ago…three years ago, had an operation on her bowels and prior to that she'd been on very high doses of fentanyl, so she may well have got some tolerance to opioids as a result of that. Since that operation, the fentanyl was gradually reduced and she hasn't taken it for a couple of years, you know, [for 05:01] getting off it. But I think it made her a bit less sensitive to opioids and therefore they had to increase her medication, pain relief, to a point where really she has had…had…she's okay now, had little memory and was really at one point a little bit delusional and was hallucinating. So even if they'd told her, I doubt she would have remembered it. Her short term memory was hopeless. I mean, since then the medication, painkilling medication/analgesia has been reduced and she's now on relatively small doses of things and her mental abilities have come back. **[Family16_L2]**<br><br>But when she was in the hospital with so many other…I mean, she had six infusions…she got seven infusions going in, a nasal tube, a drain…a blood drain, a catheter, urine bag, electrodes all over her. I mean, you couldn't move…she couldn't move without setting something off. By the time she got to the ward, of course we were down to just two infusions. And then…so those infusions just went up as normal and then from my experience they gave her all her tablets, possibly at a slightly different time, but that didn't matter. Nothing was missed as far as I can tell. And nothing was doubled up or forgotten. **[Family16_L2]**<br><br>R: So basically what would happen, my partner would get started on different variations of medication. He had quite a nasty infection and the cultures had not been grown from the lab. They weren't too sure actually what the infection was. So when I get there in the morning, there'd just be different antibiotics, names and different bottles and stuff. So I was quite inquisitive because I do work in the NHS myself.I<br>: Oh right, okay. That possibly makes a difference.<br>R: I'm not clinical but I do work in a clinical environment so I do have a bit of an understanding, so it made it a lot easier for me. But I think like my partner's mum, for instance, who doesn't have any medical terminology or anything, she found it really hard to understand what all the medicines were and what they were for. But the staff in the intensive care unit were very, very helpful and tried to explain, like this is for blood pressure, this is for this. So they were really good in explaining stuff. **[Family18_L3]**<br><br>I: Right, okay, good. Yeah. Did you prompt anyone for any details or was that discussion initiated by them?

		R: I don't know what other people's experiences are like, but, obviously like I said, because I do know bits and bobs as soon as I got there in the morning and asked what his blood pressure medication was on, how much clonidine he was on, I'd ask all different questions about the medication because I remembered them because I used to see him every day. So if there was a new one I'd be like oh, what's that for, what's this.<br>I: So you're not clinical but you do work in the NHS.<br>R: Yeah. I'm a practice manager at a surgery. I don't work in a hospital environment […] But I've got some sort of clinical knowledge […] being in that environment. […] I mean, and reading letters and I do coding and all sorts. I've got a bit of a basic understanding, like a blood pressure reading. I knew when I took him to the doctors and it was 82 over 46, that that was a problem. Whereas obviously people who don't work in that environment wouldn't know that was an issue. **[Family18_L3]**<br><br>I think, generally, I think they should ask, at least, because I know that I keep a prescription leaflet of my mum's, or my dad's, at home, just constantly, just in case. I think, I don't, obviously, I don't know what people, like, whenever they're going into a scenario, like I obviously know they end up having to read up more about the patient. But I feel that, if they seek out the family, in order to, I mean, just make sure that there was certain medications that they were aware they were on beforehand. Because I know, one of the major ones that I don't think I've mentioned at the moment, is she's on citalopram.<br>**[Family29_L3]**
	**Patient ability to engage - e.g. conditions, hearing, memory, cognitive ability, prior knowledge, health literacy, education.**	I: No, and so if you've, have you said, oh, I don't, any time, have you said, oh, I don't want to be on that medicine or I don't want to do that or can we do this? Have you pushed anything like that?<br><br>R: Because I don't know nothing about it or about the medicines, I don't want to say anything. Does that make sense, until I know what the medicine is. That's what I'm planning to do. That's what me and my wife are planning to do. When I leave the hospital, I'm going to find out what medicines I'm on, what they're for. Because I'm on about 28 some form of different tablets. At least 28 and that's quite a lot for me. And it depresses me. It depresses me. So, that's what we're going to go through. We're going to go through each one, even if the doctor likes it or doesn't like it. I, we want to just going to see what's happening. **[Patient07_L2]**<br><br>Interestingly though you're talking about medicine, she said, my mum did say to me today, she said, I don't know what medicine they've given me this morning, (name), she said, and she talked about the tablets so she's not been told at the moment what medicines she's having and what it's doing. I mean, I'm sure if they asked but they're not actively telling her and I guess, I could have at that point said to her, Mum, would you like me to ask? One thing that I do notice is that when they do do their rounds, she likes me to be there and fortunately I have been there because they do their rounds in the morning. This is only what happens on this ward and they tend to be a team of people that come. So you've got your main consultant and you've got presumably your trainee doctors and so they all kind of like arrive on troupe because they're kind of like learning and she almost panics and says to me, (name) you will stay in the room. I think it's just…my mum's always been very fiercely independent, she's a retired teacher and she's very articulate and things like that, I think she just finds it a little bit overwhelming now and wants me to be there to listen to possibly ask the questions, you know. Yes, I don't know. **[Family11_L3]**<br><br>I: Yes, because around those decision…in that process then, as you say, this morning she'd been saying, oh, they've changed some medicines, I don't really know what they're doing with those.<br>R: Yes, she did, yes, and I wouldn't have thought of it had you not said about medicines but she definitely said to me this morning, I don't know what the medicines are that they're giving me and what they're for. **[Family11_L3]**<br><br>I: And as you said, your husband, do you know whether they've talked to him? But of course, you said he wasn't particularly able to take the information in.

		R: Well, he's hard of hearing, and at the moment, he's not wearing his hearing aids […] So, sometimes they'll say to him, and he'll not understand. And he'll try and tell me something, and then I panic, so when I get there, I ask them, and they'll say, no, no, we said this. Because, like, at one point, he thought he'd got sepsis, and he hadn't […] It was streptococcal, or something like that, Strep A, it was that, but he thought he'd got sepsis […] Because his hearing, he's not wearing his hearing aids at the moment.<br>I: Right, yeah. Yeah, so he's finding it difficult to have conversations with the medical staff, as well, then.<br>R: Yeah, at the moment. He doesn't quite understand what they're telling him. **[Family17_L2]**<br><br>I: Yes. Actually, this happened with somebody else I interviewed who also worked clinically in the NHS or had done previously, and I asked them the same sort of question. Firstly, do people know you work in the NHS and does it change the way they talk to you?<br>R: I think it does a little because I think they get this sort of like mutual ground with you, but they'd spend a lot more time explaining stuff to me I feel than my partner's mum. I think that's mainly because she didn't understand, if that makes sense. I don't think it's a reflection on them trying, I think it's more the recipient. So when she didn't really understand, it's a bit flogging a dead horse sort of thing, isn't it. Like fentanyl, she didn't know what it was, she didn't know what the purpose was, and it didn't matter how many times they told her this is for that, she never quite understood, if that makes sense.<br>I: Yeah.<br>R: So I think it's more maybe if you can understand the information they probably will spend more time explaining it rather than someone that doesn't understand. **[Family18_L3]**<br><br>I: But do you know who made the changes? What was the…<br>R: I don't. I don't know. I couldn't tell you […] I'm assuming it was a consultant or the doctor themselves.<br>I: But no one came to you and said right, we're going to change the medicines to this because of that? Were there any of those conversations around that?<br>R: No. There possibly were the conversations with my father but his memory's not very good. So he's… I mean you ask him questions and he gets things wrong and you then speak to a doctor, it's like no, no, this is what we've said […] Which I always do anyway. I always ask the doctor after something's been said. **[Family20_L2]**<br><br>R: I mean I raised, when she was asleep when I went the other day, I did raise to the staff about her memory, that's my main concern is how her memory's been affected. So there's not a lot with regards to her medication that I'm concerned about, so I've kind of left that to her to deal with. But my main concern is her memory, so I've been talking to people about that, more than anything.<br>I: Right, because you said she can't remember, is it…?<br>R: So she's literally, she has a 24 hour window, so she can remember what's gone off in the past, and she knows who everybody is, and she knows of things coming up in the future, but she doesn't know anything we talked about yesterday. […] She'll have forgotten that, so it's like a 24 hour window. She didn't know what year we was in, or what day of the week it was. […] So I've took her a little calendar in, that she looks at and turns over every day to try and get that ingrained back in her head. **[Family21_L3]**<br><br>R: I know, yeah. And back to the ward on Saturday, the third, so he was in critical care for a week, gradually getting better. And then on his transfer to the ward, he was totally with it, you know, he was back with us. So I asked him yesterday, because I knew I was going to do this interview today, about his medication and everything, and he knew everything, all the medication that he was getting, from the nurses. So he was able…it wasn't a conversation I needed to have with the nurses, if you know what I mean. **[Family23_L2]**

	**Prior knowledge about previously prescribed medicines**	The most recent occasion. Yes, when I were on the ward, because they gave me painkillers, they gave me tramadol, but they told me to keep that… They only gave me enough to sustain the…you know, the…no pain [voices overlap 00:00:47] that was all. The only problem I did have, and I had a bit of a fall out over it, when I went in for my pre-med, I had to take in a list of my medicines. Well, I take metformin for type 2 diabetes, I take a statin I've just started taking on recommendation, not because I need it but because of my age, I'm 69. It's to do with, like, coronary heart attacks, all of this lot. And the other one is an anti-reflux tablet which I take.Now, when I went in, I got a letter from the people. I took it into hospital with me. Now, all it had on there was do not take the metformin, only one that day. When I was in there, they were coming out and taking my sugar, they wouldn't give me metformin. And I asked why. And so they said, well, the doctor said so. I said, well, the doctor's not told me that, and I said, I want to know why. They said, we can't tell you. Well, I said, I'll be quite honest with you, the way I feel at the present time is, it's pointless me having my sugar being taken three times a day when whatever reading it is… Once I was 12.9. I says, it's quite pointless me having this procedure… Anyway, they did come back, talked to me, and they put me on it. **[Patient01_L2]**<br><br>R: I know the occupational therapist rang yesterday, she sorted out a bed for my dad and a toilet rail, so that came this morning, and they're just waiting for a grab rail for the door, so they're helping him get comfortable at home, and it's just the medication that needs to get sorted.[…] Because, obviously, with his cholesterol tablet being stopped whilst he's also on antibiotics again, so…and his Allopurinol tablet gone down, so I think now he'll be…I'm going to have to write down exactly when to take them all or not to take [inaudible 06:57], because I do my dad's…I manage my dad's care and everything, so I write it down on his boxes, two in the morning and two at night, it's more easy for him to see and everything. But now that he's really weak, I will definitely be going down and about the time he takes his medication, I'll be going down and giving it him and everything. **[Family03_L2]**<br><br>I: So, we've started that recording. So, brilliant. So, just thinking about your medicines. If, you know, you were given to take, after you'd left critical care, after you'd left ICU, can you tell me about the sort of conversations that you had with people about those medicines?<br>R: Well, there was a bit, for instance, from my diabetes medicines and I was on [inaudible 00:31], I've always been on some sort of certain units of insulin. So, for instance, I've been on 60 units, every time I eat. If that makes any sense but for some reason when I was in the hospital, they only gave me ten units and I didn't know why was that at first. But when I started realising, it just, well, hold on a minute, at home, I eat quite a lot of stuff extra. Does that make any sense?<br>I: Yeah, yeah.<br>R: But at the time, I didn't realise it, at first, because I thought there was, there's something wrong here and then when they tried to explain it to me, it didn't register at first. Then it did afterwards, if that makes any sense? Is that what you're on about? **[Patient07_L2]**<br><br>I: Right, and thinking about…I mean, were you on any medication before you went into hospital?<br>R: What for pain control?<br>I: Oh, just anything?<br>R: Oh blimey, yes about six or seven, now I'm on about 14 at the moment, I think and counting. They seem to…I may well have replaced oesophageal cancer with some kind of heart failure, I'm not sure the way things are going at the moment, but yes, I was on about seven different things.<br>I: Right, okay. And do you know if there's been any changes around those medicines made while you were in hospital?<br>R: Yes, I do, and I know exactly what they are. **[Patient12_L2]**

		R: She has…she's taking a lot less medication than the medication that she went in with, but she was having to have 24 loperamide a day, which…and she doesn't take any now. The thyroxine is 75 micrograms, they've kept her on that. Amlodipine one milligram, they kept her on that. Multivitamin and minerals, not sure about that, whether they…she said she hadn't had a blue tablet, so maybe they didn't give her that but it's…as I say, it's only multivitamins, not essential. Her calcium, they've kept her on that. Creon, she was given that. Codeine phosphate, they did take that away because of all the other medication she was on, but then that was reintroduced as a painkiller. She was actually taking it, not as a painkiller, to reduce the diarrhoea which is the whole purpose of this operation. So that was taken away at one point because it was being replaced by other things. And then it was reintroduced on the ward to help with the pain relief. The ondansetron, they took away. And I asked her about it and she said she had no nausea so there was no point in taking it. The [letrozole 08:27] tablet, she thought one day they might have missed that in the HDU unit, but she always had it in the…on the ward. So in terms of transfer, everything was perfect. She…having had her operation, they've stopped any paracetamol. That's it really. Yeah. That's everything. **[Family16_L2]**<br><br>R: Well I used to be a pharmacist […] so I could actually recognise tablets just by looking at them, mostly, but not always of course. I noticed the ondansetron was missing. There was no conversation with me about why that was taken away. But I could see the logic for taking it away. I don't know whether they'd had a discussion with (name of patient/participant's spouse) about it, possible, possibly not, I don't know. But anyway, we hadn't…now we're home, we haven't reintroduced it. We have some here, but we haven't reintroduced it because she was getting a bit of nausea from the codeine phosphate…well that's what I assumed it was anyway. And the ondansetron just completely took that away. So we kept on with it. But I think now because she's taking the codeine regularly for a longer time, her body's got used to it, and possibly the ondansetron isn't needed anymore. So we shall continue exactly as she left the hospital, with no ondansetron and…in fact, she had reduced it herself from three a day to two a day, with no discussion from me, prior to going in. And so…yes. But that is there, we've got it should that recur. But it hasn't done so far. I mean, it's early days yet and she'll [voices overlap 15:01]. **[Family16_L2]**<br><br>I: And so was he then, whilst you were waiting in this year before, was he on medicines before he went into hospital?<br>R: He should've been on medicines before he went into hospital. The only one he took regularly was Omeprazole for his really bad indigestion. When the paramedics took him to the ambulance on Friday night, I had a scour around the room and I found several boxes, including… There was Cocodamol, there was folic acid, there was vitamin D3, which all of them were unopened. Which I'm pretty sure he's been prescribed for a reason.<br>I: So he was prescribed but not actually taking them.<br>R: No. That's correct, yeah, he was prescribed but not taking them.<br>I: But he was taking his Omeprazole?<br>R: That's if he had any left.<br>I: So he might not be…<br>R: With the way… My dad's… He's an alcoholic. So he didn't eat a great deal, drank a lot. So obviously one of the effects of that can be chronic indigestion, can't it? […] So yeah, that's why he'll have burnt through the Omeprazole. **[Family20_L2]**<br><br>I: Do you know of any other medicines that were changed or anything else that was changed about that since you came in?<br>R: I'm only on two medications before I came in, one was the blood pressure tablet called Amlodipine, that's been changed to Ramipril, and they've stopped my antidepressant, they were only the two tablets that I took for my arrest.<br>**[Patient22_L3]**

		R: Again, when it comes to this, it's very much like, my mum has very much had a similar scenario, two and a half years ago. So, the sequence of events has very much mirrored, sadly, a couple of years ago. And I'm not sure whether they have a defined plan, but it's more, me and mum when we're talking, and it's more of the assumption, this is probably some of the medication she'll be on. Because I know that she ended up having, initially, when she got first stoma, because this scenario came about from her having it reversed, as well as (a hernia?) ended up like reduction and removal.<br>I: Right, okay, yeah.<br>R: So, she knows that she will have to be on loperamide again. She knows that she will have to, at least, have pain relief. But we haven't really discussed, like, what's going forward. It's more like, based off the initial assumptions of, this is what happened last time, this is, kind of, the standard of what's going to happen, or from the assumptions we can make, we're just seeing that, oh yes, she's on loperamide again, she's taking her inhalers again. And it's more, we're making just assumptions based on the information we've already had.<br>I: Yeah, rather than any new, someone coming down and saying, right, this is where we're going to go. And it's fairly early days, presumably, as well, because she's…<br>R: Yeah.<br>I: …it's three or four days since she left critical care, is it?<br>R: Yes, it is, yeah, it's not been that long yet. So, yeah, it's very much like, even though there hasn't really been a plan, or a structured like scenario, like what are we going to be going into. It's more, I think they're seeing how she gets on, that's probably the best way to put it.<br>**[Family29_L3]**

**Theme 3**	**Initial Theme - Beliefs about persons roles.**<br><br>**Final Theme - Beliefs about persons roles and expectations.**	
	**Sub Theme**	**Quotations**
	**Beliefs about patient (or family) role**	I: That's fine, that's good. So, when he was talking to you, were you encouraged to ask any questions about that, about your medicines as well, or was it just…?<br>R: I don't need encouragement. That metformin, as soon as I realised it, I were in [inaudible 00:11:11] straightaway. And if I had any doubts whatsoever what I was being given without explanation, I'd have pulled them. I'm not shy in coming forward, not by a long way […] I think if I'd have turned round and said I don't want the tramadol, you know, the effects that it has, like, and they are saying it's the…advising me it's the best they can do, but I certainly feel if I'd have said I don't want it, they wouldn't have, like, tried to railroad me, they'd have given me something else.<br>**[Patient01_L2]**<br><br>I: Yeah, good. And do you think…I mean thinking generally then how do you think patients can be best helped to be involved in their medicines?<br>R: I think that's a difficult one because some patients want to be and some don't. And maybe I thought that the…prior to it happening that I wouldn't have wanted to be involved but as it turned out I did want to be involved. So, I did ask a question. But if you'd have asked me beforehand, I'd have probably said, no, I'm not really bothered […] But as it turned out in the end it was like, well why are you giving me that, or, what are you giving me that for?<br>I: What do you think changed your mind now? What do you think?<br>R: I don't know, I can't say. Maybe I would have always said that, I just thought I wouldn't. And when it turned out in the end I did want to know. Whereas perhaps I'd thought, oh no, I'd rather not know, thanks, as I said, but as it…when it turned out in reality it was a case of, well actually, yes, I do want to know what you're doing. **[Patient04_L2]**

		I: Do you think patients should be involved, and it what ways can they be involved in those decisions around medicines?<br>R: I'm not sure that they should be. […] You know, I'm an intelligent guy I, you know, but I'm no medical person, I'm 71, you know, I can go to Boots and buy paracetamol, Ibuprofen, can go to the Boots Pharmacy and say, look, I'm feeling a bit like this, what would you suggest? But anything beyond that I have no knowledge whatsoever, and yet I'm sat there in my bed in front of a consultant who's got 30 years' experience, you know, why shouldn't he decide for me? **[Patient08_L2]**<br><br>I: Firstly, do people know you work in the NHS and does it change the way they talk to you?<br>R: I think it does a little because I think they get this sort of like mutual ground with you, but they'd spend a lot more time explaining stuff to me I feel than my partner's mum. I think that's mainly because she didn't understand, if that makes sense. I don't think it's a reflection on them trying, I think it's more the recipient. So when she didn't really understand, it's a bit flogging a dead horse sort of thing, isn't it. Like fentanyl, she didn't know what it was, she didn't know what the purpose was, and it didn't matter how many times they told her this is for that, she never quite understood, if that makes sense […] So I think it's more maybe if you can understand the information they probably will spend more time explaining it rather than someone that doesn't understand. **[Family18_L3]**<br><br>No. I don't think they are. And the physio was great, because they'd speak to all of us. Rather than just speak to my dad, they'd speak to all of us. The doctor not so much tried to speak directly to my dad, me and my brother or my partner have to actively involve ourselves in that conversation, ask some questions, which I don't mind doing. But I'd prefer to be told to me. So we know what's going off. Even though I think they already know that my dad's… his mental health state is not stable. So they should be talking to us anyway. **[Family20_L2]**<br><br>R: I think a lot of the decisions haven't really included us, or like me and my father, sorry, as such, really. It's more, especially on the critical care unit, it's like, as I touched on earlier, it was very much said, this is probably what's going to happen, and these are the medications, and gave us a bit of information about it. But with the nature of the unit itself, I think there's, kind of, the expectation of, with it being such a serious scenario, they're more giving you, like, this is probably what we're going to give her. Rather than being included, and you're deciding, or helping us decide.<br>**[Family29_L3]**<br><br>I: But I'm really intrigued about this taking people away from their job. […] In what way do you feel their job is not talking to you, their job is doing something else, injecting something, or doing a dressing, or going to see a patient?<br>R: It's just, how I put it is like, sometimes you feel that, like, the nature of how things are structured at the moment, sometimes you get worried that you're taking somebody away from what they're doing.<br>I: Yeah, yeah. So, you're using up time that could be spent elsewhere?<br>R: Yeah, that's, kind of, more like what I'm going for. Because, as I say, I've never felt like I couldn't, but it's more like […] how they're functioning at the moment, you just feel like, oh god, like, if they're doing something, you do feel like you're taking them away from that, that could be done now, rather than later. And you don't know what's coming around the corner, as well, and you feel that, yeah, if you take people away from certain things, you're feeling just a little bit guilty. Especially when somebody is into, like, even changing all the medications, and going back to, like, obviously what this is about. […] You feel like you're taking them, or distracting them a little bit, which makes me feel a bit guilty.<br>**[Family29_L3]**

	**Different ways family and friends can be involved including levels of involvement based on patient preferences, patient ability to engage etc**	R: Well, no, not really because she's, we've got kids, she's busy, I'm busy, well, I'm ill. She takes the kids to university, they're at uni. The kids are at uni and they're working, as well as working. You know how it is, kids work part-time and they study and she's looking after the household and she's trying to look after me and it's at least, too much, too trouble for her. If that makes any sense? […] And she wants to listen, yeah, she talks to the doctors and then she talks to me, then she is, she says then, she doesn't know what to think. Or what's the outcome. So, what are you going to do about it now?<br>I: Right. So, she does have conversations with the doctors?<br>R: Well, yeah. But only if I'm present.<br>**[Patient07_L2]**<br><br>Maybe relatives should be asked, are you aware of what medication your mum is on? Would you like to know? Though I would feel confident asking, not everybody would, would they, and not everybody would know that it is their right. Actually, to be honest, if I had been asked that question, I would say, yes, please. It's just not occurred to me. Had we not been having this conversation it would just not occur to me to kind of question, kind of like, where are we, what are we having? Yes, I think it would be really useful and good practice to ask an open question like that, you know, saying, are you aware? Would you like to know?<br>**[Family11_L3]**<br><br>I: One of the things you've talked through this is saying you're very prepared to ask questions, but do you think the prompt should come from…should it be down to the patient or the family members to say, well, what's happening? Is it actually better if it comes down and they say, what would you like to know or do you know?<br>R: I felt that when I was on ICU that I got the right information. Yes, I think I did because I think emotionally at that point there's probably only so much that you can cope with when you've got somebody you love so much that is in so much…I felt that what they gave me was just about probably what I could absorb. I probably…now, you know, and I guess only because of us having this conversation about my mum saying, oh, what are these tablets I was having this morning, so I guess, maybe, I don't know, maybe more information now. I'm not sure. I don't know really but, no, I think during her time in ICU, I thought the staff had got that right, I do. **[Family11_L3]**<br>I: So in terms of then the information that's coming to you do you have to search for more? Do you have to ask more questions, or have you had to ask more questions since he's been on the ward?<br>R: Yeah. I do ask, because when I visit him in the morning the first thing I do as soon as I see a nurse I'll ask about his CRP levels, his pain levels and your blood results and what's going on at the minute. That's the first thing that I ask usually. And, to be fair, they will tell me what the levels are. But again in intensive care when I used to go and ask a level they'd say oh, but it's been trending from this and this is a good incline, they'd give me a bit more information, whereas they'll just tell me a number, basically. So I think it's harder to get the information because you do have to look for it, you have to ask a lot. Like today, for instance, I've been asking…because basically he's been in intensive care for all this time and then he's been on the ward, but now he's only having IV antibiotics, he's looking after himself, he's got a lot steadier on his feet, he's eating and drinking fine, kidney function's a lot better. So he is a lot better. So it's just trying to find out what the plan is now. Because obviously he's been in hospital nearly a month now, he's desperate to go home. **[Family18_L3]**<br><br>She did say many people don't ring up…because we'd call in the night. We used to have a direct line to his room, which I think is a brilliant idea. So if I felt a bit uneasy in the night, I'd give them a call. When he was critically ill they did his bloods every two hours, so then I'd be able to call at night and I'd ask his level of his blood, and she said normal family members don't ring up and ask what is this level, what is that level. So I think it is hard to know if you would get that information otherwise or not. **[Family18_L3]**

		R: I think on reflection, I think…I did express this to the nurses, I think for me as the partner having very strict visiting times, I think that's not very good for someone who is in intensive care just because I feel like every hour something can change, and I think if your loved one is having a bad morning, for instance, and you have to leave them because you haven't got a visit booked, I think the best way to keep informed is to keep that conversation…to keep being around, to keep being involved.<br>I: So have you been finding where there have been conversations between your partner and the medical staff have you then gone back to check or been like a go-between in there because of that?<br>R: Yes. Especially in the first couple of days when he weren't 100 per cent, if by any means I wasn't there when the doctors came round, he would struggle to give me that information. He would say oh, they said I might be able to go home in the next couple of days. I'm like oh right, what did they say about your antibiotics, IV, what they going to do about that? Oh, I can't remember what they said. But obviously when it's going to be me looking after him I need to know, but what are they going to put in place to make sure that he's going to be all right, what are they going to do about this, what are they going to do about that. So I think that is a bit difficult. **[Family18_L3]**<br><br>I: Yeah. And how do you feel about the way you've been, or not been, if you like, how do you feel about the level of the conversations or the amount of information you've been given about these medicines? Has it been…you know, what's that been…?<br>R: Well, I think that my wife has been here for many hours over the last three to four weeks […] She's spent a lot of time, from, like, eight o'clock in the morning while eight o'clock at night. And although it might…and some of it might have gone over her head, a lot of the information that was…couldn't be given to me, would have been given to her. […] She…we discussed it last night and she was like, really don't remember, I really don't remember the, you know, what drugs were what, and…and so on and so forth. But the actual conversations one's had between the nurses and pharmacists, you know what I mean. So obviously they couldn't talk to me because I was unconscious. […] So there would have been a lot of information passed on. Some of the nurses spent a hell of a lot of time, you know, helping iron out questions and that sort of thing, to try and ease (name of patient's spouse) mind **[Family19_L3]**<br><br>I: So basically, they're talking to him but then when he's mentioning things you can then intervene, as it were, and… So the next question actually is about decisions, about your family members' medicines. So I mean in what way do you think you're included and involved in any decisions about that?<br>R: In what way? I think…I don't think… Yeah, my dad's mental state is not good. […] Not good at all. And I think judging on how the doctors speak to us and the physio speaks to me and my brother, or my partner, is that they don't leave the full decisions to my dad because he's not capable of making the decisions solely himself. Hence why they like to speak to us as well. I'm still waiting to speak to the physio but I can't get in early enough from work to speak to them at this moment in time. I have to probably make a phone call or something like that. But I think that's the main reason why we'd be so involved in answering questions of any decisions that are made. And I feel myself anyway, knowing what my dad is like, I would like to be rather closely involved in any decisions made. Because at one point they were just going to send him home, discharge him at the beginning of the week, like Monday, Tuesday. But you can't do it. He's not fit to come home. It's not safe.<br>**[Family20_L2]**<br><br>I: Yeah, well, I'm sure, as you say you're having conversations with people about that, and hopefully that might resolve itself, I don't know, I have no idea, not being clinical. But so, in some respects then, thinking about those decisions that have been made, in what way do you feel that you've been included and involved in any decision making?

		R: I don't think I have been involved in any of the decision making, but that doesn't bother me. She's got her husband, and she's got her two sons, although I kind of have taken the lead a lot of the time, through them asking me to. I think we've just allowed Critical Care to get on with making her better in whatever way they need to […] And it was having that trust in Critical Care. I'm very experienced with (name of hospital) and it's a great hospital, the staff are fantastic. So we've just trusted that they've made the right decisions for (name of patient) when she was unable to do so. So I've not been too concerned that I haven't been consulted, they're the professionals, I don't like people telling me how to do my job, so I let them get on with their job. **[Family21_L3]**<br><br>R: I know, yeah. And back to the ward on Saturday, the third, so he was in critical care for a week, gradually getting better. And then on his transfer to the ward, he was totally with it, you know, he was back with us. So I asked him yesterday, because I knew I was going to do this interview today, about his medication and everything, and he knew everything, all the medication that he was getting, from the nurses. So he was able...it wasn't a conversation I needed to have with the nurses, if you know what I mean. […] Because he knew what he was getting, he was being told what he was being given and he knows what he should have been taking and shouldn't have been taking. And he said that everything that he was taking was right, it was what he was on before. Now if it had been my mum who had Alzheimer's, we lost her about six years ago, then I would have been more involved, obviously, because I would have been the one, or my dad, rather, would have been the one that was in charge of her medication. So he would have had to have had those conversations with the nurses, do you see what I'm saying? **[Family23_L2]**<br><br>I: So it's not…they were saying, oh, this is your antibiotics but they weren't providing you with further information like exactly why they were doing that and for what reasons they were?<br><br>R: Yeah, that was pretty much for a lot of it. A lot of it I had to say, so the blood thinners, for example, (name) is currently on blood thinners. Now I know in the healthcare profession that blood thinners, if someone's laid on the bed, it's quite common to be given, especially in hospital after big surgery, to avoid blood clots. However, his wife doesn't know that. So as soon as they left, the first thing she asked me was, why is (name) having blood thinners? She has no idea. So in her head, she thinks now (name)'s, you know, potentially a high risk of clots but don't know what or why, is that because of the surgery, is that because of all this? But she had no…so I had to sit down and explain it. And then it's just watching that fear go away that…you know, so I know why they're doing that but they didn't explain why they were doing that, I had to be the one to tell his wife that. **[Family25_L3]**<br><br>I: Yeah. You've actually used a phrase there which is I find it really interesting about 'depth of explanation'. You know, is that what would be useful to have that, sort of, further…?<br>R: I think so. I think knowledge is power. I think if you understand what you're facing and what the plan is, you can better prepare for it, you can better understand what to expect for both negative and positive. You don't panic if something bad, you know, goes, if you're prepared for the potential of that. Instead, you get slapped with something negative that happens and actually you think you've just jumped back 10 steps but really in reality, it's quite normal. But you don't know that it's quite normal until it happens, if that makes sense. **[Family25_L3]**

		And because, why I obviously seek and ask these questions. But I think, from a standpoint of, with my father, when he ended up coming with me, a lot of the information sometimes would have gone over his head, in regards of, it wasn't quite explained in a way where, if you didn't have even a little bit of knowledge beforehand, it could be something that doesn't quite meet the expectations of somebody who wouldn't quite understand. Or, like, if you didn't have any understanding of, like, what these things were, sometimes it did, it was, like a point where my dad wouldn't understand why she was on it. And it was very much, you'd have to give an explanation. Maybe if there's somebody else, obviously, in my place, and stuff, I just feel that, maybe a little bit more information would be good. […] But I think it's very much difficult to do presume, obviously, because it was me who was there, I don't really know. But from my father's perspective, a lot of it I'd have to relay to him, or at least reassure him about, if that makes sense at all. **[Family29_L3]**<br><br>R: […] I feel that, if they seek out the family, in order to, I mean, just make sure that there was certain medications that they were aware they were on beforehand. Because I know, one of the major ones that I don't think I've mentioned at the moment, is she's on citalopram. […] And even though, obviously, at the time, that wasn't really something that was really entering the minds of anybody, really. But now she's come round, and now we know that she's been off it for quite a duration, it would always be handy just to be able to have that discussion of, these are all the medications that she's had, this is all the medications that she has a prescription for. And especially again, when it came to the COPD, I think a lot of it, even coming down to the medication that she was having, if I hadn't mentioned it, I'm not sure whether they would have actually known how many issues she's had with it in the past. […] I think, just getting a general health overview, just even from the family, like, it just, I feel personally, would help. […] I feel like it would just be nice to sit down with the family, and ask exactly what she's been on, exactly what the potential emergency scenarios have been, that could be contributing to some of the issues that the patient is having currently. **[Family29_L3]**
	**Patient (or F&F) confidence in their ability and capability to engage with health professionals and to challenge decisions**	When I went on my statin at my local practitioner, and I said, what's this for? And the next thing, this girls says, oh, they're ready at so and so tomorrow for you. I said, what will? She said, these statins. I said, woah, love, I said, just you reel that in, I said, first of all, I'm not under that pharmacy. I said, secondly, I said, I want to speak to a medical professional. I want to know why, and I want to know the background on these tablets. And I give them their due, like, the head nurse, what do you call them, charge nurse or something, she phoned me up and she told me what she was going to give me. She said, it's nothing to do with your, what do they call it, all the fats stopping your blood, it's not for your cholesterol, it's just for this…for my heart. I said, what about the side effects? She says, well, it can cause some numbness in your legs, et cetera. But if that happens, let us know and we'll change the statin. Well, fortunately, the statin I've got has given me no…what I know of, no side effects […] Maybe I'm a bit sometimes too forward, but if people are giving me something, I don't take a young girl on the phone desk's word for me. [Inaudible 00:24:43], hang on a second, let me find out why, what it's for and where it's for. **[Patient01_L2]**<br><br>I: Right, okay, so it's about when you get the information leaflet, they want you to look at that carefully and think about that. Do you feel that you've been involved in some of these decisions, say you've talked with your mum, and you've talked with the doctors, how do you feel included and involved in what's happening with those?<br><br>R: Well, everyone has always spoken to me about everything that is happening around me and always made sure that I was aware of it, even in a confused state and that way, I was able to make executive decisions with them, but I wanted them to make them with me because of the confused state, if I'm honest.<br>I: Right, okay, that's really helpful and so you feel that there's been a discussion, you feel that…you said you feel you've been included in all the decision-making, has that been important to you then?<br>R: Yes, I like having a sense of control.<br>I: Okay, so do you think it's important to have a sense of control around your medicines you're taking, is that because of what has happened to you?

		R: Yes, because it shocked me so much when I got carried away, I'm way more strict on that side of things now. **[Patient06_L2]**<br><br>I: Right. And what do you know those?<br>R: I don't know, most of them, I don't know nothing about them. Quite a lot [heart's 04:55]. I'm on some eye medicines and there's some high cholesterol, low cholesterol and there's quite a lot of tablets, I'm on.<br>I: Right, okay.<br>R: And I need to, I need to sort it out, with my doctors of what they are. But I need to be on them. That's what [voices overlap 05:16]…<br>I: The doctors in the hospital or doctors when you get back to your GP or…?<br>R: Doctors when I get back to my GP. **[Patient07_L2]**<br><br>I: No, no, it's brilliant, it's fine. No, that's great. No, yeah, absolutely. No, and so if you've, have you said, oh, I don't, any time, have you said, oh, I don't want to be on that medicine or I don't want to do that or can we do this? Have you pushed anything like that?<br>R: Because I don't know nothing about it or about the medicines, I don't want to say anything. Does that make sense, until I know what the medicine is. That's what I'm planning to do. That's what me and my wife are planning to do. When I leave the hospital, I'm going to find out what medicines I'm on, what they're for. Because I'm on about 28 some form of different tablets. At least 28 and that's quite a lot for me. And it depresses me. It depresses me. So, that's what we're going to go through. We're going to go through each one, even if the doctor likes it or doesn't like it. I, we want to just going to see what's happening. **[Patient07_L2]**<br><br>I: No, that's, that's interesting. One of the questions we've got here is how do you think patients should be best involved in discussions about their medicines? But you've, sort of, answered that, haven't you? Is it that, is it going to your GP? Is that how the best…?<br>R: It's literally going to your GP, explaining yourself. Explaining the medication and explaining to the doctor, is it his fault or is it my fault or is it any of the doctor's fault that's put me on the medication? Or is it another consultant and just tell me what to do from now on. Do I still carry on with it because, at the end of the day, it's just, it's too much. **[Patient07_L2]**<br><br>Yes, and I don't think I probably asked searching questions. It's a very strange thing when a relative is so seriously…and they'd say to me, is there anything you want to ask, and you've almost got…and I almost…as the weeks went by because she was in there for some time, I would almost say, is there something I should be asking? I guess, they don't know how much information to give to relatives so I don't know that I always ask the most pertinent of questions. You know, you're just kind of like surviving day by day really. **[Family11_L3]**<br><br>I: I mean, do you feel you would like to be more included, involved in the…<br>R: I don't think so, no. I'm more very vocal if I can…the physical wellbeing and all that, I would be very vocal if I thought that my mum wasn't happy and I guess, on the medical side because I've got no medicine knowledge at all that I just put my trust in them but I do feel that if I did ask the questions, then I would get the answers. I feel confident enough that I would ask either the doctors or the nurses so I don't feel inhibited in that way. I think I'd probably just blindly trust them with the medication. I think that's where that's coming from **[Family11_L3]**<br><br>I: Do you think that's how family members can be involved or perhaps are best involved in that sort of way or perhaps to rephrase that how do you think family members can be best involved in these sorts of decisions? Do you think, as you say, leave it to the professionals?

		R: Maybe relatives should be asked, are you aware of what medication your mum is on? Would you like to know? Though I would feel confident asking, not everybody would, would they, and not everybody would know that it is their right. Actually, to be honest, if I had been asked that question, I would say, yes, please. It's just not occurred to me. Had we not been having this conversation it would just not occur to me to kind of question, kind of like, where are we, what are we having? Yes, I think it would be really useful and good practice to ask an open question like that, you know, saying, are you aware? Would you like to know? **[Family11_L3]**<br><br>I: One of the things you've talked through this is saying you're very prepared to ask questions, but do you think the prompt should come from…should it be down to the patient or the family members to say, well, what's happening? Is it actually better if it comes down and they say, what would you like to know or do you know?<br><br>R: I felt that when I was on ICU that I got the right information. Yes, I think I did because I think emotionally at that point there's probably only so much that you can cope with when you've got somebody you love so much that is in so much…I felt that what they gave me was just about probably what I could absorb. I probably…now, you know, and I guess only because of us having this conversation about my mum saying, oh, what are these tablets I was having this morning, so I guess, maybe, I don't know, maybe more information now. I'm not sure. I don't know really but, no, I think during her time in ICU, I thought the staff had got that right, I do. **[Family11_L3]**<br><br>I: No, right. How do you think patients can be best involved in their decisions about their medicines?<br><br>R: I think it's quite tricky these days isn't it, because a lot of people have got some knowledge of medicines and I'm very much the exception to the rule, I think I've got quite a lot of knowledge of medicines, and a little knowledge of them is probably a bad thing, stuff looked up on the internet or what have you. I don't know, it's just everyone's different, aren't they? I don't think there's a sort of general rule that you could apply. I suppose what we've got now which is let's go and see this person and see, you know, whether they are able to engage in the decision themselves or whether they are quite happy to let the doctors know best, I don't really know.<br><br>I: Yes, well, it's almost you say, yes, it's almost like an individual, personal, case by case basis, isn't it.<br>R: Yes, I mean I think most people when they go into hospital they feel as though, like, you know, do whatever they want, these guys know best, you know. […] But as and when it became a decision for me, if there was, certainly any decisions being made for me, I was certainly allowed to make it myself **[Patient12_L2]**<br><br>I: But is there a particular way you'd like to receive information from them, in terms of is it better that it's a face to face conversation, or is it better that it's… Would it be better with an information sheet, or something written down?<br>R: Yeah, I would say that maybe sometimes when we have had the conversations, especially with the surgery, they do sometimes explain things a little bit…it's a lot to take in in one go. You try and remember things after, did they say this, did they say that? I can't remember. Is that what he said? Like yesterday, because we've had that many conversations with different people, maybe some kind of way of noting it down without sitting there taking notes yourself. Maybe that might be helpful, yeah, because there's a lot of information to take in sometimes. And obviously when you don't know, where you've got no medical background, sometimes it is quite a lot to take in. **[Family15_L3]**<br><br>I asked about the …(name of patient/participant's spouse) thought it was omeprazole, but I think it was lansoprazole. Anyway, it doesn't really make…it's a proton pump inhibitor. So I asked about that 'cause I thought I recognised it and (name of patient/participant's spouse) wasn't sure what it was and…until I reminded her and then it came back, so she had been told she just misremembered it or not remembered it, should I say. So…yes, that…everything else was straightforward really. She seemed to have been given everything she should have been given. She seemed to have been given it at the right time. **[Family16_L2]**

		R: Well, I don't know. Because sometimes, it can be rather frightening, at first. […] Because he was covered with wires, and everything, and he'd got drips all over. And at first, it were overwhelming, the first night he was took in. […] And I think, if they did tell us upfront, then, it wouldn't have gone in. […] You know, as we got used to going, then we knew what questions to ask. […] Because the first couple of days, it were overwhelming. **[Family17_L2]**<br><br>R: So basically what would happen, my partner would get started on different variations of medication. He had quite a nasty infection and the cultures had not been grown from the lab. They weren't too sure actually what the infection was. So when I get there in the morning, there'd just be different antibiotics, names and different bottles and stuff. So I was quite inquisitive because I do work in the NHS myself. […] I'm not clinical but I do work in a clinical environment so I do have a bit of an understanding, so it made it a lot easier for me. But I think like my partner's mum, for instance, who doesn't have any medical terminology or anything, she found it really hard to understand what all the medicines were and what they were for. But the staff in the intensive care unit were very, very helpful and tried to explain, like this is for blood pressure, this is for this. So they were really good in explaining stuff. **[Family18_L3]**<br><br>I: Right, okay, good. Yeah. Did you prompt anyone for any details or was that discussion initiated by them?<br>R: I don't know what other people's experiences are like, but, obviously like I said, because I do know bits and bobs as soon as I got there in the morning and asked what his blood pressure medication was on, how much clonidine he was on, I'd ask all different questions about the medication because I remembered them because I used to see him every day. So if there was a new one I'd be like oh, what's that for, what's this.<br>I: So you're not clinical but you do work in the NHS.<br>R: Yeah. I'm a practice manager at a surgery. I don't work in a hospital environment […] But I've got some sort of clinical knowledge… **[Family18_L3]**<br><br>I: …one to one, as you say, to one to six or seven or eight or whatever. So in terms of then the information that's coming to you do you have to search for more? Do you have to ask more questions, or have you had to ask more questions since he's been on the ward?<br><br>R: Yeah. I do ask, because when I visit him in the morning the first thing I do as soon as I see a nurse I'll ask about his CRP levels, his pain levels and your blood results and what's going on at the minute. That's the first thing that I ask usually. And, to be fair, they will tell me what the levels are. But again in intensive care when I used to go and ask a level they'd say oh, but it's been trending from this and this is a good incline, they'd give me a bit more information, whereas they'll just tell me a number, basically. So I think it's harder to get the information because you do have to look for it, you have to ask a lot. Like today, for instance, I've been asking…because basically he's been in intensive care for all this time and then he's been on the ward, but now he's only having IV antibiotics, he's looking after himself, he's got a lot steadier on his feet, he's eating and drinking fine, kidney function's a lot better. So he is a lot better. So it's just trying to find out what the plan is now. Because obviously he's been in hospital nearly a month now, he's desperate to go home. **[Family18_L3]**<br><br>Well, I came down here on Monday, for a skin graft. Sorry, no, I came down here on the Friday, had the skin graft on the Monday. So now I've got the dressings coming off tomorrow. So when they come off, obviously I'm going to be asking what the situation is, what I need to do dressing-wise. […] And all that sort of thing. I mean, I'm obviously…well I'm saying obviously, [inaudible 0:15:35] but I'm quite rooted now, I'm up and about to a degree, I'm [inaudible 0:15:40]. So all being well, you know, at the end of the week I'm hoping to be…tootling off home, kind of thing. You know what I mean? […] You know, in this next week, there's quite a lot to happen. […] So I'm capable now of asking those questions [voices overlap 0:16:03].And getting the answers that I need, you know. **[Family19_L3]**

		I: And when you've been having these conversations with people, you said about the nurses there, and the Nursing Sister, have you initiated anything, or have they come to you?<br>R: I think it was a bit of both really. They've come to us at times, and then if I've seen the nurse sat at the desk and I've got a question, I would approach them.<br>I: Yeah, so, and have you been happy to do that, do you feel confident to do that?<br>R: Yeah. I'm happy to do that, I've had a lot of involvement with hospitals with our mum over the years, and my dad, so yeah, I'm quite happy to approach any member of staff and say, do you know what's happening, or, can you answer me this question? **[Family21_L3]**<br><br>To be honest, the medication, his medication wasn't a problem because, like I say, they had everything on the screen what he took and they managed it and they, you know, I knew they were managing it and my dad knew he was managing it. On the transfer to the ward my dad was fully with it by this time, because he spent nearly, I think he was admitted to critical...I've written it down actually, he was admitted to critical care on the 27 May, which was his birthday, believe it or not, his eighty sixth birthday. […] And then on his transfer to the ward, he was totally with it, you know, he was back with us. So I asked him yesterday, because I knew I was going to do this interview today, about his medication and everything, and he knew everything, all the medication that he was getting, from the nurses. So he was able...it wasn't a conversation I needed to have with the nurses, if you know what I mean. **[Family23_L2]**<br><br>R: I know, yeah. And back to the ward on Saturday, the third, so he was in critical care for a week, gradually getting better. And then on his transfer to the ward, he was totally with it, you know, he was back with us. So I asked him yesterday, because I knew I was going to do this interview today, about his medication and everything, and he knew everything, all the medication that he was getting, from the nurses. So he was able...it wasn't a conversation I needed to have with the nurses, if you know what I mean. […] Because he knew what he was getting, he was being told what he was being given and he knows what he should have been taking and shouldn't have been taking. And he said that everything that he was taking was right, it was what he was on before.<br>**[Family23_L2]**<br><br>R: Oh, yeah. We were actually there during the transfer because he actually got transferred onto the ward while me and my sister were visiting him. So we actually went down to the cafe and got a coffee while they did all the, you know, the moving from one to another. And then we came back to the actual ward when he was all settled on the ward. But, yes, he was totally with it, I mean, it he'd literally been a week in there and he was already, just before he came out of critical care, he was already sat up and talking and he was already with it, you know. A lot of his pain medication that was making him a bit strange at first, you know, at first, with the amount of painkillers and stuff they're pumping into you it makes you a bit, well you're in and out, aren't you of, sort of, not consciousness exactly, but he thought the bin was a fridge and things like that. But once that was gone, and that was more or less gone by the time he was transferred, or most of the really, like, heavy drugs had gone by the time he went back to the ward, he was [inaudible 00:13:18], sat up, compos mentis, talking about everything, you know. […] So he knew whatever medications he was given, there was no need for me to intervene and ask about his medications because he would have been on it himself because he's that type of person, he's a worrier, do you know what I mean? So he would have made sure that he was getting everything he should have got. **[Family23_L2]**

		R: To be fair, it was a mixed emotion, if I'm being honest. You know, inside I'm broken because my son is in agony and there is nothing that I can do. I'm angry because I know there's people that can do something. I know that they're stretched and I understand and totally appreciate that, however, I just never felt that they were on our side. So it always felt like a bit of a fight. I had a consultant's words to me, when I…because to me, they weren't checking (name), they weren't…I'm very clued up on healthcare, so I work in the healthcare industry. So, you know, they'd done no pressure care with (name), they'd not come in to check for anything. His feet were overhanging off the bottom of the bed, they'd not offered to take him up the bed. There were so many things going on that it just built up over this time period of (name) being in the state that he was in. I said to the consultant, it's institutional abuse, which it was. And he said, I completely agree. And that to me was just like the icing on the cake because then he said, however, we are short staffed. But, you know, for me, how is that the patient's fault? Not just my son, I don't doubt other patients are probably in the same boat, however, you know, it shouldn't be that way for anybody. So it was such a mixed emotion, to be honest. It's quite heartbreaking as a mother to watch your son in that much pain to the point that you feel like you have to blow your top just to be felt like you've been heard. **[Family25_L3]**<br><br>And because, why I obviously seek and ask these questions. But I think, from a standpoint of, with my father, when he ended up coming with me, a lot of the information sometimes would have gone over his head, in regards of, it wasn't quite explained in a way where, if you didn't have even a little bit of knowledge beforehand, it could be something that doesn't quite meet the expectations of somebody who wouldn't quite understand. Or, like, if you didn't have any understanding of, like, what these things were, sometimes it did, it was, like a point where my dad wouldn't understand why she was on it. And it was very much, you'd have to give an explanation. Maybe if there's somebody else, obviously, in my place, and stuff, I just feel that, maybe a little bit more information would be good. **[Family29_L3]**
	**Patient (or F&F) perspectives of health professional willingness to engage, co-operate and work in partnership, including confidence that they will**	But the guy was very, very clear. And he spent time with me, he didn't just come in, bang, bang, bang, bang, bang, bang, make just ten bullet points and go out. He came and he took his time with me. **[Patient01_L2]**<br><br>R: Yeah. I understood it. It's more hands-on and you feel [with the NHS at the present time, like 00:08:46] but you feel a person and not just a patient. […] Or a number. You know, it's like my specialist came round, the doctor who did the operation – he's done me three times now, like. And even my nurse, I have a nurse called (name), who does anything and I can phone her any time of the day, seven days a week. Even she came round three times to see me. Twice in ICU and once on the general ward. And my doctor came round every time, every day. No, I tell a lie, he didn't come Sunday. But he came on the Saturday morning, he was there on the Monday and there on the Tuesday. **[Patient01_L2]**<br><br>R: They just gave me the goods. You know, if it was something new they'd tell you what it was anyway, and if it was something which was a continuation, so they said, this is such and such, you know, they always said. And the best thing about that was they reiterated…if you asked they reiterated what they said at the first, you know, because obviously sometimes you're so far out of it **[Patient05_L3]**<br><br>R: This time with the, it seems to be better than I was in before. Because the doctors come round in the morning and they'll come and they'll actually talk to you. And they'll talk to you and say, well, this is what we want to do. We want you out by this much warning. See, this, a doctor might then want to see this. I didn't have this before. If that makes any sense?<br>I: Yeah, yeah. And what do you feel about that? Do you think that's good?<br>R: That. Yeah, yeah, it is good, yeah. It seems to be better. It gives you the confidence because, well, hold on a minute, they know what they're going to do. They've started to bring me into the conversation with themselves and that's it. **[Patient07_L2]**<br><br>R: I would say a lot of that was explained by a sister that was doing…when Mum was really poorly and she was, kind of like, working with her constantly there was a sister that worked with her and she explained that to us in quite some detail.<br>I: So it was basically around nursing staff then who would do that.

		R: Yes, I would say most of my conversations were with the nursing staff because they were the ones that were doing the main care for her. I mean, had I requested a conversation with a doctor that would have been…you know, that was possible but I found that I was happy with the information that I was getting from the nursing staff, particularly this…this particular sister was really good. **[Family11_L3]**<br><br>I: Do you think that's how family members can be involved or perhaps are best involved in that sort of way or perhaps to rephrase that how do you think family members can be best involved in these sorts of decisions? Do you think, as you say, leave it to the professionals?<br>R: Maybe relatives should be asked, are you aware of what medication your mum is on? Would you like to know? Though I would feel confident asking, not everybody would, would they, and not everybody would know that it is their right. Actually, to be honest, if I had been asked that question, I would say, yes, please. It's just not occurred to me. Had we not been having this conversation it would just not occur to me to kind of question, kind of like, where are we, what are we having? Yes, I think it would be really useful and good practice to ask an open question like that, you know, saying, are you aware? Would you like to know? **[Family11_L3]**<br><br>I: So basically, you are asking questions but they're also volunteering stuff to you if you're seeing them. But if they're volunteering stuff to your mum, you might then go back and check back with them. That's interesting.R: Yeah. It's like the surgeons and doctors, I think they only seem to be there in the daytime. Obviously me and my sister have been going up to see her and we both work, so we have to go up late and there's something not always a doctor around. But there is usually somebody that we can ask, but I suppose they're looking after so many people, they can't really just come round and explain everything that's already been explained once, which I understand. I: And in terms of those explanations you've been given, what do you feel about those? Have they been clear enough, or have you had enough information, if you like, perhaps?<br>R: Yeah, I think so. There's nothing really that we've been left not knowing, to be honest. **[Family15_L3]**<br><br>R: I feel like on reflection the intensive care nurses do an amazing job. They don't reassure you in the way, they don't say your loved one is going to be fine, because they are open and honest and they will tell you that they can't give you that reassurance, despite how desperate you may be for it. They don't give you any false pretences that your loved one is going to wake up tomorrow and they're going to be fine, because they can't tell you that information, which I understood from the beginning. But I think they reassure you in the way when, like, for instance, he was put on a ventilator and for me that was the worst thing that could happen, just being put in an induced coma, I thought that was meaning he's not going to wake up again sort of thing. I didn't understand the actual process of it. Because you only hear about your friend's aunty who was on a life support machine, and they had to turn it off because she was unresponsive. You don't hear about the amazing things that the intensive care people actually do to save lives. So I think when they sat us down and explained the process of what would happen to his body and what medications they'd be using and stuff like that, it did make me feel quite reassured that it was a positive thing as opposed to a negative. **[Family18_L3](STAR QUOTATION)**<br><br>I: In terms of the way people would talk to you then, how do you feel about how you were…how these things were discussed with you and how the conversations went?<br>R: In regards to in-depth information is what I wanted, pretty poorly up until the point of speaking to the doctor in respiratory, on the respiratory ward. I mean the guys in critical care were fairly vague with what kind of medicine they were giving. **[Family20_L2]**<br><br>I: Yeah. And how do you feel about any of those explanations that you've been given?<br>R: Yeah, it was fine. I think it would have been nice to have spoken to somebody about the oramorph beforehand, but if it's happening in the night, obviously, it's not something they're going to phone up and chat to you about, they're just going to make that decision, which I totally understand. **[Family21_L3]**

		R: But if ever I wanted to ask a question, if ever I was concerned about something or I wasn't sure what was going on, the staff on critical care were amazing and would always take the time to sit down and go through it all with me again. And even though I couldn't communicate either because I had a trachea in that prevented me from speaking, even to the point where the staff they knew that I was getting frustrated because people couldn't lip read particularly well, so they got me a board so I could write on it and communicate that way. So, I feel like…yeah, it was a bit strange because obviously choices were out of my hand to begin with, but when I was communicative and once I had questions, people had all the time in the world to explain to me what was happening. So, I feel like I've had as much communication as I possibly could have.<br><br>I: And you said they facilitated even when you couldn't speak with the board as well, which is fascinating, isn't it? Very helpful obviously.<br>R: It was such a small gesture that was made by…I think…she wasn't a nurse she as one of the support workers, such a simple gesture, I just said to her before I left critical care unit, I know it took you minutes to go and find this thing but, I said, you made a world of a difference, because I didn't realise…obviously my role in a job and I'm also a volunteer, there's a lot of communication involved, and I didn't realise how restricted it is when you can't just talk. And that simple gesture of her taking two minutes to go and find that board to give to me, it made…well, it made my month basically, it gave me my month back and I just said to her, that was amazing and thank you so much, you have no idea the difference you've made. I definitely let everybody know on the ward that they did fantastic. **[Patient24_L3](STAR QUOTATION)**<br><br>I: You said there you feel you've been involved in the decisions, with whom have those…you know the conversations around those decisions…who have you been talking to or who's been talking to you about that?<br>R: Yeah, it's just the nursing staff that administer the medications. Obviously they knew what I was on when I came from critical care and they just, is that working for you? Are you happy with that? Do you want this? Do you want that? So, they asked me every single time, it's not a, here's your medication you must have it. We progressed it a little bit in that speech and language therapists weren't completely happy with my swallow, so rather than trying to swallow tablets now, I get like a…what do they call them? Like an effervescent tablet, isn't it?<br>I: Yes.<br><br>R: So, everything's liquid form now. But, yeah, it feels like a choice, like it's a, you must take this. This is what it is…I also have an injection in my tummy once a day as well, I think that's a blood thinner because I'm not moving around as much. So, that's an option. There's also an option to have…oh what do you call them when you have the face masks with the…a ventilator, essentially like an inhaler. That's also my decision but they always try and give it me when I've got visitors, so I didn't have that for the first few days. But that was to clear stuff off my chest and every day I feel my voice is stronger. So I had this conversation with the nurses and I think they all tend to agree with me and we'll kind of like come to an agreement that it's not really necessary. So, yeah. **[Patient24_L3]**<br>I: And that's really, really interesting. And in terms of those conversations, how do you feel about the actual conversations that was happening? The ways that people have talked to you, if you like?<br>R: Yeah, I feel like everybody's…we've all got one goal in mind and that's to get us better. They will listen if I've got concerns, if they don't have the answer they'll go and find somebody that does. Yeah, it's been a breath of fresh of air being on the ward. Yeah, everybody's very respectful, very mindful that you are an individual with feelings and thoughts. And if you've got questions and, like I say, if they don't have the answers they'll always find out for you. **[Patient24_L3]**<br><br>I: Do you feel that when you've made that, do you feel you've been supported by what you…your choices within that?<br>R: Yeah, I would say that as well I am very conscious of that fact that I'm not medically trained and that these guys know a lot more than I do. So, as long a somebody explains something to me and it makes sense in my head, then I'm happy to go with their recommendation because they're the professionals, I put my trust them, and nobody's seen me wrong yet. I know I've said it's my choice and it does feel like that, but also I'm happy to be led by a medical professional because they know a lot more than I do. **[Patient24_L3]**

<table>
<tr><td></td><td></td><td>I don't think so. I think the main part of it all really, I just wish the communication and the quality of everything between the ITU and, you know, down to the ward, that level of care shouldn't change. I really want that highlighting, it shouldn't. I know they can't give one-to-one care and I totally respect that, working in the healthcare industry I know that first-hand. But that quality should never drop, it should always be top quality when they're with that patient. And half the time, you know, you feel as though they don't even know the person that they're treating. And that's the scariest part of it, especially as a relative, to watch that happen. [Family25_L3]</td></tr>
<tr><td></td><td>Patient network of support and the ways family and friends engage on behalf of patient (or not) - or patient preference for this</td><td>R: Basically, I was originally on antidepressants and questionable pain relief and then I got put on safe pain relief and told that the antidepressants definitely weren't working because if I wanted to drink again, it probably…yes. […] Basically, one of my mum's friends was on antidepressants and she said she didn't really get the urge to drink when she was on them, but I still did. So, it feels as if it wasn't really working well with my body.
I: Right, okay, and are they taking you off the antidepressants now or have they talked to you about those?
R: It's mostly been advice not to take them, but by choice I'm not going to take them, there is no point taking something if it's not going to work, that's just going to put my liver at further risk.
I: Yes, precisely, absolutely so then you've made that decision then?
R: Me and my mum together. [Patient06_L2]

Interestingly though you're talking about medicine, she said, my mum did say to me today, she said, I don't know what medicine they've given me this morning, (name), she said, and she talked about the tablets so she's not been told at the moment what medicines she's having and what it's doing. I mean, I'm sure if they asked but they're not actively telling her and I guess, I could have at that point said to her, Mum, would you like me to ask? One thing that I do notice is that when they do do their rounds, she likes me to be there and fortunately I have been there because they do their rounds in the morning. This is only what happens on this ward and they tend to be a team of people that come. So you've got your main consultant and you've got presumably your trainee doctors and so they all kind of like arrive on troupe because they're kind of like learning and she almost panics and says to me, (name) you will stay in the room. I think it's just…my mum's always been very fiercely independent, she's a retired teacher and she's very articulate and things like that, I think she just finds it a little bit overwhelming now and wants me to be there to listen to possibly ask the questions, you know. Yes, I don't know.
I: That's interesting as well because I mean, one of the things around that, sort of, I suppose, decision making, who's making those decisions?
R: Yes, because they almost look when I…because initially they'll ask me to go out because there is a bay of four people and they obviously don't want me to hear what's being said for the others but I have to ask, well, my mum has to ask, and some of them almost look at me like it's an inconvenience, why would you possibly want to come back in? I'm thinking, well, actually, it's not me, my mum's just said to you, she wants me in. So it's not me being difficult, my mum says, can my daughter be in? [Family11_L3]

I: So you were involved in those conversations as well.
R: Yeah. I've been going up to see her at night because I work during the day, but anything that gets [inaudible 00:08:59]. In the case of my mum, she sometimes doesn't always take everything in, because it's a lot of information to take in. And obviously when I go up at night, there's always usually someone to speak to, if it's not too late, and they'll reiterate everything that they've said. And then I can try and feed that back to my mum in a way that she might understand it a bit more. [Family15_L3]</td></tr>
</table>

		R: That does the feed that goes into the PICC line, because I think that has to be changed every day depending on blood tests and things like that. So he's been coming round every day, but it's usually in the daytime, so I'm not usually there. So just because of that reason, I sometimes do have to ask questions. And to be honest, my mum can remember quite a lot of it because she does tell me, but if there's something that she doesn't quite understand, I do sometimes have to try and find someone to just ask. **[Family15_L3]**<br><br>I: Yeah. And are you going, are you visiting regularly in terms of, and so, do you get to see the staff regularly, and have regular contact with them?<br>R: I go every day. […] And whenever I see, like a ward sister, I seem to ask them more than the nurses, because they seem to know more. […] And the nurses are very busy. So, if I get worried about anything, I will look for a nurse and ask them. […] But they're in and out to him all the time, anyway, so you can always ask a question. **[Family17_L2]**<br><br>I: Right, okay, good. Yeah. Did you prompt anyone for any details or was that discussion initiated by them?<br>R: I don't know what other people's experiences are like, but, obviously like I said, because I do know bits and bobs as soon as I got there in the morning and asked what his blood pressure medication was on, how much clonidine he was on, I'd ask all different questions about the medication because I remembered them because I used to see him every day. So if there was a new one I'd be like oh, what's that for, what's this.<br>I: So you're not clinical but you do work in the NHS.<br>R: Yeah. I'm a practice manager at a surgery. I don't work in a hospital environment…<br>I: Right, yeah. So you have lots of…<br>R: But I've got some sort of clinical knowledge… **[Family18_L3]**<br><br>I: Yeah. And how do you feel about the way you've been, or not been, if you like, how do you feel about the level of the conversations or the amount of information you've been given about these medicines? Has it been…you know, what's that been…?<br>R: Well, I think that my wife has been here for many hours over the last three to four weeks […] She's spent a lot of time, from, like, eight o'clock in the morning while eight o'clock at night. And although it might…and some of it might have gone over her head, a lot of the information that was…couldn't be given to me, would have been given to her. […] She…we discussed it last night and she was like, really don't remember, I really don't remember the, you know, what drugs were what, and…and so on and so forth. But the actual conversations one's had between the nurses and pharmacists, you know what I mean. So obviously they couldn't talk to me because I was unconscious. […] So there would have been a lot of information passed on. Some of the nurses spent a hell of a lot of time, you know, helping iron out questions and that sort of thing, to try and ease (name of patient's spouse) mind **[Family19_L3]**<br><br>So you know, I can't fault [inaudible 0:12:45]. To me, if it had just been me as a sole person on my own, I would have just come in and gone, do what you need to do. You know what I mean. **[Family19_L3]**<br><br>R: I mean trying to keep in touch with people… My dad's pretty useless with stuff like that. Trying to get in touch with people for certain things. So you don't get a great deal out of him.<br>I: So you feel you have to do quite a bit of that then, do you?<br>R: We've had to, yes. Because my dad's stupidly vague. And I don't mean in the amount of vagueness he has, he is quite stupidly vague, where he'll quite happily turn around and say yeah, I can cope. Unless someone was sat there with him saying no, you couldn't, and then he'd agree to it. So he needs a helping hand in quite a bit, to be fair.**[Family20_L2]**

		I: But no one came to you and said right, we're going to change the medicines to this because of that? Were there any of those conversations around that?<br>R: No. There possibly were the conversations with my father but his memory's not very good. So he's… I mean you ask him questions and he gets things wrong and you then speak to a doctor, it's like no, no, this is what we've said.<br>I: Yeah, precisely.<br>R: Which I always do anyway. I always ask the doctor after something's been said. **[Family20_L2]**<br><br>I: So basically, they're talking to him but then when he's mentioning things you can then intervene, as it were, and… So the next question actually is about decisions, about your family members' medicines. So I mean in what way do you think you're included and involved in any decisions about that?<br>R: In what way? I think…I don't think… Yeah, my dad's mental state is not good.<br>I: Right, yeah.<br>R: Not good at all. And I think judging on how the doctors speak to us and the physio speaks to me and my brother, or my partner, is that they don't leave the full decisions to my dad because he's not capable of making the decisions solely himself. Hence why they like to speak to us as well. I'm still waiting to speak to the physio but I can't get in early enough from work to speak to them at this moment in time. I have to probably make a phone call or something like that. But I think that's the main reason why we'd be so involved in answering questions of any decisions that are made. And I feel myself anyway, knowing what my dad is like, I would like to be rather closely involved in any decisions made. Because at one point they were just going to send him home, discharge him at the beginning of the week, like Monday, Tuesday. But you can't do it. He's not fit to come home. It's not safe.<br>I: No, quite. No, precisely. So do you feel that you are taking an active role in the decision making, to go and see people, to ask questions to say what's happening?<br>R: Yes, I do, yeah.<br>I: Do you feel you have to do that because people aren't coming to you or is it more that you just feel you need to and want to?<br>R: Oh, I need to. I need to, not particularly because people aren't coming to me, it's the fact that I don't trust my dad with the decisions. **[Family20_L2]**<br><br>I: Yeah. Actually, just checking back a little bit, you mentioned that your sister was considering not going back onto antidepressants, have you been involved in that conversation with, either her or with the other medical staff around that, or has that really been her talking to them?<br>R: She did mention it to me, that she'd not had her antidepressants or her vape for a week, and that she'd spoken to the doctors, and she was going to see how she got on without them, and they reassured her that her doctor would have an alternative, after she's discharged, if she gets any low mood to speak up. And so I found that reassuring that she felt she could try without them, especially with everything that she's gone through as well. But that was her decision and I just respect her decision. […] But I will then keep a close eye on her to make sure, 'cause I said, I don't want you going down a path you can't get back from, because she's been there before. But I am well aware of what signs to look for through personal experience with her and other family members, so I respect her decision, I think it's great if she can come off them but be aware that she may need a prod to go to the doctor's if she's not herself. **[Family21_L3]**<br><br>R: Right, well the thing is, because my dad...while my dad was in critical care…well, first of all, he was in taken in to resus by the paramedics and I did actually ask the nurse, they were giving him morphine and they did actually ask me about his medication and I said, because my dad hasn't got dementia, he's fully compos mentis, he's totally in charge of his own medicine. And the nurse said that's no problem because they have it all on a screen, they had it all on the computer what my dad was taking, obviously, because all the computers are linked, aren't they, they know what medication he's on. **[Family23_L2]**

		R: Yeah. And that took several hours it took for me to…and, you know, I'm not one for shouting unless I really have to but I really blew my top, to be honest with you. It got to a point where we'd asked several times, (name) had been so patient. He was, at this point, throwing up, he was in absolute agony. All the progress that he'd made in the ITU had gone. He'd, at one point, asked to switch the machines off and said I don't want to be here, I can't do it, he was in that much agony. And as a mother, to hear your son say that, as you can imagine, that was the point that I blew my top and I demanded to see the consultant. And finally, after hours and hours and hours of waiting, they finally prescribed him…the first thing was paracetamol but then got him something stronger, which was they put him on…I believe was oxycodone which, it was the button that was…and then that started making him sick. **[Family25_L3]**<br>R: To be fair, it was a mixed emotion, if I'm being honest. You know, inside I'm broken because my son is in agony and there is nothing that I can do. I'm angry because I know there's people that can do something. I know that they're stretched and I understand and totally appreciate that, however, I just never felt that they were on our side. So it always felt like a bit of a fight. I had a consultant's words to me, when I…because to me, they weren't checking (name), they weren't…I'm very clued up on healthcare, so I work in the healthcare industry. So, you know, they'd done no pressure care with (name), they'd not come in to check for anything. His feet were overhanging off the bottom of the bed, they'd not offered to take him up the bed. There were so many things going on that it just built up over this time period of (name) being in the state that he was in. I said to the consultant, it's institutional abuse, which it was. And he said, I completely agree. And that to me was just like the icing on the cake because then he said, however, we are short staffed. But, you know, for me, how is that the patient's fault? Not just my son, I don't doubt other patients are probably in the same boat, however, you know, it shouldn't be that way for anybody. So it was such a mixed emotion, to be honest. It's quite heartbreaking as a mother to watch your son in that much pain to the point that you feel like you have to blow your top just to be felt like you've been heard. **[Family25_L3]**
	**Patient willingness - and motivation - to engage**	R: I don't need encouragement. That metformin, as soon as I realised it, I were in [inaudible 00:11:11] straightaway. And if I had any doubts whatsoever what I was being given without explanation, I'd have pulled them. I'm not shy in coming forward, not by a long way. **[Patient01_L2]**<br><br>I: Do you feel that you've been involved in some of these decisions, say you've talked with your mum, and you've talked with the doctors, how do you feel included and involved in what's happening with those?<br>R: Well, everyone has always spoken to me about everything that is happening around me and always made sure that I was aware of it, even in a confused state and that way, I was able to make executive decisions with them, but I wanted them to make them with me because of the confused state, if I'm honest.<br>I: Right, okay, that's really helpful and so you feel that there's been a discussion, you feel that…you said you feel you've been included in all the decision-making, has that been important to you then?<br>R: Yes, I like having a sense of control.<br>I: Okay, so do you think it's important to have a sense of control around your medicines you're taking, is that because of what has happened to you?<br>R: Yes, because it shocked me so much when I got carried away, I'm way more strict on that side of things now. **[Patient06_L2]**<br><br>Well, I came down here on Monday, for a skin graft. Sorry, no, I came down here on the Friday, had the skin graft on the Monday. So now I've got the dressings coming off tomorrow. So when they come off, obviously I'm going to be asking what the situation is, what I need to do dressing-wise. […] And all that sort of thing. I mean, I'm obviously…well I'm saying obviously, [inaudible 0:15:35] but I'm quite rooted now, I'm up and about to a degree, I'm [inaudible 0:15:40]. So all being well, you know, at the end of the week I'm hoping to be…tootling off home, kind of thing. You know what I mean? […] You know, in this next week, there's quite a lot to happen. […] So I'm capable now of asking those questions […] And getting the answers that I need, you know. **[Family19_L3]**

		R: But if ever I wanted to ask a question, if ever I was concerned about something or I wasn't sure what was going on, the staff on critical care were amazing and would always take the time to sit down and go through it all with me again. And even though I couldn't communicate either because I had a trachea in that prevented me from speaking, even to the point where the staff they knew that I was getting frustrated because people couldn’t lip read particularly well, so they got me a board so I could write on it and communicate that way. So, I feel like…yeah, it was a bit strange because obviously choices were out of my hand to begin with, but when I was communicative and once I had questions, people had all the time in the world to explain to me what was happening. So, I feel like I've had as much communication as I possibly could have.<br>**[Patient24_L3]**
	**Preferences around information - necessity of, lay language, level, amount and timing**	R: We’ll leave that to the best doctors, doctors know best.<br>I: Yeah, okay. So, do you think…but you do like to have the information once they’ve done it?<br>R: If they’ve introduced a new medicine or they’ve cut down on some medicines, as long as if there’s a doctor there or the nurse, as long as they inform family members this has happened, because at the moment, I’m also my dad’s next of kin, so they ring me up and tell me or…because my dad’s got…in total, there’s eight kids and it’s…at the beginning, there were too many of us ringing in and asking [inaudible 12:12] and everything. So, we’ve all cut it down just to me now, so I’ll ask the doctor, or the doctor will ring me or the nurses and everything, so no one gets confused in that matter, so it’s just like one person or whoever’s with me and that’s it. **[Family03_L2]**<br><br>R: Well basically when they came to give them me I said, you know, what is it you're giving me? So, they told me what it was and why they were doing it. And I said, well I just want to…you know, I'm not being nosey, I just want to know. And they said, no, it's your body, you're entitled to know what's going into it. So, they did tell me what they were giving me, yes. I can't remember what they were giving me but, yes, they did tell me what they were giving me and why they were giving it to me. **[Patient04_L2]**<br><br>I: Right. So, there is a…I mean, in some respects, some medicines have been stopped, some are going to…have been restarted, but maybe…and have they…in terms of the information that was given to you about both…focusing, I suppose, on those blood pressure tablets and the iron tablets did you…do you feel you, you know…you…what do you…how do you feel about that, the information that was given to you?<br><br>R: Well it was fine because whilst you're…with doing this, and so on. So, maybe I could have had a little bit more but at that particular point I obviously didn’t feel that…well I didn’t feel that I needed…I didn’t feel I needed the information at that point for me to say to them, why? Maybe they could have initiated it and said, we've stopped it for x, y and z. Whereas I…but later on I might have said, well why is it I'm taking that and why I'm…or why am I not taking so and so? At the point that we're talking about I wasn’t really interested whether or not…for what reason I wasn’t taking them, put it that way. **[Patient04_L2]**<br><br>R: It's…the reassurance can be all you require. That is that which comes from a) the individual who's giving this medicine anyway, you want them to know everything about it, you know, and if you ask them a question, or you're lucid enough to ask…to ask them a question, you want them to be able to answer it without having to stumble over words or without having to use chemistry which you don’t understand. You just want the plain, simple English… **[Patient05_L3]**<br><br>Do you think there’s another way that that information that you get in that very small print leaflet in the box, it’s, do you think there’s another way that that could be given to you? Or could you give a perhaps a, do you think there’s some other way people could tell you?<br>R: I thought the doctor could explain it. See you, just a little bit, have a couple of minutes of his time. If he’s going to give it you, new medication or give some, prescribe it or you’re going to get new medication, you should just give a couple of minutes of your time. Just say, look, this is [voices overlap 21:27]. You’re diabetic, you’re this, you’re that, you shouldn’t be taking this or…It’s just, things like that but you don’t get that anymore. You don’t.

		**[Patient07_L2]**<br><br>Yes, and I don't think I probably asked searching questions. It's a very strange thing when a relative is so seriously…and they'd say to me, is there anything you want to ask, and you've almost got…and I almost…as the weeks went by because she was in there for some time, I would almost say, is there something I should be asking? I guess, they don't know how much information to give to relatives so I don't know that I always ask the most pertinent of questions. You know, you're just kind of like surviving day by day really. **[Family11_L3]**<br><br>I: One of the things you've talked through this is saying you're very prepared to ask questions, but do you think the prompt should come from…should it be down to the patient or the family members to say, well, what's happening? Is it actually better if it comes down and they say, what would you like to know or do you know?<br>R: I felt that when I was on ICU that I got the right information. Yes, I think I did because I think emotionally at that point there's probably only so much that you can cope with when you've got somebody you love so much that is in so much…I felt that what they gave me was just about probably what I could absorb. I probably…now, you know, and I guess only because of us having this conversation about my mum saying, oh, what are these tablets I was having this morning, so I guess, maybe, I don't know, maybe more information now. I'm not sure. I don't know really but, no, I think during her time in ICU, I thought the staff had got that right, I do. **[Family11_L3]**<br><br>I: No, right. How do you think patients can be best involved in their decisions about their medicines?<br>R: I think it's quite tricky these days isn't it, because a lot of people have got some knowledge of medicines and I'm very much the exception to the rule, I think I've got quite a lot of knowledge of medicines, and a little knowledge of them is probably a bad thing, stuff looked up on the internet or what have you. I don't know, it's just everyone's different, aren't they? I don't think there's a sort of general rule that you could apply. I suppose what we've got now which is let's go and see this person and see, you know, whether they are able to engage in the decision themselves or whether they are quite happy to let the doctors know best, I don't really know.<br>I: Yes, well, it's almost you say, yes, it's almost like an individual, personal, case by case basis, isn't it.<br>R: Yes, I mean I think most people when they go into hospital they feel as though, like, you know, do whatever they want, these guys know best, you know. But as and when it became a decision for me, if there was certainly any decisions being made for me, I was certainly allowed to make it myself **[Patient12_L2]**<br><br>I: Yeah, yeah. And how do you feel about that (name), that you're the one that's having to do that, that asking?<br>R: In a way, I ask the questions that worries me. If they actually told me what was happening, I think I'd be inundated with knowledge.<br>I: Yes, it would be…<br>R: And I probably would get confused.<br>**[Family17_L2]**<br><br>R: Yeah. I just said, you know, what is happening, is he improving, you know, and everything like that […] Because I think if they gave me all the knowledge they've got, I'd just be swamped with it. **[Family17_L2]**<br><br>I: Yes. That's really interesting actually because this is not something which…it's something I've heard before in some respects. So actually I'd like to explore a bit. In what way does the amount of information you're getting…was it right or was it too much or was there not enough? Or is there a limit to how much you can absorb in some respects?

		R: Yeah, I think the problem is when somebody's in critical care every day is different. I think the nurses did a very good job on making me understand the process of intensive care and the fact that one day he could have a really good day and it seemed very positive but then the next day something could happen it could be not a very good day. But they made me feel very reassured in letting me know that is very common. A lot of the nurses all use the same phrase which is two steps forward, three steps back, and that gave us a bit of reassurance that when he did have a bad day it was just a bad day and tomorrow's a new day sort of thing. So I think that was very helpful. But I think it is a lot of information and I think when he was on maybe six or seven different medications a day on IV drips, he was on dialysis machine, ventilator, he was on a lot of different things, a lot of different medicines, they kept trying different antibiotics. So I think every day there was a lot of information and there was always something that had changed or something new that needed to be added or this needed be up a few milligrams and this needed to be reduced. <u>It is a lot to take in.</u> **[Family18_L3]**<br><br>I: Yeah, precisely. So you talked about hard to find the relevant person. Who for you is the relevant person to talk to?<br>R: Well, I think in intensive care, I was more than happy just to speak to the nurse, because the consultants and the doctors used to meet at lunchtime and they have a bit of a mini MDT meeting, so all the different specialities will discuss the patient and then they will feed back to the nurse like the plan going forward either for the next day or the next couple of days. So what used to happen is, once I got there at tea time I'd know a bit of information, so she would be able to feed back a bit of something to say oh, the doctors aren't happy with this, or the doctors want to review this or he's going to have this scan. You'd get a lot of information. Whereas I feel like at the minute I don't know who's looking after (name) I don't actually know who the named doctor is or who should be reviewing it. And I think because the nurses are that thinly spread on the ward, I don't actually really know the nurse. For instance, a minute ago I just grabbed a lady that walked past and I said oh, I'm just looking to find (name's) blood results for today and she was like oh, I don't have that information, I don't know which nurse it would be. So it's a bit like that. I think it wouldn't be as frustrating if you'd not been in the intensive care environment, but I think because you are used to that intensiveness and that information overload sort of thing and you get to know a lot of stuff, I think it is a big massive change from intensive care to the ward **[Family18_L3]**<br><br>I: In what ways would it have been good to have been involved?<br>R: Well, it's just nice to know what you're taking, what it's for, and why you're not taking what you were taking before. You know, okay, yeah, we understand that you were on an antidepressant before you came in, but this is why we've stopped it, do you think that you might need something to replace it? How has your mood been, is it any better?<br>I: Yeah, absolutely, those sorts of questions then would have helped, would they?<br>R: Yeah.<br>I: How do you think patients can be best involved?<br><u>R: Talk to us, just talk…</u> **[Patient22_L3]**<br><br>And because, why I obviously seek and ask these questions. But I think, from a standpoint of, with my father, when he ended up coming with me, a lot of the information sometimes would have gone over his head, in regards of, it wasn't quite explained in a way where, if you didn't have even a little bit of knowledge beforehand, it could be something that doesn't quite meet the expectations of somebody who wouldn't quite understand. Or, like, if you didn't have any understanding of, like, what these things were, sometimes it did, it was, like a point where my dad wouldn't understand why she was on it. And it was very much, you'd have to give an explanation. Maybe if there's somebody else, obviously, in my place, and stuff, I just feel that, maybe a little bit more information would be good. […] But I think it's very much difficult to do presume, obviously, because it was me who was there, I don't really know. But from my father's perspective, a lot of it I'd have to relay to him, or at least reassure him about, if that makes sense at all. **[Family29_L3]**

		R: When you're, like, yeah, when you have, kind of like, a basic understanding at least, it's very different to somebody who's probably never even studied anything like it.<br>I: Yeah, precisely, yeah, it's a difficult one, isn't it?<br>R: So, with my dad not really knowing, it's kind of like, whether this information was okay for me, but whether it was okay for my dad, is kind of like, a different story.<br>I: Yeah. And I suppose, were you then given, or he, given an opportunity to say, oh sorry I don't get that, can you go through that again, you know, was there any of that?<br>R: I think there was, like, the opportunity. I think it's more the reliance of my dad for me to explain it, if that makes sense. […] Like, there would be an assumption that I'd probably explain a bit more later. But the opportunity was there, as I say, like honestly, I could ask anything, I could probably seek any information, if there was nothing, or like, something that, or we weren't able to, like, understand. […] The door was always open, if that makes sense, like there was no pressure, and it was always, we never felt like you couldn't seek any help, or any extra advice, or any extra information. **[Family29_L3]**
	**Trust (or lack of trust) in health professionals**	R: Yeah. I honestly, I think if I'd have turned round and said I don't want the tramadol, you know, the effects that it has, like, and they are saying it's the…advising me it's the best they can do, but I certainly feel if I'd have said I don't want it, they wouldn't have, like, tried to railroad me, they'd have given me something else.<br>I: Yeah. Right.<br>R: I was quite prepared to accept their professionalism and take it, you know, and get it, sort of, this [once, like, you know 00:12:16] and get there. **[Patient01_L2]**<br><br>but, no, you soon find out whether or not you trust. I think that trust is the most important thing.<br>I: Right, it's about trust then, trusting the person talking to you knows what they're talking about, is that what you're saying?<br>R: It's…well it's certainly the front person, the person who's issuing this stuff, you know? I mean say it's…you would expect someone who's doing pharmaceutical round the ward in ICU to have knowledge of what they're talking about because they've talked about it so often and the doctor would have talked to patients and shown them what the results are and why they might be used. And once you've got that from them then everything else, you know, it is trust. You know, if you lose the trust you've lost everything. **[Patient05_L3]**<br><br>R: Haven't got a problem, I trust the people.<br>I: Okay, you trust them, right, okay. So what is giving you that trust?<br>R: I don't know enough about medicine. Up until, excuse me, [voices overlap 00:11:22] up until this happened, I would take a paracetamol if my, if there was a little bit of a pain, and Ibuprofen for my bad back. And that would be it, yeah, and then suddenly I've got this major problem, major, I was told it was life-saving surgery. I don't anything about it, I didn't even know what had happened to me and suddenly people are there, and I've got to trust them. **[Patient08_L2]**<br><br>R: You know, I'm an intelligent guy I, you know, but I'm no medical person, I'm 71, you know, I can go to Boots and buy paracetamol, Ibuprofen, can go to the Boots Pharmacy and say, look, I'm feeling a bit like this, what would you suggest? But anything beyond that I have no knowledge whatsoever, and yet I'm sat there in my bed in front of a consultant who's got 30 years' experience, you know, why shouldn't he decide for me? **[Patient08_L2]**<br><br>I: Right, so, yes, I mean, this is really interesting because it's, sort of like, who's aware of what's going on as it were and how much you're involved…<br>R: In that process.<br>I: …included in that process? Yes.

		R: I guess, I'm probably very old fashioned in thinking they're a professional, let them get on with it rather than me asking any questions in any way. I guess that's kind of where my…yes.I: I mean, do you feel you would like to be more included, involved in the…?<br>R: I don't think so, no. I'm more very vocal if I can…the physical wellbeing and all that, I would be very vocal if I thought that my mum wasn't happy and I guess, on the medical side because I've got no medicine knowledge at all that I just put my trust in them but I do feel that if I did ask the questions, then I would get the answers. I feel confident enough that I would ask either the doctors or the nurses so I don't feel inhibited in that way. I think I'd probably just blindly trust them with the medication. I think that's where that's coming from **[Family11_L3]**<br><br>Well really just as someone with no experience in medicines, like you said before, I wouldn't know a lot of medications. I suppose you put a lot of trust in people that know what they're doing, don't you? So you don't really feel like you need to ask because you think they know exactly what they're doing. […] I suppose as long as there have not been any problems or anything, there's no real need for me, how I feel about it, to really ask that much about stuff like that, I suppose. **[Family15_L3]**<br><br>Well, I didn't know, I trust them, you know, it's as simple as that. Because like I said, we don't really have much to do with the medical side of it […] So, I just trust them, and if they're doing something, I'll say, why are you doing that, and they'll explain. But they've never discussed why they're giving him anything, or anything […] They just put him on it, and then we ask, what's that he's having, and then they tell us.<br>I: Yeah, absolutely, yeah. So, do you think that trusting, do you think that trust is important, then, is it, you know?<br>R: Well, I don't know anything about medical, so I have to trust them. **[Family17_L2]**<br><br>I don't think I have been involved in any of the decision making, but that doesn't bother me. She's got her husband, and she's got her two sons, although I kind of have taken the lead a lot of the time, through them asking me to. I think we've just allowed Critical Care to get on with making her better in whatever way they need to. […] And it was having that trust in Critical Care. I'm very experienced with (name of hospital) and it's a great hospital, the staff are fantastic. So we've just trusted that they've made the right decisions for (name of patient) when she was unable to do so. So I've not been too concerned that I haven't been consulted, they're the professionals, I don't like people telling me how to do my job, so I let them get on with their job. **[Family21_L3]**<br><br>I: Who's been making, or who's been involved in that decision making, has that been, you know, yourself or your dad or a combination of that, or the health professionals, or what?<br>R: Well I think that would have been the medical team, that would have been the medical team because they have to juggle a lot of things, don't they, in critical care. So, you know, you can't start telling a consultant, oh, I want him to be taking this medicine when that could kill him, [voices overlap 00:17:36]. If he was on a painkiller that, if you mix it with another medication that he normally takes, could kill him, you know, just, I'm saying, I'm not, I don't know that, they know the medications and the type of things that you can take together and that you can't take together, and things like that. So, to a certain extent, you have to leave that to the medical professionals don't you ….**[Family23_L2]**<br><br>I: Do you feel that when you've made that, do you feel you've been supported by what you…your choices within that?<br>R: Yeah, I would say that as well I am very conscious of that fact that I'm not medically trained and that these guys know a lot more than I do. So, as long a somebody explains something to me and it makes sense in my head, then I'm happy to go with their recommendation because they're the professionals, I put my trust them, and nobody's seen me wrong yet. I know I've said it's my choice and it does feel like that, but also I'm happy to be led by a medical professional because they know a lot more than **[Patient24_L3]**

**Theme 4**	**Initial Theme - Care continuity and opportunities for information exchange**<br><br>**Final Theme - Care continuity and individualised information exchange**	
	**Sub Theme**	**Quotations**
	**Differences between ICU and ward**	Well, the doctors and nurses have been really brilliant up in the critical unit, and I think once they're in the ward, it's totally different. You see more busy, in and out and whatnot and it's more crowded and it's totally different. My dad was getting brilliant care in the critical care unit, but then once he got into the ward, he was getting more confused, [inaudible 16:01] asking what he would like, and then there's another person coming, and so, his dinner got messed up and everything because too many people. My dad does get confused a lot, so it was that we were there we asked for what meal to have or he did say, it's confusing, too many people coming asking me things and everything. Because the critical care unit was one nurse and one domestic person, yeah. So, it was confusing for him in the ward. **[Family03_L2]**<br><br>I: Yes, absolutely. How do you feel about the way the information was given to start with? Do you think it was good or could be better or…?<br>R: Could be better, I imagine it could be better because, say, the pharmacist came down and told me what medication I was taking going into hospital and why I wasn't taking it there, but nothing was said as why I hadn't been taking it when I went onto the ward.<br>I: Yes, so I'm with you, so when you left intensive care, no one said, oh, why you're not now on it now, but you said you have had information from the nurses around the anti-sickness medicines?<br>R: Yes, yesterday [inaudible 05:08].<br>I: Yes, right. And do you think…. is it clear, is it understandable, the information?<br>R: Again, a little vague, some of the questions that could…I don't know whether they were reluctant to answer or they couldn't answer. **[Patient10_L2]**<br><br>I: One of the things you've talked through this is saying you're very prepared to ask questions, but do you think the prompt should come from…should it be down to the patient or the family members to say, well, what's happening? Is it actually better if it comes down and they say, what would you like to know or do you know?<br>R: I felt that when I was on ICU that I got the right information. Yes, I think I did because I think emotionally at that point there's probably only so much that you can cope with when you've got somebody you love so much that is in so much…I felt that what they gave me was just about probably what I could absorb. I probably…now, you know, and I guess only because of us having this conversation about my mum saying, oh, what are these tablets I was having this morning, so I guess, maybe, I don't know, maybe more information now. I'm not sure. I don't know really but, no, I think during her time in ICU, I thought the staff had got that right, I do. **[Family11_L3]**<br><br>R: I don't think so apart from just…like especially in Critical Care, the nurses were brilliant. Everything, like their bedside manner and stuff like that. Do you know?<br>I: Yeah, I know what you mean.<br>R: Really helpful, and they'd always say hello and ask if we needed anything. They're really all-rounders. As well as getting on with what they're supposed to be doing with their job, they were really polite and friendly.<br>I: And that helps, does it?<br>R: Yeah, I'd say so, definitely **[Family15_L3]**

R: I think it was from intensive care, to be honest. I think obviously all the nurses on intensive care did say, because when you're in intensive care you're having one to one nursing, so it seems when you do go to the ward because they're all spread that thin you don't…obviously it's not intensive caring, is it […] So they're reviewing status of him, his stats and stuff like that is very scarce now compared to what it was. But I think that's a little bit hard to get your head around from my point of view. I think when you have been in intensive care and you've been looking at the stats monitor constantly and you get a lot of information I think when you do get transferred to the ward you don't get that information and you have to sort of grapple to try and get some sort of information or what the plan is or where do we go from here sort of thing. Whereas when you're in intensive care, literally the nurse will know everything inside out about that patient what she's looking after that day. That for me is more hard to adjust to from intensive care to the ward, even in regards to medicine. If you ask oh, how long is he going to be on this for or what's this one and they'll just say oh, they're just the antibiotics that he has every day. Whereas in intensive care you get quite a bit more information as to he's on this many milligrams and he'll be having this bag which will last this long and he has it at this time and this time. Whereas I think on the ward it's a bit more like oh, it's on his sheet that's what he's having sort of thing.

I: Yeah, right, I'm with you. So there's a more detailed explanation in critical care than there is on the ward.

R: Yeah. I think you get a lot more information as to what's going on, what the plan is, and it's a bit more clearer. I think on the ward it's a bit scarce. Which is obviously understandable because, obviously, you know, in intensive care, it's one to one, it's not like that on the ward. There might be one nurse looking after eight patients at a time.

I: From your experiences of the last few weeks generally how do you think family members can be best involved?

R: I think on reflection, I think…I did express this to the nurses, I think for me as the partner having very strict visiting times, I think that's not very good for someone who is in intensive care just because I feel like every hour something can change, and I think if your loved one is having a bad morning, for instance, and you have to leave them because you haven't got a visit booked, I think the best way to keep informed is to keep that conversation…to keep being around, to keep being involved. But I think having a direct line to the room is a really positive thing. You know, you can ring up in the night and they'll tell you how your loved one is and the information that you want to find out.

But yeah, I think, throughout my experience, our experience as a family, I feel like they have done an amazing job, and I do feel like we have been informed a lot. I couldn't praise the intensive care staff any more if I tried, to be honest. The nurses have done an amazing job. They're really polite, pleasant. I think it's just been a big shock moving to the ward from that environment. And again it's not a problem. […] But then, I think when you're on the ward and you don't see a nurse for quite a while and they're really busy and there's a lot going on, it's quite hustle and bustle, I think that then does give you a bit of an uneasy feeling. I think for the first days especially, when they do get taken from an intensive care environment, if they're not still 100 per cent, obviously which they're not going to be, I think it is a bit uneasy for the family. **[Family18_L3]**

R: Yeah. And I think when you've been that statistically driven and looking at the stats constantly *(in ICU)* and then just to not have them at all it is a bit… And then like obviously in intensive care they were just there to hand, you could just see it, whereas now you can't see the information from the morning, you have to wait until you can grab a nurse to ask oh, what was his blood pressure like this morning and what was his temperature and stuff like that. So I think for me that's been the biggest adjustment. **[Family18_L3]**

I: Yeah, precisely. So it's more of a welfare thing at the moment, you're getting the IV, you're getting the medicines, you're getting better, but perhaps not that same sort of one to one as it were. Have there been any problems with his medicines at all, particularly in this change from critical care down to the ward, that you're aware of?

		R: No, I don't think there's been any problems. I think the only slight problem that there was is because he's on a reducing dose of clonidine, the second day I think he needed the reduced dose, he's supposed to have two in the morning, one in the afternoon and one in the evening, and that's been the schedule, but they didn't have any and they had to order it in from the pharmacy, so that resulted in his second dose being, I think, six hours late. Whereas in the intensive care, they have their own pharmacy on the ward so obviously that doesn't happen. But apart from that, that's been the only little hiccup.<br>I: Yeah. As I understand, again, similar ratio in terms of pharmacy care. Pharmacists down on the wards will be looking after dozens of patients, won't they, as opposed to…<br>R: Yeah, that's it. And they have to order it in, rather than in intensive care, they can literally just walk down the hall and go and get it out the cupboard. **[Family18_L3]**<br><br>I: So, you feel very much involved in the decision-making process there and in those…?<br>R: Definitely. And especially from coming from critical care to the normal unit, where…well, you probably know in critical care, they don't want you to do anything for yourself, everything wants to be measured in and out type thing, they want to see everything. And here I feel like they're very much about that step between critical care and getting home. So, they let you do as much as you want to do by yourself or as much as you can do, and then if you need help with things, then they will help you as well. But it's just dealing with everything as well as the medications, so it very much feels like…almost like rehabilitation in a way, just that step towards getting home. **[Patient24_L3]**<br><br>The pain relief…in terms of like, pain relief and medication on that side of things. We had a bit of an issue when he first went from the ITU down to the ward, where (name) had no pain relief, they'd not had the doctors re-prescribe it from the different system. So (name) went almost…I think it was about 15 hours without anything and he was in agony, absolute agony. And when we were asking and asking and asking regarding where is his medication and why has this occurred, all we got was, I don't know **[Family25_L3]**<br><br>R: Yes. So I think the way that it was explained is that they have a different system in the ITU. So once (name) was moved from one ward to the other, it all needed re-prescribing onto this new system, but it hadn't been done before we left the ITU. However, nobody communicated that with us and I'm assuming between themselves as well, until (name)'s pain relief had ran out from the one that he currently went down with. And it was only when (name) started to experience pain and we asked what pain relief had he got and they said he'd not been prescribed any. So they were unable to give him anything, they couldn't even give him paracetamol.<br>I: Right. Oh, because nothing had actually been put on the system as prescribed? […]<br>R: Yeah. And that took several hours it took for me to…and, you know, I'm not one for shouting unless I really have to but I really blew my top, to be honest with you. **[Family25_L3]**<br><br>I think the main part of it all really, I just wish the communication and the quality of everything between the ITU and, you know, down to the ward, that level of care shouldn't change. I really want that highlighting, it shouldn't. I know they can't give one-to-one care and I totally respect that, working in the healthcare industry I know that first-hand. But that quality should never drop, it should always be top quality when they're with that patient. And half the time, you know, you feel as though they don't even know the person that they're treating. And that's the scariest part of it, especially as a relative, to watch that happen. **[Family25_L3]**

		On the general ward, I believe it's like, you, kind of, seek out, if that makes sense. […] You have to actively seek out and ask. Because I know, I am aware, with it being a general ward, it's not to, the only way I can really put it is, a bit more hands off. Or you end up, there's specific times that people do come to check in on you and give you the medication. But otherwise, a lot of it is down to the patient, and obviously, the patient's family, like me, to go and ask questions, and ask for things like, with my mum's stoma at the moment, I have obviously raised that she is having a few issues with it, like leakages. And due to the nature of her skin at the moment, the adhesive isn't sticking quite right. And a lot of it is having to just know when to go and ask a nurse about it. And in terms of medication, it's very much, as I just brought up just a moment ago, it's at set times, it's very much […] yeah, it's very much like, a lot more structured in the regard of, like, taking account of, there is several more people that the nurses are between. So, with critical care, as you'll obviously know, you have a nurse per person. But when it's a general ward, they have to keep that kind of structure, I've noticed. Because they are looking after about eight, ten different patients, so I do understand …. **[Family29_L3]**
	**Patient (or F&F) feels that there is regular contact from health professionals and opportunities for discussions to occur – or questions to be asked – or not.**	I: Yes, so that's…that that will be the plan. So, you're aware of that and again, presumably the doctors and nurses have been talking that through with you have they?<br>R: Yes, they've talked me through that.<br>I: Yes, in terms of the decisions and about your medicines, do you feel you're involved in those decisions?<br>R: Yes, because was it a couple of days ago I had an antibiotic drip and I didn't know if I had a reaction to it, because I was, like, just laid on the bed sweating and felt really ill for about six hours. And then nurse came round to give me another one and I did say to her about it and she said, oh okay then, we'll contact the doctor and the doctor came and had a word and checked before I could take another one. **[Patient02_L2]**<br><br>R: [Yeah, the daily 12:35] information when we go there, if the nurses are there, just give us a quick chat on what's changed or [inaudible 12:44] things like that and how he's going. Which they do daily, they do, even if you don't ask them, they come down and they'll just tell us, or I'll ask them. This is a daily thing, yeah. **[Family03_L2]**<br><br>R: Well basically when they came to give them me I said, you know, what is it you're giving me? So, they told me what it was and why they were doing it. And I said, well I just want to…you know, I'm not being nosey, I just want to know. And they said, no, it's your body, you're entitled to know what's going into it. So, they did tell me what they were giving me, yes. I can't remember what they were giving me but, yes, they did tell me what they were giving me and why they were giving it to me. **[Patient04_L2]**<br><br>R: This time with the, it seems to be better than I was in before. Because the doctors come round in the morning and they'll come and they'll actually talk to you. And they'll talk to you and say, well, this is what we want to do. We want you out by this much warning. See, this, a doctor might then want to see this. I didn't have this before. If that makes any sense?<br>I: Yeah, yeah. And what do you feel about that? Do you think that's good?<br>R: That. Yeah, yeah, it is good, yeah. It seems to be better. It gives you the confidence because, well, hold on a minute, they know what they're going to do. They've started to bring me into the conversation with themselves and that's it. **[Patient07_L2]**<br><br>And there was also a conversation, a three-way conversation, no…a four way conversation between myself, the upper GI support nurse, the junior doctor and the consultant on the day I was due to go out, and it was a question as to whether I…as far as the consultant was concerned, I was medically fit to discharge. The support nurse, I think, thought that I should stay another night and try and get this oedema sorted out a bit more and be happier before I went home. I wish I'd taken his advice, but in the end I went with the consultant saying, you know, there's no real reason for you to be here and the rest as they say is history. I wished I had stayed because I think the junior doctor was talking about, you know, putting a drip up and getting some diuretics into me, even there and then, but that's history and 28 hours in (name of hospital) is not much fun I can tell you, especially when you spend nine hours lying on a trolley in A&E.

		**[Patient12_L2]**<br><br>But he had to have a review from cardiology which he had this morning, so now it's just waiting to find out what the doctor thinks, because they did mention to my partner when I wasn't there that they don't feel it necessary for him to be an inpatient basically taking up a bed just to have IV antibiotics and he could have that as an outpatient. So obviously that was this morning when he spoke to the cardiologist. A doctor's still not been round now and it's four o'clock sort of thing. So he's desperate to go home. But it's just hard to find who is actually the relevant person. Plus obviously, with all due respect, there is junior doctors' strikes at the minute. So, like I said to my partner, they're spread thin at the minute and you just have to be patient, sort of thing, which he's obviously in agreement, but I think that's not helping the reflection of the information process at the minute because for all I know, you might get a bit more information. Because I think the consultant who works on the ward that my partner's on, is currently away and then the other second consultant has had to go to (another hospital in same trust) to help out over there. **[Family18_L3]**<br><br>R: Pretty much constantly I had a critical-care nurse, so dedicated one-on-one nursing. Sometimes the doctor would come in and say, are you in any pain; and you'd go, oh, yeah, it's quite…right bad at the moment. And they'd talk about what was the best thing to give me, between themselves, and then say, we're going to give you a shot. But I didn't think to remember what they were going to give me, do you know what I mean?<br>I: No. Quite. No.<br>R: At that point you're just kind of thinking, no, just give me something to take pain away, you know. **[Family19_L3]**<br><br>I: And when you've been having these conversations with people, you said about the nurses there, and the Nursing Sister, have you initiated anything, or have they come to you?<br>R: I think it was a bit of both really. They've come to us at times, and then if I've seen the nurse sat at the desk and I've got a question, I would approach them.<br>I: Yeah, so, and have you been happy to do that, do you feel confident to do that?<br>R: Yeah. I'm happy to do that, I've had a lot of involvement with hospitals with our mum over the years, and my dad, so yeah, I'm quite happy to approach any member of staff and say, do you know what's happening, or, can you answer me this question?<br>I: And do they answer the questions, do they answer them…?<br>R: Generally, they do their best to answer the questions. Yeah, they've been great. **[Family21_L3]**<br><br>R: Yeah, definitely, it is good to talk to people to say, well why are you giving her antibiotics? And they've said, oh, we think she's got a chest infection, we've sent off something for testing, but we're treating her for this in the meantime. So, you know, whenever a nurse came and did something, you know, gave her an injection in her tummy, I was like, well what's that for, and they would explain what it was for. And then I just think, well yeah, that's fine. There was no one time where I thought, actually, that makes me feel uneasy, I don't want you to do that. There was never that time.<br>I: Yeah, that's interesting isn't it? 'Cause you're getting the opportunity to ask questions. And when you ask those questions, do you find the response, well what's the response like?<br>R: Oh it's always been very good they've always took time to explain what they're doing, even like when they were taking blood, I was like, well why are you taking blood from her, what are you checking for, and they would explain what they were checking for. So yeah, the explanations have always been really good. **[Family21_L3]**

R: Yeah, I’ve not actually sat down and had a conversation with anybody regarding medication, because before I came in to hospital before I had my arrest, I was on Duloxetine, on an antidepressant of 60 mg a day, and I’ve been asking for the last two days where they are, and apparently, they’ve been stopped. We’ve only found that out because (name of nurse) asked them today for me.
I: Right, okay. So you’ve had medicines beforehand that you’re on, this antidepressant…
R: Yeah.
I: …and that’s been stopped you said, but no one’s come to you and talked to you and discussed with you about why that’s been stopped, have they? **[Patient22_L3]**

R: I asked a couple of times what he was being given, painkillers, and I think that Tramadol was mentioned at one point and, oh, he was given something to stop his lungs from, I don’t know all the medical names for all these things, well he was given something to stop his lungs from filling up with fluid. He was given antibiotics, yes, it was all explained to me as they were giving it, you know. When we came, [inaudible 00:05:00] was, sort of, in the evening, the consultant saw us twice and the nurses gave us all the information we ever asked for, you know. So we were all aware of what he was, that he was getting his full medication. And, like I say, when he was transferred onto the ward he was fully compos mentis anyway, he was sat up and talking to the nurses, so he knew. And, like I say, I did ask him last night, was he aware that he was getting all the medication that he was getting before the accident, and he said, yes, they were giving him all the medication that he should have been on.**[Family23_L2]**

R: ...I was going to say, on a couple of occasions they mentioned something that I didn’t understand, you know, they mentioned a name of the medication. And I said, well what does that do? And they explained it, that was, well we’re giving him that because of this. And even down to his oxygen, he had to go in, like an oxygen thing, like a bubble really, which he hated. And they explained to me, it was like a CPAP thing, and they explained to me why he had to be in that, because the oxygen levels, they were pumping the oxygen levels into his lungs. And then, gradually, as his lungs improved they took him off that and put him on different types of oxygen, and all that was explained as well. Because, you know, obviously, when you see my dad in, like, a plastic bubble, you want to know why he’s in there. And it was all explained why, why he had to be there. And, you know, every question we asked they answered, if I wasn’t sure if he was being given a medication for something that the consultant had mentioned, and I said, well what was that for? The critical care nurse explained it, what it was for.
[Family23_L2]

R: Yeah, it’s just the nursing staff that administer the medications. Obviously they knew what I was on when I came from critical care and they just, is that working for you? Are you happy with that? Do you want this? Do you want that? So, they asked me every single time, it’s not a, here’s your medication you must have it. We progressed it a little bit in that speech and language therapists weren’t completely happy with my swallow, so rather than trying to swallow tablets now, I get like a…what do they call them? Like an effervescent tablet, isn’t it? […] So, everything's liquid form now. But, yeah, it feels like a choice, like it’s a, you must take this. This is what it is…I also have an injection in my tummy once a day as well, I think that’s a blood thinner because I’m not moving around as much. So, that’s an option. There’s also an option to have…oh what do you call them when you have the face masks with the…a ventilator, essentially like an inhaler. That's also my decision but they always try and give it me when I've got visitors, so I didn’t have that for the first few days. But that was to clear stuff off my chest and every day I feel my voice is stronger. So I had this conversation with the nurses and I think they all tend to agree with me and we’ll kind of like come to an agreement that it's not really necessary. **[Patient24_L3]**

I: Did you initiate some of those conversations, you said you asked questions, did you start off the conversations, or did they, you know, come to you?

		R: You see, sometimes it was a mixture. Because a lot of the time, I do, kind of, seek out answers, as and when, that's just the nature of me, I think. But, even when not prompted, they would at least give me a general overview of the medications she was on, and any progress that was made within that day, when I'd arrived. It was just, sometimes, I ended up going to ask myself, I think that's just, as I say, I think that's just me as well […] Because I think, from what I gathered, when I was going to the critical care unit, they would have just let me know anyway. It was just, sometimes, if I had a question, anyway, I'd just go up to them and ask, yeah, before they got to me, really. **[Family29_L3]**
	**Quality of conversations, treated with respect, reassurance, empathy, clarity, etc**	Probably as much me not being able to focus. I think the problem is, you're told stuff, and if I go back in, like, my work situation, when I was working, if my gaffer came round and gave me an instruction, I'd get it written and I'd know what to do, and then I would look at it, and go, and this is wrong, and you'd go back to him and [inaudible 00:07:57]. But because it's all verbal. I know them pamphlets they get, can be a pain, but maybe if they had an iPad where they could pull up just certain areas what they want to emphasise to you. But the guy was very, very clear. And he spent time with me, he didn't just come in, bang, bang, bang, bang, bang, bang, make just ten bullet points and go out. He came and he took his time with me. **[Patient01_L2]**<br><br>It's…the reassurance can be all you require. That is that which comes from a) the individual who's giving this medicine anyway, you want them to know everything about it, you know, and if you ask them a question, or you're lucid enough to ask…to ask them a question, you want them to be able to answer it without having to stumble over words or without having to use chemistry which you don't understand. You just want the plain, simple English, but I'm sure you know that anyway because you're doing the study. **[Patient05_L3]**<br><br>I: Right, okay. Do you think, has there been anybody in hospital who's talked to you about them? This plan?<br>R: The hospital, I've got to admit, they've done alright, they've done, they've tried to explain it to me, though, in the way that they see fit. But sometimes when they try to explain to you, it seems like they're talking to you, like you're a little child.<br>I: Oh, right. Okay. What does that make you [voices overlap 11:07], yeah, what does that make you feel?<br>R: It upsets you, it gets you angry at first. Then you just, you think to yourself, they must think, I just think, why are they talking to you like this? Then you just talk to the wife. The wife settles you down and that's how it was in my family. I over-exaggerate. I get angry and then the wife calms me down. **[Patient07_L2]**<br><br>I: Do you feel these explanations, if you've had explanations about medicines, they've been, as you say, you've been talked to as if you're a child. So, how would you like to be talked to? What would you like to be told when you're given these explanations?<br>R: Just, like, oh, okay,(name) your medicine, now we've changed it. We changed it because we feel that in hospital, right now, you're not eating as much. At home you'd be eating as much, you're not having as much sugar breaking down or these medicines that you're having now, you need to have more. More of these water tablets because it's not working for you or just, do you know, explaining, not just do it. So, they'll just change it without explaining it, yeah. You know when they try to explain it to you, they do it fast. It's really fast. It's confusing.<br>**[Patient07_L2]**<br><br>R: Well, very kind, very empathetic. The doctors, the consultants when they do their rounds are very presumptory you know, they don't say a lot. You sometimes have to, you know, you've thought of a question and they've gone before you get to ask it. And then that's ward rounds, isn't it? But I've had two sessions today, one with the physios, who were brilliant, brutal but brilliant. And one with the, the stoma nurse who was very empathetic, she was lovely, she reassured me<br>actually. **[Patient08_L2]**<br><br>I: Do you think that's how family members can be involved or perhaps are best involved in that sort of way or perhaps to rephrase that how do you think family members can be best involved in these sorts of decisions? Do you think, as you say, leave it to the professionals?

R: Maybe relatives should be asked, are you aware of what medication your mum is on? Would you like to know? Though I would feel confident asking, not everybody would, would they, and not everybody would know that it is their right. Actually, to be honest, if I had been asked that question, I would say, yes, please. It's just not occurred to me. Had we not been having this conversation it would just not occur to me to kind of question, kind of like, where are we, what are we having? Yes, I think it would be really useful and good practice to ask an open question like that, you know, saying, are you aware? Would you like to know? **[Family11_L3]**

R: I guess, as a relative, I think the personal care that…I think I just take for granted that higher up the doctors know what they're doing, they know what they're doing with the medical care but for me, it's the personal care that my mum's being washed daily, that they're taking time with her hair, that they're talking to her as she's being given the medicines. That was quite interesting, which staff, even when my mum was really heavily sedated which staff when they were putting things in her line were actually talking to her and saying, (patient's name), I'm just going to do this now, (patient's name), I'm just doing that now, I'm just going to do this now, (patient's name), I'm just going to do blah, blah, blah, that was really important to me and not every member of staff did that. Actually it's really reassuring as a relative to actually know, this is what I'm doing now, this is why I'm…do you know. **[Family11_L3]**

I: Right, okay. So, how were those changes explained to you?
R: Well, it only sort of became apparent to me some way into the event that the normal blood pressure medications, including the diuretic that I was taking, they all stopped doing operate…I didn't know that at all...that they were stopped. And it was when I kind of realised that I was blowing up a bit like the Michelin man that I kind of had the discussion with one of the doctors I think it was about, you know, whether that had changed. I'm sure…but yes, it's definitely been discontinued, you will go back on them, but it will be a gradual process, and I'm thinking well, the rate this thing is going, you know, I won't be able to get out of the bed, you know, I'll be a barrage balloon […] But I think they thought better of that and then eventually decided to restart my normal water tablet, which is a fairly low dose, I think, and I was sent home on that. And we had…I was on Lisinopril, 10 milligrams,
Amitriptyline, 25 milligrams, and Spironolactone, 12 and a half milligrams. And so they restarted the Spironolactone when they realised I was getting a bit oedematous and then said let's start taking half of the dose of Atenolol, because I was tacky. (Tachycardia) […] Leave the Lisinopril, so there was kind of like a…then there was a schedule of when the various tablets should be reintroduced by the GP and it was over to you guys, you know, in terms of the GP sorting the oedema and the whole blood pressure thing out again. So, that was when we were sent home and that was kind of semi explained to me and semi negotiated by me really. **[Patient12_L2]**

And they were brilliant, they explained everything, how everything had gone. Yeah, I can't honestly remember all the specific details as to what… With regards to what medicines she was on and stuff like that. I know she was on a PICC line and she'd been on that before her surgery, before Critical Care, she was still on that for a while after Critical Care because she couldn't eat, still. That got explained to us again. I mean, we already knew but they took the time to explain it all to us again. Yeah, they were really good. **[Family15_L3]**

And…so the transfer was…which is what you're asking about, was…well it was seamless, I'd say. I couldn't really find fault. If I'm really looking to find fault, the only thing I could say is that the lansoprazole was put in and I didn't know it was put it until I saw it on the ward. I presume it was started in the HDU, but I wasn't aware of it, because by the time I'd gone in in the morning, 'cause you're not allowed to go in the HDU until 11:00, whereas in the normal ward I was going in at 8:00 so I could see her morning medication, you see. And I could see it where of course I couldn't see it before. It was a perfectly sensible thing to put her on and…I was just slightly surprised because I hadn't been told. So I asked what I was and they told me straightaway. The nurse knew what it was. She didn't have to go away and consult a chart or anything.
[Family16_L2]

		R: So basically what would happen, my partner would get started on different variations of medication. He had quite a nasty infection and the cultures had not been grown from the lab. They weren't too sure actually what the infection was. So when I get there in the morning, there'd just be different antibiotics, names and different bottles and stuff. So I was quite inquisitive because I do work in the NHS myself<br>I: Oh right, okay. That possibly makes a difference.<br>R: I'm not clinical but I do work in a clinical environment so I do have a bit of an understanding, so it made it a lot easier for me. But I think like my partner's mum, for instance, who doesn't have any medical terminology or anything, she found it really hard to understand what all the medicines were and what they were for. But the staff in the intensive care unit were very, very helpful and tried to explain, like this is for blood pressure, this is for this. So they were really good in explaining stuff. **[Family18_L3]**<br><br>I: And what were those conversations like? Were they useful or helpful or…?<br>R: I think they were helpful. I think obviously in my own experience working in that environment, I think nurses in general are a bit better at explaining things in layman's terms rather than doctors and consultants who use big words and stuff. But yeah, obviously at that time when things are quite up in the air, it's a lot to take in anyway, isn't it, for anyone. But I think the nurses did really do a good job in explaining what was going on in regards to medicine**. [Family18_L3]**<br><br>R: Yeah. I feel like on reflection the intensive care nurses do an amazing job. They don't reassure you in the way, they don't say your loved one is going to be fine, because they are open and honest and they will tell you that they can't give you that reassurance, despite how desperate you may be for it. They don't give you any false pretences that your loved one is going to wake up tomorrow and they're going to be fine, because they can't tell you that information, which I understood from the beginning. But I think they reassure you in the way when, like, for instance, he was put on a ventilator and for me that was the worst thing that could happen, just being put in an induced coma, I thought that was meaning he's not going to wake up again sort of thing. I didn't understand the actual process of it. Because you only hear about your friend's aunty who was on a life support machine and they had to turn it off because she was unresponsive. You don't hear about the amazing things that the intensive care people actually do to save lives. So I think when they sat us down and explained the process of what would happen to his body and what medications they'd be using and stuff like that, it did make me feel quite reassured that it was a positive thing as opposed to a negative.**[Family18_L3]**<br><br><br>R: So yeah. So probably about four times a day I get a pot full of tablets.<br>I: Right. Okay. R: So there's paracetamol, gabapentin, omeprazole-type…one of them, lansoprazole. Let me think, a couple of different types of blood pressure tablets, and no doubt there was a statin in there of some sort.<br>I: Yeah. Right. Okay. And again, in terms of you say four times a day you get a pot load of medicines; when they bring those along to you does someone talk you through that, or is it just here's your medicines, (name) , and, you know, crack on, as it were? Or is it a conversation about, all right, we're changing that, or doing that, whatever?<br>R: The majority of time it's literally, here's your tablets, and there you go. **[Family19_L3]**<br><br>I: Right okay. So in that way, another question I have here is…in some respects how do you feel about the explanations you've been given?<br>R: To be honest with you, the fact that we did get the answers when we did ask, I'm pretty happy with. I mean I can let go of the smaller discrepancies of not being told what the medication is in critical care, et cetera. The fact that we got to speak to a doctor and a physio as well, we had a chat with. Not about medication, but other things. I'm pretty happy. If we asked for a doctor, they came, they come and speak to us. So I am pretty happy with that. It's something I can… I could cope with that.<br>I: And do you think you're getting enough information then as a consequence?

R: You never get the full picture with doctors unfortunately. They can be very vague. But you have to ask the right questions otherwise they don't answer properly, which I have found. And there has to be more than one of you because obviously two people have got different points of view, different questions, different sides of questions to ask. So it's easier when there's a couple of you asking questions. **[Family20_L2]**

I: And when you've been having these conversations with people, you said about the nurses there, and the Nursing Sister, have you initiated anything, or have they come to you?
R: I think it was a bit of both really. They've come to us at times, and then if I've seen the nurse sat at the desk and I've got a question, I would approach them.
I: Yeah, so, and have you been happy to do that, do you feel confident to do that?
R: Yeah. I'm happy to do that, I've had a lot of involvement with hospitals with our mum over the years, and my dad, so yeah, I'm quite happy to approach any member of staff and say, do you know what's happening, or, can you answer me this question? **[Family21_L3]**

R: And side effects-wise, I think, no-one really spoke about the side effects, until after she'd reacted to the oramorph negatively. They kind of said, oh it could be a side effect of the oramorph, you know, but other than that, I didn't really know much about, I mean, the antibiotics, I asked what they were for, it's for a possible chest infection, so that was explained, that was fine. And she was having paracetamol, she'd got a pain in her chest, this was before we knew she'd broken her sternum so they were giving her a paracetamol, and then she was taking paracetamol orally once she'd come round a little bit, because she didn't want any more of the oramorph.
I: And so, 'cause the oramorph was making her delirious was it, or…?
R: Yes, well I think the after effects of just coming round was, but that just made it a lot worse.
I: Yeah. And how do you feel about any of those explanations that you've been given?
R: Yeah, it was fine. I think it would have been nice to have spoken to somebody about the oramorph beforehand, but if it's happening in the night, obviously, it's not something they're going to phone up and chat to you about, they're just going to make that decision, which I totally understand. […] But yeah, everything was explained well, and I didn't come away thinking I should have asked more questions or anything like that. **[Family21_L3]**

R: Yeah, definitely, it is good to talk to people to say, well why are you giving her antibiotics? And they've said, oh, we think she's got a chest infection, we've sent off something for testing, but we're treating her for this in the meantime. So, you know, whenever a nurse came and did something, you know, gave her an injection in her tummy, I was like, well what's that for, and they would explain what it was for. And then I just think, well yeah, that's fine. There was no one time where I thought, actually, that makes me feel uneasy, I don't want you to do that. There was never that time.
I: Yeah, that's interesting isn't it? 'Cause you're getting the opportunity to ask questions. And when you ask those questions, do you find the response, well what's the response like?
R: Oh it's always been very good they've always took time to explain what they're doing, even like when they were taking blood, I was like, well why are you taking blood from her, what are you checking for, and they would explain what they were checking for. So yeah, the explanations have always been really good. **[Family21_L3]**

I: In what ways would it have been good to have been involved?
R: Well, it's just nice to know what you're taking, what it's for, and why you're not taking what you were taking before. You know, okay, yeah, we understand that you were on an antidepressant before you came in, but this is why we've stopped it, do you think that you might need something to replace it? How has your mood been, is it any better?
I: Yeah, absolutely, those sorts of questions then would have helped, would they?

		R: Yeah.<br>I: How do you think patients can be best involved?<br>R: Talk to us, just talk…<br>**[Patient22_L3]**<br><br>I: And were they, and were they, sort of…do you feel they were full did you feel they were, you know, you got all the details, as it were?<br>R: Yes. We were even told when he was first admitted to A&E that his injuries were, well, they were similar to what you get in a car accident. And at the end of the day he was 86 and it was explained to us, I mean, they were quite honest about the fact that he was seriously ill. And, [inaudible 00:09:24] try and save his life. So I've got absolutely no complaints at all, every question my brother asked or my sister asked, it was all explained by the critical care nurse why they were doing it. We asked about his oxygen levels, were they improving and they explained all that. We could see on screens, numbers, and they even explained, because we weren't sure what were his oxygen levels and what was something else because they can be a bit complicated, those. […] We'd worked out which was his blood pressure, but there was his heart rate and everything, and they even said, you know, this is this and this is this, and his oxygen level's this and this is what we're trying to get to. And they just explained things, as soon as we asked anything they explained it, there was no, sort of, fudging over it. Well we wanted to know because the thing is, if you get told the truth you can make measured decisions, can't you. You can…you know, my son wanted to come down and see him and he [inaudible 00:10:33] so I said, yes, come down. If they'd have fudged over everything and said, oh, no, he's going to be fine, you know, that wouldn't have been helpful because you can't make measured decisions of things when people want to come and see him, can you. **[Family23_L2]**<br><br>R: But if ever I wanted to ask a question, if ever I was concerned about something or I wasn't sure what was going on, the staff on critical care were amazing and would always take the time to sit down and go through it all with me again. And even though I couldn't communicate either because I had a trachea in that prevented me from speaking, even to the point where the staff they knew that I was getting frustrated because people couldn't lip read particularly well, so they got me a board so I could write on it and communicate that way. So, I feel like…yeah, it was a bit strange because obviously choices were out of my hand to begin with, but when I was communicative and once I had questions, people had all the time in the world to explain to me what was happening. So, I feel like I've had as much communication as I possibly could have. **[Patient24_L3]**<br><br>R: Yeah, it's just the nursing staff that administer the medications. Obviously they knew what I was on when I came from critical care and they just, is that working for you? Are you happy with that? Do you want this? Do you want that? So, they asked me every single time, it's not a, here's your medication you must have it. We progressed it a little bit in that speech and language therapists weren't completely happy with my swallow, so rather than trying to swallow tablets now, I get like a…what do they call them? Like an effervescent tablet, isn't it? […] So, everything's liquid form now. But, yeah, it feels like a choice, like it's a, you must take this. This is what it is…I also have an injection in my tummy once a day as well, I think that's a blood thinner because I'm not moving around as much. So, that's an option. There's also an option to have…oh what do you call them when you have the face masks with the…a ventilator, essentially like an inhaler. That's also my decision but they always try and give it me when I've got visitors, so I didn't have that for the first few days. But that was to clear stuff off my chest and every day I feel my voice is stronger. So I had this conversation with the nurses and I think they all tend to agree with me and we'll kind of like come to an agreement that it's not really necessary. So, yeah. **[Patient24_L3]**<br><br>Yeah, I feel like everybody's…we've all got one goal in mind and that's to get us better. They will listen if I've got concerns, if they don't have the answer they'll go and find somebody that does. Yeah, it's been a breath of fresh of air being on the ward. Yeah, everybody's very respectful, very mindful that you are an individual with feelings and thoughts. And if you've got questions and, like I say, if they don't have the answers they'll always find out for you. **[Patient24_L3]**

		I: Have you had any conversations with the doctor…the medical staff, the doctors or anybody else, any other health professionals?<br>R: Yeah, so I see a few different people. I see physio and they're trying to…but they don't have anything to do with my medication. I see a dietitian, so she's very much trying to get me to eat again so I can be more balanced out. And then I see the plastics team who did my skin graft. I have had one fraught conversation with plastics, just because of a procedure to remove…because I had like a vacuum dressing, that I didn't realise was…that the…I don't know what you would call it, it looked like polystyrene had been glued and stapled to me underneath the dressing. And so that needed to be removed and it wasn't done in theatre like it was done originally when I had my plastic surgery, it was done on the ward in my bay, with just a curtain around me, and the nurse staff were asked to do it. So, I didn't have any extra pain relief other than…oh, what do you call it? Entonox, which I thought would be perfect because I've had an ovarian cyst that ruptured in the past and in the ambulance on the way to hospital the Entonox was…it did amazing things, like it took the pain away, so I knew that I was able to use that and I didn't have any qualms about it happening. Only what I didn't remember was because my ribs had been pinned and it's happened quite recently, I'm no longer able to take a big breath full of air. So, actually the pain involved in the removal of the dressing was quite intense. So I have actually spoken to a ward manager about it and she was a bit upset on my behalf, and then within two minutes she had the plastics department speaking to me and telling me why they do it that way, and apologising because I hadn't realised I wasn't able to use Entonox until as well as I could have. So, I feel like it's been addressed, she said we definitely take that into consideration next time when we're removing a dressing when somebody can't use Entonox.<br>I: Yes, well that's really, really helpful, isn't it, yeah.<br>R: Yeah, it definitely is, honestly it was horrific but the fact that somebody's just taken five minutes to come out and just say, I'm really sorry that, that happened to you, and this is why they usually do it that way, this is probably why you felt more pain than we'd like you to have, and we'll change that from now on and we'll look at our procedures. She just ticked every box in my mind and I'm quite happy now that nobody has to go through the same thing as I did again.<br>**[Patient24_L3]**<br><br>I: I think…to be honest, (name), I think I've reached the end of what I need to talk to you about. Do you think there's anything you'd like to say or anything we haven't covered, anything we haven't talked about or, you know, perhaps go back over anything that we have talked about?<br>R: I don't think so. I think the main part of it all really, I just wish the communication and the quality of everything between the ITU and, you know, down to the ward, that level of care shouldn't change. I really want that highlighting, it shouldn't. I know they can't give one-to-one care and I totally respect that, working in the healthcare industry I know that first-hand. But that quality should never drop, it should always be top quality when they're with that patient. And half the time, you know, you feel as though they don't even know the person that they're treating. And that's the scariest part of it, especially as a relative, to watch that happen. **[Family25_L3]**
	**Timing of conversations - importance of, when on patient journey, how it fits with family, how it fitted with staff shift patterns and working, time of day.**	Well it was fine because whilst you're…with doing this, and so on. So, maybe I could have had a little bit more but at that particular point I obviously didn't feel that…well I didn't feel that I needed…I didn't feel I needed the information at that point for me to say to them, why? Maybe they could have initiated it and said, we've stopped it for x, y and z. Whereas I…but later on I might have said, well why is it I'm taking that and why I'm…or why am I not taking so and so? At the point that we're talking about I wasn't really interested whether or not…for what reason I wasn't taking them, put it that way […] mainly what they were concentrating on whilst I was in high dependency…because I was only in for one night and then they moved me into the ward that I'm now in, I think basically what they were more interested in, was saying are you in any sort of pain, and if so and the epidural is not…because it was constantly on, they said, if you do actually need to have more you need to just press this little button and it will give you more. **[Patient04_L2]**<br><br>I: Yeah, good. And do you think…I mean thinking generally then how do you think patients can be best helped to be involved in their medicines?

		R: I think that's a difficult one because some patients want to be and some don't. And maybe I thought that the…prior to it happening that I wouldn't have wanted to be involved but as it turned out I did want to be involved. So, I did ask a question. But if you'd have asked me beforehand, I'd have probably said, no, I'm not really bothered.<br>I: Just let them get on with it sort of thing?<br>R: Yeah, just let them get on with it. But as it turned out in the end it was like, well why are you giving me that, or, what are you giving me that for?<br>I: What do you think changed your mind now? What do you think?<br>R: I don't know, I can't say. Maybe I would have always said that, I just thought I wouldn't. And when it turned out in the end I did want to know. Whereas perhaps I'd thought, oh no, I'd rather not know, thanks, as I said, but as it…when it turned out in reality it was a case of, well actually, yes, I do want to know what you're doing. **[Patient04_L2]**<br><br>R: Up until last night I was all over the place, one night on intensive care, I take statins and they gave me three tablets when I used to take one. I asked them that, the nurse said that's what you've been prescribed, but it turned out that they'd overdosed me because when the nursing team did the handover in the morning, the nursing staff queried that, why did she give me that amount, and somebody had misread the amounts on the tablets.<br>I: So there's a bit of inconsistency there as well, isn't there, then?<br>R: Yes.<br>I: Both in what you were being told and what you were being prescribed.<br>R: Yes.<br>I: How do you feel about that inconsistency?<br>R: I didn't feel safe, to be honest. I mean, it was a junior nursing staff, two junior nurses on shift at that time, and I thought I don't want to spend another night on here. **[Patient10_L2]**<br><br>I think it's because what happens is the doctors do their rounds but, to be honest, I guess the thing that interested me as family was, how is my mum on the…and they were the ones that were caring for her on the 12 hour shifts so I was talking to them about her…how she'd been in the night time and how she'd been in the day time. I guess that was more…and I guess I trusted the drugs and med… Had Mum obviously not been responding to treatment or I thought that the sister's answers weren't fulfilling what I wanted to know, then I would have gone to a doctor but I didn't feel that I needed to, if that makes sense. **[Family11_L3]**<br><br>I: So you were involved in those conversations as well.<br>R: Yeah. I've been going up to see her at night because I work during the day, but anything that gets [inaudible 00:08:59]. In the case of my mum, she sometimes doesn't always take everything in, because it's a lot of information to take in. And obviously when I go up at night, there's always usually someone to speak to, if it's not too late, and they'll reiterate everything that they've said. And then I can try and feed that back to my mum in a way that she might understand it a bit more. **[Family15_L3]**<br><br>I: So basically, you are asking questions but they're also volunteering stuff to you if you're seeing them. But if they're volunteering stuff to your mum, you might then go back and check back with them. That's interesting.<br>R: Yeah. It's like the surgeons and doctors, I think they only seem to be there in the daytime. Obviously me and my sister have been going up to see her and we both work, so we have to go up late and there's something not always a doctor around. But there is usually somebody that we can ask, but I suppose they're looking after so many people, they can't really just come round and explain everything that's already been explained once, which I understand. **[Family15_L3]**

		And…so the transfer was…which is what you're asking about, was…well it was seamless, I'd say. I couldn't really find fault. If I'm really looking to find fault, the only thing I could say is that the lansoprazole was put in and I didn't know it was put it until I saw it on the ward. I presume it was started in the HDU, but I wasn't aware of it, because by the time I'd gone in in the morning, 'cause you're not allowed to go in the HDU until 11:00, whereas in the normal ward I was going in at 8:00 so I could see her morning medication, you see. And I could see it where of course I couldn't see it before. It was a perfectly sensible thing to put her on and…I was just slightly surprised because I hadn't been told. So I asked what I was and they told me straightaway. The nurse knew what it was. She didn't have to go away and consult a chart or anything. **[Family16_L2]**<br><br>R: Yeah. Well when we first get there my dad's chances weren't very good. So we were actually pulled into a room, well we were taken into a room, by the consultant and she explained what they were doing in detail. What they were going to try and do, what medications they were going to try and give him to try to stop him getting a chest infection, you know, the antibiotics and everything. She explained all that and she...and then, when he was in critical care, that was straight away, that was as soon as he was transferred to critical care, and then, from then on, we went every day. Me, my sister and my brother went every day and as questions came up, they answered us, they answered them. But they'd already been through the fact that, you know, what they were trying to achieve, you know, in critical [care 00:06:55]. But to be quite honest, they saved his life, to cut a long story short. Critical care saved his life without, you know, without any doubt. **[Family23_L2]**<br><br>R: Yeah. I think the only thing…some of the decisions have been quite prompt, so it needed to be a right now decision. And they're the ones that, you know, I think I'm more tense for (name), we need to make this choice right now. In some instances, you know, maybe that decision didn't need to be made as quick as what it seemed like it felt it had to be, if that makes sense. […] But again, I'm not a professional as such, if that makes sense, so I, sort of, follow their lead with it. But, I mean, sometimes the impression that you get is that it's an imminent decision that needs to be made and you're waiting three, four hours for anything to actually happen. And you're, like, does it really need to be that urgent? **[Family25_L3]**<br><br>I: In what way do you feel their job is not talking to you, their job is doing something else, injecting something, or doing a dressing, or going to see a patient?<br>R: It's just, how I put it is like, sometimes you feel that, like, the nature of how things are structured at the moment, sometimes you get worried that you're taking somebody away from what they're doing.<br>I: Yeah, yeah. So, you're using up time that could be spent elsewhere?<br>R: Yeah, that's, kind of, more like what I'm going for. Because, as I say, I've never felt like I couldn't, but it's more like…<br>I: No, but you do feel…<br>R: …how they're functioning at the moment, you just feel like, oh god, like, if they're doing something, you do feel like you're taking them away from that, that could be done now, rather than later. And you don't know what's coming around the corner, as well, and you feel that, yeah, if you take people away from certain things, you're feeling just a little bit guilty. Especially when somebody is into, like, even changing all the medications, and going back to, like, obviously what this is about. […] You feel like you're taking them, or distracting them a little bit, which makes me feel a bit guilty. **[Family29_L3]**
	**Verbal or written information - attitudes towards, benefits or limitations**	R: Probably as much me not being able to focus. I think the problem is, you're told stuff, and if I go back in, like, my work situation, when I was working, if my gaffer came round and gave me an instruction, I'd get it written and I'd know what to do, and then I would look at it, and go, and this is wrong, and you'd go back to him and [inaudible 00:07:57]. But because it's all verbal. I know them pamphlets they get, can be a pain, but maybe if they had an iPad where they could pull up just certain areas what they want to emphasise to you. But the guy was very, very clear. And he spent time with me, he didn't just come in, bang, bang, bang, bang, bang, bang, make just ten bullet points and go out. He came and he took his time with me. **[Patient01_L2]**

		I: And in terms of, you know, receiving information from them, do you get any…as well as being told explanations, talked to about it, is there any written information that's given to you or to your dad or anything? […] Do you think that might be something you'd want or would that be something that would be useful?<br>R: It would be useful if they put it into his care when we take him home, because knowing how busy obviously the hospital is and everything, it's like an added job for them and the nurses and whatnot. Yeah, so as long as someone gets a grip on what's going on and everything, they put it into the end of the care of when he gets discharged, everything that has been done and what needs to be done and everything and the medication's written down, that would be a nice thing.<br>**[Family03_L2]**<br><br>R: Yeah, it probably would be more…something more individualised, a summary of what you're on and why you're on it and then you can actually look at it and think, oh perhaps I should ask so and so then. That would help to prompt you to ask a question rather than you thinking afterwards, oh perhaps I should have asked so and so. Or when somebody says to you, oh did you ask them so and so, then you think, oh I never thought to ask them that. […] Whereas if you've got it…if you've got a summary of it in front of you you can then use it as an aid and say, oh well what exactly is this, or, why exactly is this, and what are the effects of it, and what happens if there…what happens if I don't do it, what will the effects be if we didn't do it? **[Patient04_L2]**<br><br>I: Written information given to you, you know, an information sheet or anything like that?<br>R: There was pre-op information about what medication I needed to stop but afterwards, no, not really.<br>I: No, so there's no summary of your medicines or anything like that. Do you think that it's better to have a conversation about your medicines or is it better to have written information or a combination of the two or what?<br>R: Yes, I think a combination of both would be beneficial to the patient.<br>I: Yes, in what ways would that help?<br>R: Well, for the operation, you know, if you're given the information telling you about your medications, what not to take, when to stop taking the medication, and then possibly when you're in hospital, something to explain to you why we're stopping the medication, or we're slowing that down or… **[Patient10_L2]**<br><br>I: But is there a particular way you'd like to receive information from them, in terms of is it better that it's a face to face conversation, or is it better that it's… Would it be better with an information sheet, or something written down?<br>R: Yeah, I would say that maybe sometimes when we have had the conversations, especially with the surgery, they do sometimes explain things a little bit…it's a lot to take in in one go. You try and remember things after, did they say this, did they say that? I can't remember. Is that what he said? Like yesterday, because we've had that many conversations with different people, maybe some kind of way of noting it down without sitting there taking notes yourself. Maybe that might be helpful, yeah, because there's a lot of information to take in sometimes. And obviously when you don't know, where you've got no medical background, sometimes it is quite a lot to take in. **[Family15_L3]**<br><br>R: Well every box was labelled up of course with the instructions. She went through the complete list with me…just one sec…very, very carefully, I'm just seeing what information she [submitted 28:47]. I don't think she sent me a list out unless (name) has got it somewhere. There might have been a list…I: Yeah, like a…R: Oh yes, here it is. Yes. They sent me a complete list out of everything that she was supplied with, including sharps, containers, anti-embolic stockings, all the medications she went in with, of course some of them…we took the medications in as requested so some of them they brought out were ours, prior to the hospital visit and some were theirs. Was there anything extra? No, they gave her some Laxido, which they explained was for constipation should she get it, which would be quite a novelty because she went in taking 24 loperamide a day plus six codeine a day and she still got diarrhoea three/four times a day.

		R: So far, it seems to have worked. So that was a new thing that was sent out and I did know what it was but she did explain it to me. And she got some Oramorph which she has taken in the past, but not recently. Yeah, they gave me a complete list of all the medication she was on. They went through it I thought quite thoroughly. I know [they have to know everything 30:31] before she started but she did go through it properly and carefully and, yes, on discharge. Yes, I thought it was very good. **[Family16_L2]**<br><br>R: She just phoned up and explained what was happening, this woman.<br>I: Right.<br>R: And then she said, your doctor will be in touch, and they'll decide whether you need the antibiotics.<br>I: Right, okay.<br>R: And it all happened within 24 hours.<br>I: Right, right. Right, but you got, as you say, you got that email, yeah.<br>R: She did send us some information via email, to download and read.<br>**[Family17_L2]**<br><br>I: So no general information thing or anything like that.<br>R: No.<br>I: Do you think that might have been helpful at all at any stage?<br>R: I don't think it really mattered in intensive care just because the information was easier to get hold of. I think just even like a little…not a diary but something on the bed, like on the clipboard what the doctors use, just something. I don't know. Just so you can have a little read like oh, this morning his stats were fine or need to speak to the doctors because of this. You're a bit more informed then. I think if someone has been in intensive care for that long and they've been sedated and whatnot, and they're not 100 per cent with it and you're a little bit confused, when you go to visit them and you say oh, what did the doctor say and they're like oh, don't know, I can't remember. […] And then it's not as easy as just finding them to ask them what they said. You can't always know. Then you would ask the nurse and they're a bit like oh.<br>**[Family18_L3]**<br><br>I: Have you been given any general information in terms of written information about any of these medicines? Is there anything sort of like a general information sheet, or a summary sheet, or anything like that?<br>R: I've had a… They did a diary for me which is being documented every day, you know, what nurses were looking after me, and what happened to me that day, you know, and did I have any ups and downs, that sort of thing […] kind of thing, gives you quite a lot of information and insights, what was going off on that particular day […] And I've also had some leaflets, although I can't remember which ones they are or where they are. But I have had some other leaflets. **[Family19_L3]**<br><br>I: So written information would've been good or a summary of what was going on or something like that would've been good then?<br>R: Yeah, absolutely, yeah. Either for me. I mean if they want to talk to us about it, fine. If they want to give it in written form. Fine. Talking and written form would be perfect because obviously you've got other things going on in your life, like children, work…<br>I: Yeah, precisely.<br>R: …household things. When you get a chance to relax you can sit down and have a read and then refresh yourself on what's been happening. **[Family20_L2]**<br><br>I: The other question we have here is how you like staff to communicate to you. And one of the things we particularly ask people is, whether you would have liked any written information?

		R: I think written information would have been good, because you're going through a stressful time, and you can't always take on board what you're being told at that moment. And so it would be good to have something to refer back to.<br>[…] So yeah, written information would be good, I would say that, yeah, definitely.<br>I: Like a sort of general information sheet about the medicines or…?<br>R: Yeah, just general information of, a) what they've assessed to be wrong in the first place, and, b) what they're doing to put that right, make them better, and what meds they're on and why they're on those meds. **[Family21_L3]**<br><br>I: Right, okay. Yeah, that's good. And is it helpful to have written information?<br>R: I think so, yeah, I think so because I don't think my dad realised how seriously ill he was, and it has been explained to him now, that he was very ill. And all this information has been given to him in leaflet form, and, like I say, I shall read that, when he's back home I shall read it. But I've left him to read it because obviously the injury's happened to him not me. So, you know, it gives him something to look at and read. **[Family23_L2]**<br><br>I: Would, sort of, some written form of information be useful?<br>R: In my unique scenario, with myself and my dad, sadly, we are both dyslexic, but this is quite a specific scenario, but that's something you could bring up.<br>I: No, that's true, it's very important, yes<br>R: Yeah. But I feel that, on a general basis, it would help being able to have at least some form of written communication. Because I know that, obviously on the ward, there is a lot of consistently, like, taking people away from their job, and like, that adds to it, you feel like you're taking people away, even to ask these questions. So, I think some scenarios, not specifically to mine, but I feel that written communication would quite help, because it means that you're not taking people off, feeling guilty, or feeling like…not to say that anybody made us feel like that at all […] It's that guilt of you're feeling like you're taking somebody away from their job.<br>**[Family29_L3]**<br><br>I: Would that have helped, do you think, to have had written information?<br>R: I think that would be helpful, yes.<br>I: In what ways do you think it would be helpful?<br>R: Well, you can't remember everything, can you, it's impossible to remember everything they tell you. It would be helpful to just think, yeah, I can remember them saying that but to have something in your hand, you don't have to keep asking people sometimes. If you have something in the hand, you're not liable to forget it…. **[Family30_L3]**
	**Which health professionals engage with patient. Patient preferences (if any)**	I: Oh, yes, we were talking about the different people who have conversations. I think one of the things is of interest to us is what you would prefer, what would you…what do you like people to do when they're talking to you about your medicine and who would you like to hear from?<br>R: I don't mind, either the nurses or the doctors to be honest. I don't know, either/or. **[Patient02_L2]**<br><br>Well, very kind, very empathetic. The doctors, the consultants when they do their rounds are very presumptory you know, they don't say a lot. You sometimes have to, you know, you've thought of a question and they've gone before you get to ask it. And then that's ward rounds, isn't it? But I've had two sessions today, one with the physios, who were brilliant, brutal but brilliant. And one with the, the stoma nurse who was very empathetic, she was lovely, she reassured me actually. **[Patient08_L2]**<br><br>I: Those conversations, do you anticipate then during this visit to have a conversation with somebody before you leave?

		R: Yes, when I've been in before, normal procedure has been on the day of discharge or a couple of days before to be told if they're changing the medication or if they're sending me home with any extra medication and then the reasons why, they usually discuss.<br>I: Yes, and is that normally a pharmacist or a nurse or a doctor who does that?<br>R: Usually the pharmacist but sometimes a nurse will come round and explain to you.<br>I: Right, yes, depending on…<br>R: If the pharmacist is busy or not. **[Patient10_L2]**<br><br>R: I would say a lot of that was explained by a sister that was doing…when Mum was really poorly and she was, kind of like, working with her constantly there was a sister that worked with her and she explained that to us in quite some detail.<br>I: So it was basically around nursing staff then who would do that.<br>R: Yes, I would say most of my conversations were with the nursing staff because they were the ones that were doing the main care for her. I mean, had I requested a conversation with a doctor that would have been…you know, that was possible but I found that I was happy with the information that I was getting from the nursing staff, particularly this…this particular sister was really good. **[Family11_L3]**<br><br>R: I think it's because what happens is the doctors do their rounds but, to be honest, I guess the thing that interested me as family was, how is my mum on the…and they were the ones that were caring for her on the 12 hour shifts so I was talking to them about her…how she'd been in the night time and how she'd been in the day time. I guess that was more…and I guess I trusted the drugs and med… Had Mum obviously not been responding to treatment or I thought that the sister's answers weren't fulfilling what I wanted to know, then I would have gone to a doctor but I didn't feel that I needed to, if that makes sense. **[Family11_L3]**<br><br>I: Right, okay. And was this, in terms of thinking of timings then, was that around the, sort of, when you're recovering, when you're back on the ward, you know, you're getting these painkillers, were you getting any other medicines as well there?<br>R: I had a daily injection which I was told was for thinning blood.<br>I: Right, okay. And you were told that was for, as a blood thinner.<br>R: That was what it was for.<br>I: Yeah, right. Okay. And that was on a daily injection?<br>R: I had it every morning, yes.<br>I: Right. And who was involved in that, the nursing staff, presumably?<br>R: The nursing staff, yes.<br>I: Yeah. So, they came round.<br>R: Yes. Well, I mean, there was only eight of us in the ward, so by and large there was somebody there all the time.<br>I: Right, okay. So, there was always a nurse in the room with you…yeah.<br>R: Well, there are, kind of, two parallel wards and the nursing station at the end, if you see what I mean.<br>**[Patient14_L3]**<br><br>I: Who were the people who were having these conversations with you then? Was it the nursing staff or the doctors or…?<br>R: The nursing staff in Critical Care, they informed. Everything we asked, they had an answer, or they'd try and find out the answer. And since she's been back on the ward, just everybody involved, so all the nurses and… We spoke to…well, I've not, they've been there, but my mum's spoke to the physiotherapist and the dietitian, just to talk her through about getting a bit of exercise and saying it's important to sit up every so often rather than just lying in bed. Trying to get her to walk around a bit if she can. **[Family15_L3]**

		I: Precisely. Yeah, that's interesting. 'Cause actually I spoke…that was at the point of discharge, was that one of the…who was that doing that? Was that the nurse or a…was that a…?<br>R: It was one of the staff nurses. I mean, I don't think she was the sister in charge of the ward. I found it a bit confusing actually because there were so many changes of staff. But she was a senior nurse, let's put it that way. And she was training a student nurse at the time. **[Family16_L2]**<br><br>I: Yeah. And are you going, are you visiting regularly in terms of, and so, do you get to see the staff regularly, and have regular contact with them?<br>R: I go every day […] And whenever I see, like a ward sister, I seem to ask them more than the nurses, because they seem to know more […] And the nurses are very busy. So, if I get worried about anything, I will look for a nurse and ask them […] But they're in and out to him all the time, anyway, so you can always ask a question.<br>I: So, you ask the ward sister. Do you talk to anybody else, like the doctors, or any of the other…?<br>R: I've not seen, I can honestly say, I haven't seen a doctor while I've been there. **[Family17_L2]**<br><br>I: And what were those conversations like? Were they useful or helpful or…?<br>R: I think they were helpful. I think obviously in my own experience working in that environment, I think nurses in general are a bit better at explaining things in layman's terms rather than doctors and consultants who use big words and stuff. But yeah, obviously at that time when things are quite up in the air, it's a lot to take in anyway, isn't it, for anyone. But I think the nurses did really do a good job in explaining what was going on in regards to medicine. **[Family18_L3]**<br><br>R: Well, I think in intensive care, I was more than happy just to speak to the nurse, because the consultants and the doctors used to meet at lunchtime and they have a bit of a mini MDT meeting, so all the different specialities will discuss the patient and then they will feed back to the nurse like the plan going forward either for the next day or the next couple of days. So what used to happen is, once I got there at tea time I'd know a bit of information, so she would be able to feed back a bit of something to say oh, the doctors aren't happy with this, or the doctors want to review this or he's going to have this scan. You'd get a lot of information. Whereas I feel like at the minute I don't know who's looking after (name) I don't actually know who the named doctor is or who should be reviewing it. And I think because the nurses are that thinly spread on the ward, I don't actually really know the nurse. For instance, a minute ago I just grabbed a lady that walked past and I said oh, I'm just looking to find (name's) blood results for today and she was like oh, I don't have that information, I don't know which nurse it would be. **[Family18_L3]**<br><br>I: Yeah. And when you did have any interaction with people in ICU, who was it mostly that was talking to you?<br>R: So it was mainly the nurse that was looking after her that particular day. I did speak to one of the consultants one day, that was when she'd only really just come round, sort of a couple of days after. But the main conversations were with the nurse that was looking after her that particular day. **[Family21_L3]**<br><br>I: …things like that in that, isn't it? In terms of…I suppose because you say you haven't had these conversations, it's difficult to think of it, but how would you like staff to communicate to you, what would you like them to do?<br>R: Is this regarding critical care or now?<br>I: Well, either, either, let's just think about it as both, the two moments as it were.<br>R: Because on critical care I don't think they could have spoken to me about anything because I was absolutely out of my tree. […] But when I've come onto coronary care and then I'm [inaudible 14:31] now, they could have just talked to me, have a conversation.<br>I: Yeah, yeah. And who would you most like that to be?

		R: It could be a nurse, it could be somebody from pharmacy.**[Patient22_L3]**<br><br>No, no, they've not given us any indication of the plan going forward. We literally get it day by day and that's if we're lucky enough to get the sister on the ward because the sister will explain it. If the sister is there, she will sit down and explain it. If the sister's not there, they'll literally explain to you what they're doing in that moment and they can't give you anything more than that. They just say, the consultants know what they're doing **[Family25_L3]**<br><br>R: It's not strong at all. And we're not allowed most of the medications, and that's what really annoys me. And, yet, we're left to be in pain, or have an alternative that isn't as good.<br>I: Yeah, indeed.<br>R: And that's very frustrating.<br>I: Right. And who has talked to you about this issue around, you know, what medicines you can have, and so on, who's discussed this with you?<br>R: The renal team. When you ask them if they can increase your dosages for pain relief, for example, you know, to a safe level, or you know, get you something stronger, they won't do it.<br>**[Patient27_L3]**<br><br>R: A lot of the discussions were with the nurses that were looking after her at the time […] Obviously, with the critical care unit, it was like one nurse per person, so like, it was a very open conversation, with the nurse obviously being there, it was a lot easier to just ask them. But if there was, like, times that their potential consultants, or I know that sometimes if there was a major change, or any particular issues that we faced, a doctor would come to talk to me.[…] Like, it obviously just depended on the situation. Because with my mum, she was stable for quite a long period for her stay, it was more, she needed the care that was supplied by the unit. But when there was, like, any major changes, it would be a consultant or a doctor that would have informed me […] But as day by day went, it would generally be the nurses that were looking after her in the daytime, that would give me the update. **[Family29_L3]**<br><br>R: You see, I didn't particularly mind it was the nurses relaying with me, because obviously, I think, just from my assumption, when it comes down to the consultants and doctors, it is nice to have the update when something particularly major happens […] But due to the nature of the nurses being there constantly, and observing my mum the whole day, sometimes it did feel a lot better just asking the nurses. Because, obviously, it's nothing like, nothing bad or anything, it's just the fact that, with consultants and doctors, kind of, being summoned, we'll call it, when something either has gone wrong, or it is something major, you do feel a lot better being able to talk to a nurse, where they've been able to observe my mum, and look after her all day. They've been there from, literally, the morning until the evening, they've been there most of the time […] And sometimes, obviously, when you are relaying with consultants, and doctors, it is very much like, it's an as and when, if something has gone wrong, that's why they're there, or tend to be there, or when it's something that they have to observe. Because with my specific circumstances with my mum, she had a lot of input with stoma team, and she had a lot of input with, I believe it was the tissue specialists, as well. Because the nature of the abdominal wound that she ended up getting, I believe they are using, it's a vacuum sealed dressing that is constantly draining it.**[Family29_L3]**

**Theme 5**	**Initial and Final Theme - Engagement in practice**	
	**Sub-Theme**	**Quotations**

	**Acquired knowledge and understanding from interactions**	I: Right, so have they sort of…just talk me through that, has it been a sort of staged process of slowly coming off the painkillers or have they added some new ones in also?<br>R: Yes, I think I started on [inaudible 0.00.47] and then they are slowly thinning them down sort of thing.<br>I: Right, so you…<br>R: I was on [Oramorph 0.00.57] as well, but I don't really use that anymore. […] They just explained that obviously the stronger pain relief in my back would be stopping so I'd probably need some other and explained that if I needed the Oramorph was if I asked for it, I wouldn't just get given it, it was if I was in pain I'd ask for it at any point and they explained how over many hours I could have it between that as well, so it was explained well to me to be fair. **[Patient02_L2]**<br><br>R: Yeah, his regular medication that he's been on, one has stopped whilst he's on antibiotics, it was also stopped in a critical unit because he was on a lot of antibiotics, and they stopped one of his Allopurinol tablets last week in ICU, and they've now reintroduced it, but they've lowered the dose.<br>I: Right, okay. And who…was it, again, the doctors that talked to you about that?<br>R: Yeah, it was the doctors saying it all first.<br>I: Do you know why those medicines were stopped or why they were changed?<br>R: Yeah, when he was in the critical unit his kidneys weren't functioning properly, so they stopped his Allopurinol medicine because that affects the kidneys, so they reintroduced it just this week whilst he's on the ward, but a lower dose.<br>**[Family03_L2]**<br><br>I: Right. And you were told…were you told the reason why they were added?<br>R: Oh yes, you know, what they did with the heart one kind of slowed it down, one rushed it up. It's quite funny how they applied it. There was…it was usually ….explained but they did say, you know, you do have to take these for the rest of your life, et cetera. So, they explained the importance of it. So, you know, once again, trust. **[Patient05_L3]**<br><br>… they were explaining to me why she'd got a lot of water retention and they explained that the medication that they were giving her, I think, to help her heart was affecting the kidney function which was the water retention. Then they talked again about medication that they were giving to try and activate the kidneys again […] Yes, in ICU so when she came out of her operation and then she had to go back in almost immediately because there was internal bleeding and then they sorted that out. Then they were struggling so much with her heart then they had to give her some medication which then affected the kidney function, then because she had so much water retention, they were thinking she'd have to go on dialysis they then had to give her something to, kind of, counteract the kidneys to try and… They were just mentioning that they were trying to balance the medication between supporting her heart and not affecting the kidney too much. That was what I was aware of. **[Family11_L3]**<br><br>Well, I mean, I've got a complete schedule for everything I have to take and where I have to take it. And some of the tablets say reintroduce full Atenolol on 6 March or whatever it is and, you know, start half the dose of Lisinopril on 7 March, or whatever it is, you know, it's all down. There is a schedule, but I do need…I've got to have my staples taken out today, so I try to get round the 08:00 phone call thing and say, well can I please come and talk to someone a-sap about what's going on, because although the Furosemide is working, I've still got plenty of fluid on board and you can probably tell from the way I'm breathing, it's not particularly clever, but what I don't want to happen is the same things happened last time, I do not want to be taken in an ambulance ever again in a hurry. So, I need to manage that a bit better I think, and just make sure it is a session with the GP as opposed to the paramedics. **[Patient12_L2]**

…it was one of the ward doctors, I think, who came with the blood pressure medication, would certainly be in that case, who came with the notion of this reintroduction. I can't remember whether that was before or after the oedema showed his head, but […] But it was kind of presented to me as, this is how we do it, this is how we reintroduce and I know what it was. But the oedema was apparent and it was only when the oedema became apparent and that's probably…I don't know whether it happened in particular this way, but I think it did, I was walking thinking blimey, my legs are getting bigger and bigger and I was walking around the ward trying to get mobilised […] I wonder whether there's a set of weighing scales anywhere around here. Well, there was and my weight before the operation was 82 kilograms, I got on the scales and I'm 91 kilograms. So, I thought well, a) they've cut a lot of me away, b) I haven't been eating and c) this must mean that the rest of the difference is all water, you know. Well, I did mention this to the nurse and I think it was at that stage that the doctor came in and said oh, we will be reintroducing the Spironolactone which is the diuretic, straight away, have some of this now. […] And then the schedule for reintroducing the rest of the Atenolol and the whole of the Lisinopril was on the discharge letter. **[Patient12_L2]**

I: Right, okay, yeah. And in terms of then…because you talked earlier about some changes to medicines. So you said there'd been a change to… Oh, there wasn't a change, the antiseizure medication, she continued on that.
R: The antiseizure medicine just went through the canula because she obviously couldn't take tablets.
I: So that was the change.
R: Yeah, it wasn't the actual change of the medication, it was just the form of how she took it.
I: How it was done.
R: Yeah. The antibiotics have changed. I don't know what she was taking before, and they've literally just changed today. […] I don't know why they've changed them or anything, I've no idea. She just told me that they've changed her antibiotics, that's all I know. **[Family15_L3]**

R: I think they probably had told her, we're giving you this to help your stomach, perhaps that's what they said, but I doubt she would really be able to remember it to be fair. I mean, she does take quite a lot of medication, so one more capsule [in there 07:03] is not going to be necessarily noticed even. She has…she's taking a lot less medication than the medication that she went in with, but she was having to have 24 loperamide a day, which…and she doesn't take any now. The thyroxine is 75 micrograms, they've kept her on that. Amlodipine one milligram, they kept her on that. Multivitamin and minerals, not sure about that, whether they…she said she hadn't had a blue tablet, so maybe they didn't give her that but it's…as I say, it's only multivitamins, not essential. Her calcium, they've kept her on that. Creon, she was given that. Codeine phosphate, they did take that away because of all the other medication she was on, but then that was reintroduced as a painkiller. She was actually taking it, not as a painkiller, to reduce the diarrhoea which is the whole purpose of this operation. **[Family16_L2]**

From the critical care to the respiratory ward, that's when we found out that the clot was actually…there wasn't an artery that wasn't being blocked by this clot. So they then told that it was thrombolysis that they gave me father. Now it's something that I think is quite a common…it's quite commonly given, but in small injections subcutaneously into the stomach. Not in the method they gave to my father, which was through the vein and quite a large dose, a fairly quick time, to break up this clot. Other medicines, there was something for his indigestion, it was pro something. There is a blood thinner that they were giving him, I don't know the name of that. He had a powdered 30 day inhaler or 30 uses. Something like that. Upon coming onto the respiratory ward, he never had any regular painkillers. If he was suffering he told them, then they'd bring him some sort of painkillers which was Co-codamol, I believe originally. And then when the doctor came in, I believe it was the beginning of this week, he then…or towards mid-week rather, that they decided they were going to give him some regular painkillers. So Paracetamol, Codeine and Oramorph if needed, which the Oramorph they never gave him, because he's been quite happy on Paracetamol and Co-codamol. **[Family20_L2]**

		R: Yes. And that was something that I did bring up when she was on Critical Care, because she was on antidepressants beforehand, and I sort of said, could some of her reactions be because she's literally gone cold turkey, because we found out that they'd stopped anything that she was on beforehand. And they said it could, but it was more likely that it was to do with waking up from the sedation, and the current situation, than sort of tablets that had been stopped.<br>I: Yeah, of course, yeah, precisely. Yeah. So do you know whether those antidepressants have been restarted now?<br>R: They haven't been restarted, and I do know she had a conversation with one of the doctors about it, just from speaking to her. She's quite happy to try not to go back on them, 'cause she also used to vape as well, and she's kind of said, I'm not going to go back to vaping either, I've lasted this long, so, she was going to try not taking the antidepressants and the doctor just, nurse just said to her, if you do get low mood speak to us. And I think she's got to have something different because of her heart now, and they've already made recommendations to her GP should she wish to go back on them. At the minute she's staying off them through choice. **[Family21_L3]**<br><br>I know, yeah. And back to the ward on Saturday, the third, so he was in critical care for a week, gradually getting better. And then on his transfer to the ward, he was totally with it, you know, he was back with us. So I asked him yesterday, because I knew I was going to do this interview today, about his medication and everything, and he knew everything, all the medication that he was getting, from the nurses. So he was able...it wasn't a conversation I needed to have with the nurses, if you know what I mean […] Because he knew what he was getting, he was being told what he was being given and he knows what he should have been taking and shouldn't have been taking. And he said that everything that he was taking was right, it was what he was on before. **[Family23_L2]**<br><br>R: Yeah. Well when we first get there my dad's chances weren't very good. So we were actually pulled into a room, well we were taken into a room, by the consultant and she explained what they were doing in detail. What they were going to try and do, what medications they were going to try and give him to try to stop him getting a chest infection, you know, the antibiotics and everything. She explained all that and she...and then, when he was in critical care, that was straight away, that was as soon as he was transferred to critical care, and then, from then on, we went every day. Me, my sister and my brother went every day and as questions came up, they answered us, they answered them. **[Family23_L2]**<br><br>I know a lot of her medication isn't taken intravenously anymore, most of it, or if not all of it, is taken orally. Like, paracetamol, I believe she has, now, oramorph, when she's having a particularly painful reaction to her stoma at the moment. But, yeah, a lot of it is just now, just taken orally. I know that a lot of the medication she was on as well, I believe she was on amoxicillin, when she was in critical care, which obviously, was to ensure that she wasn't going to have any infections. I know now that she's not on that on the general ward. But, yeah, I think, they're really the changes of, like, that I could note, anyway. But, yeah, I think the changes that, well I think the changes that were made, it's more because of the fact, with her actually being aware, and with her being more able to do it herself. I think, from what I can note is, it's more like the emphasis is on being able to provide them for her, orally, and like, as and when she can take them. Rather than, obviously, just being able to administer them to her, because she wasn't able **[Family29_L3]**
		I: Because actually that's my next question, actually, was around these changes and, you know, who made the changes to your medicines? Do you know who made those changes?<br>R: I don't know who made the changes or why the changes were made, other than the blood thinners…<br>I: Apart from…yes.<br>R: I'm aware of that, the reasons behind that. **[Patient10_L2]**<br><br>I: I presume that that was to do with the operation as to why they were changed. That's interesting. I suppose the next part of that is would you have liked to have known why those…?<br>R: Yes, I would have done, yes.

		I: In what ways would it have helped you to know?<br>R: It would make things clearer as to why they were stopped, and the reasons behind stopping it. **[Patient10_L2]**<br><br>That's what she, kind of, said. Interestingly though you're talking about medicine, she said, my mum did say to me today, she said, I don't know what medicine they've given me this morning, (name), she said, and she talked about the tablets so she's not been told at the moment what medicines she's having and what it's doing. I mean, I'm sure if they asked but they're not actively telling her and I guess, I could have at that point said to her, Mum, would you like me to ask? **[Family11_L3]**<br><br>I: Right. So, it's for you, it's the right one. What do you feel about the fact that no-ones talked to you about that?<br>R: I would have preferred if they had.<br>I: Right. And do you think you might try and find out from someone or…?<br>R: I'll try and find out what it's for before I use it.<br>I: Yeah, okay. There should be the information leaflet in there, shouldn't there, which might help, I suppose.<br>R: Yeah.<br>I: So, you would've preferred…?<br>R: I'd forgotten all about it.<br>I: Yeah. You would have preferred to have known why…?<br>R: Yeah. Well, it's slightly disturbing just to find this thing at the bottom of the bag and wonder what it's for, but I did check it and it was for me. **[Patient14_L3]**<br><br>Now she did have a nasal feeding tube so…I don't know much about those but I assume they can cause irritation to the stomach and so they'd introduced that in to her diet…not to her diet, to her medication to reduce the acidity and any potential irritation from the nasal tube. That's my best guess. I was not told why it was **[Family16_L2]**<br><br>I: Do you know if those have been, since he's been on the ward, do you know if those have been restarted, at all?<br>R: I don't know.<br>I: They haven't had that conversation?<br>R: No.<br>**[Family17_L2]**<br><br>R: Nothing. No. And I [inaudible 0:08:46] I was already on those tablets […] The only one that's an additional tablet, is the extra blood pressure tablet. Everything else I was already taking.<br>I: You were already taking beforehand. Right I see.<br>R: The statins. Yeah.<br>I: Right. Have they at all changed in terms of the dose or the number of times you're taking them a day? Has there been any changes along with those medicines then?<br>R: I couldn't tell you. I don't think so, to be honest with you. **[Family19_L3]**

		Well, I mean, I've got a complete schedule for everything I have to take and where I have to take it. And some of the tablets say reintroduce full Atenolol on 6 March or whatever it is and, you know, start half the dose of Lisinopril on 7 March, or whatever it is, you know, it's all down. There is a schedule, but I do need…I've got to have my staples taken out today, so I try to get round the 08:00 phone call thing and say, well can I please come and talk to someone a-sap about what's going on, because although the Furosemide is working, I've still got plenty of fluid on board and you can probably tell from the way I'm breathing, it's not particularly clever, but what I don't want to happen is the same things happened last time, I do not want to be taken in an ambulance ever again in a hurry. So, I need to manage that a bit better I think, and just make sure it is a session with the GP as opposed to the paramedics. **[Patient12_L2]**<br><br>She has…she's taking a lot less medication than the medication that she went in with, but she was having to have 24 loperamide a day, which…and she doesn't take any now. The thyroxine is 75 micrograms, they've kept her on that. Amlodipine one milligram, they kept her on that. Multivitamin and minerals, not sure about that, whether they…she said she hadn't had a blue tablet, so maybe they didn't give her that but it's…as I say, it's only multivitamins, not essential. Her calcium, they've kept her on that. Creon, she was given that. Codeine phosphate, they did take that away because of all the other medication she was on, but then that was reintroduced as a painkiller. She was actually taking it, not as a painkiller, to reduce the diarrhoea which is the whole purpose of this operation. So that was taken away at one point because it was being replaced by other things. And then it was reintroduced on the ward to help with the pain relief. The ondansetron, they took away. And I asked her about it and she said she had no nausea so there was no point in taking it. The [letrozole 08:27] tablet, she thought one day they might have missed that in the HDU unit, but she always had it in the…on the ward. So in terms of transfer, everything was perfect. She…having had her operation, they've stopped any paracetamol. That's it really. Yeah. That's everything. **[Family16_L2]**<br><br>Well, they'd already reduced a lot of his medications in intensive care. So he was on obviously sedation medication. That got stopped when he was un-sedated. He was on clonidine, quite a high dose. He'd been on a weaning dose on the ward. But that is obviously initiated by intensive care. Yeah, a lot of his medications had been stopped. So he was on noradrenalin for his blood pressure. He was weaned off that. Yeah, he was weaned off most of his medications. I think the only medications that were being transferred from ICU to the ward are the antibiotics, because he's on a long course of them, because he's still got high infection markers, and a clonidine weaning dose. Apart from that he's not been on any medicine. **[Family18_L3]**<br><br>No, my dad told me. They gave him the anti-blood clotting, the blood clotting injection up on critical care before he came down to the ward. And the first question they came over and asked my dad was, had he had that injection? And asked me as well, has he had this injection up in critical care? And I said, yes, me and my sister were both there when he had it and my dad said, yes, he'd definitely just had this injection in his stomach. So that was the first thing that happened. And then, as for his medication, like I say, he was on top of that anyway because he was okay, he was with it, he knew what was happening. He's told me that a couple of his drugs were altered for a reason but I've not asked him what the reason was, what that reason was. He said it had something to do with his blood pressure, I think he mentioned one of his medications, they altered that from, I don't know if it was when he was up in critical care, but he seemed to know all about that. He started [inaudible 00:15:18] they started me back up, back on that again. So he was aware of all that, he was aware. Like I say, if it had been my mum, I would be more helpful to you because I would have been, I'd have been the one. **[Family23_L2]**
		I: Yeah, it's interesting. And have any of your medicines been changed? Have there been any specific changes to your medicines since you've left ICU that you know of?<br>R: Only two, and I can't remember what they are anyway. But there were two which I'm not quite sure whether it's changed or whether it's just added to. I think…<br>I: They've changed the dose, or something, yeah.

		R: No, it's just there was…there were very few doses changed in that as I remember. I mean thinking back about it I don't think there were any, but there just the two new…[medicines added]. **[Patient05_L3]**<br><br>I: Right, okay. So in terms of then, you know, what's happening now on the ward, has anyone come to talk to you about medicines, you know, had any conversation about that, apart from saying, you know, about being on the paracetamol? Have there been any other conversations?<br>R: No, not really. They keep, you know, they come in the night and say, do you want some paracetamol and anti-sickness? I have had some problems keeping, well, what little bit of food I could have, but that seems to be dissipating now so I'm refusing it. But that's it really, I've got one of these drip machines next to me and I think there's something connected but I don't know what it's doing.<br>I: Oh, right. So you don't know what that's doing, okay.<br>R: No. **[Patient08_L2]**<br>In terms of the medication, that was all transferred over satisfactorily. And she was taking everything that I expected her to be taking. I'm not a hundred per cent sure they gave her one of her multivitamins one day, but that's neither here nor there. And they seemed to have discontinued the ondansetron, which she'd been taking before she went in. But I think that was probably…well I asked her and it was unnecessary 'cause she hadn't any nausea. But there was something introduced in to her medication which I was not aware of 'til I saw it and they seemed to have put her on a lansoprazole 30 milligram which I thought I recognised but I didn't know what it was. I think they told her what it was or when I asked they told me, so that was a stomach protection. I don't know when she started that, whether it was in the HDU ward and they just carried it forward and I just didn't know **[Family16_L2]**<br><br>And I remember them saying things like, this is your pain relief, or, this is your antibiotic.[…] But specific names would have been completely wasted on me […] So I was being pumped with so many different chemicals to try and keep me going […] My blood pressure was higher than normal, so I were being given…on top of…I mean, I took blood pressure tablets to start with from home, but obviously they'd taken control of them. So I think I was taking an extra blood pressure tablet, or blood pressure medicine, to try and get my blood pressure down […] And, like I say, on top of that probably two to three different types of pain relief, sometimes they'd get injected, sometimes it would come in as an intravenous, sometimes it would be given as a drink, you know what I mean. **[Family19_L3]**<br><br>And the name of the medicine was never mentioned, the severity of the clot was never particularly mentioned. I know it was a life threatening one, but how life threatening, don't know. So the only medicine I know that he was given at that time was thrombolysis, which we didn't find out until we'd come from resus to critical care and then to the respiratory ward. Other than that, when they originally went onto the critical care, they were having what they called clot busting medicine, is what they called it in the critical care. And then there were a couple of other medicines which they never really mentioned the names of. I did ask and they just vaguely said oh, this is for so and so, this is for so and so. **[Family20_L2]**<br><br>Yeah, they gave him an inhaler, I'd say two or three days into being in the respiratory because he was suffering with a bit of breathlessness when he'd walked to the toilet and walked back. A bit of… The painkillers, when he actually mentioned the pain he was getting in the side, which is from the dead tissue from where they clot restricted the area in the lungs, they then started giving him the regular pain medication. He's now also, I forgot to mention, some laxatives… Because obviously the Co-codamol or the Paracetamol and Codeine can cause constipation. So they're given him laxatives he has three times a day. Changes wise, there's been quite a bit of change going from critical care up to the respiratory ward because obviously he doesn't have thrombolysis anymore, he doesn't have the injections anymore. He now has a tablet form of blood thinner, his painkillers, his laxatives and his inhaler, and his indigestion stuff.<br>I: So you know why those medicines were changed then, you're aware of why those…

		R: I'm assuming the medicines have changed because…the blood thinning because he was progressing well, there was no clot there for him to keep the blood thinners running through his body. The pain, obviously his painkillers, the laxatives because Codeine has a tendency to back you up and the Gaviscon stuff for his indigestion. So I do know why they have been prescribed, yes. **[Family20_L2]**<br><br>R: I think they did review the medication, but I don't know as anything changed. She came off most things. I think she was still taking paracetamol. I do remember the nurse coming and bringing her some tablets at one point, but I've got to be honest, I can't remember what they were now. […] I think some of it was paracetamol, because she's useless at taking paracetamol, and I remember the faces she pulled. Yeah, some of it was paracetamol, but I think there was another one as well, but I can't remember what, I think that was a heart tablet, possibly, but I'm not 100 per cent certain […] But by that time, she was able to think in the moment, and make her own decisions, she was much more compos mentis, even though her memory was gone from the day before. So she can't remember what happened yesterday, but in the moment she's fully with it, and can have a conversation with the nurse and make decisions for herself. So I just accepted that that was the medication they'd discussed. **[Family21_L3]**<br><br>I: But you do know what they are?<br>R: No, I don't know, I know there's a little heart-shaped one, there's couple of capsules, and that's about it. I don't know what they are. I'm pretty good…I: But you are…R: …with medicines as well, but I don't know. Nobody's actually come round and sat down and said, you're on this tablet, this tablet, this tablet, and this is why, nobody has done that. **[Patient22_L3]**
		I: So, what have they said about those medicines now, have they told you anything about those?<br>R: I've basically been advised to avoid them, so I'm going to strictly avoid them to ensure my liver's safety.<br>I: Right, okay, so you're not going to be taking those again, they're not going to be prescribed to you anymore, these other medicines, is that the case?<br>R: Yes. **[Patient06_L2]**<br><br>R: It's mostly been advice not to take them, but by choice I'm not going to take them, there is no point taking something if it's not going to work, that's just going to put my liver at further risk.<br>I: Yes, precisely, absolutely so then you've made that decision then?<br>R: Me and my mum together.<br>I: Right, okay, that's good and…<br>R: I was also prescribed them for anxiety, not depression and they don't really take the anxiety away. **[Patient06_L2]**<br><br>Yeah, so I said…well, I asked about what kind of drugs I needed to be on and they said that because I'd had all my procedures now it was just about keeping on top of the pain. And so I was very much involved in what pain relief I've had. I just stuck to what I was having, so paracetamol and I want to say Oxycontin, which is a bit stronger. They keep checking on me and making sure that that's doing the job. And if I'm honest it's not taking the pain away completely because I've got a broken clavicle, so that's really quite painful. But it's enough pain relief that I'm able to sleep and enough pain relief that I can still feel my injury, so I know that I'm not doing too much in the daytime, because for me I wouldn't want to kind of be in no pain whatsoever and carry on with my day and not realise that I'm doing further injury to myself. I think together I feel like I've been involved in the decision, we've come up with a really good balance that I can sit pain free, I can do most things pain free, but I know when come to use this right clavicle, I know my limitations and I think that's a good thing because I don't want to be doing any further damage. **[Patient24_L3]**

	**Decisions about medications and medication plan**	R: They start today. I spoke to the doctor. I got told on…what are we…we're on Thursday now, aren't we? I were told on the Tuesday that I could come home on the Wednesday. The doctor came and he organised a letter for my local practitioner, he's arranged for the nurses to come in, he's arranged for…and give me this injection.<br>I: Right, so the nurses are going to come and do that.<br>R: He's organised everything for me. **[Patient01_L2]**<br><br>I honestly, I think if I'd have turned round and said I don't want the tramadol, you know, the effects that it has, like, and they are saying it's the…advising me it's the best they can do, but I certainly feel if I'd have said I don't want it, they wouldn't have, like, tried to railroad me, they'd have given me something else […] I was quite prepared to accept their professionalism and take it, you know, and get it, sort of, this [once, like, you know 00:12:16] and get there. **[Patient01_L2]**<br><br>R: It's mostly been advice not to take them, but by choice I'm not going to take them, there is no point taking something if it's not going to work, that's just going to put my liver at further risk.<br>I: Yes, precisely, absolutely so then you've made that decision then?<br>R: Me and my mum together.<br>I: Right, okay, that's good and…<br>R: I was also prescribed them for anxiety, not depression and they don't really take the anxiety away. **[Patient06_L2]**<br><br>R: And my water tablets. They've given me some extra water tablets, they gave me some more 'cause I've got so much water in my chest which I'm not supposed to have. That's for my heart and they're changing this, they're changing that and it's just getting a bit over the, too much for me [voices overlap 06:19].<br>I: Okay. So, you, they have been changed but you haven't, you don't really know why or the reasons for them? Do you understand any of the reasons why these medicines have been changed?<br>R: I understand some of them but I'm not 100 per cent sure and at this moment in time, I just want to listen to them, become better and go home. **[Patient07_L2]**<br><br>R: Well, in the end I had to simply accept that, you know, I think someone did mention that there was, and since I've realised, somebody somewhere has said that there was a distinct possibility if you go too fast, you get a really… with lowering the blood pressure again, you really tank it. […] And your body is not…what are we doing, and that's the rationale for doing it gradually, I think. So, I accepted that at the end of the day and just went with their advice. **[Patient12_L2]**<br><br>I don't really think so, but again, I don't really think there's been much need for me to be involved, because like I said before, you just put trust in people that know what they're doing. Anything we've asked, we've always been told, but decision-making, the nurses and doctors are just giving her what she needs, I suppose. They've not really said, is it okay if she has this, is it okay if she has that? Because obviously I'd have just said, yeah, whatever you think is best. I'm no doctor, I'm no nurse, so yeah. **[Family15_L3]**<br><br>I: Yeah, that's good. And has that, I mean, with your dad, has that continued now that he's on the ward in terms of the way they're talking to him and keeping him informed of what's going on since, as it were?

		R: Well, it's funny you should say that because while we were there yesterday, they came to give him his injection into his tummy, you know, the anti, to stop you from getting a blood clot. And the nurse came over to him and said, this is the, she did say the name and what it was for. And she said, are you happy to have this, and he said, yeah. You know, [inaudible 00:19:23] happy to have this injection? And he said yes. From that I take it that they are, you know, he said that they're bringing him his medicines and they're all right. And he would check, my dad, because he's that type. That type, he'll make sure himself that he's getting what he should be on. **[Family23_L2]**
		I: Yeah, good. And thinking about the decisions made around your medicines do you think you've been involved in the decision making processes, or included in it?<br>R: I think I was included in it because what they were saying was, particularly around the painkilling, you know, do you want to continue to have, say, codeine, or whatever, and if I'd said, no, I don't want that, then, no, you didn't have to have it, you know, we would continue with the…with something else in its place. So, in that way, yes. In terms of, well you need to have painkillers, you know, there isn't a sort of discussion about that, no. **[Patient04_L2]**<br><br>R: This time with the, it seems to be better than I was in before. Because the doctors come round in the morning and they'll come and they'll actually talk to you. And they'll talk to you and say, well, this is what we want to do. We want you out by this much warning. See, this, a doctor might then want to see this. I didn't have this before. If that makes any sense?<br>I: Yeah, yeah. And what do you feel about that? Do you think that's good?<br>R: That. Yeah, yeah, it is good, yeah. It seems to be better. It gives you the confidence because, well, hold on a minute, they know what they're going to do. They've started to bring me into the conversation with themselves and that's it. **[Patient07_L2]**<br><br>R: I think while I was on intensive care everything was explained telling me that, you know, we've got to do things differently, obviously intravenously rather than tablet form, so I think that was all basically explained quite well.<br>I: Right. And did you get a chance in that to give your opinion, or did you want to give your opinion or do you want to be involved in those decisions or did you just leave it to them?<br>R: I was involved because they did give me the opportunity to ask any questions or queries if I wasn't sure, which I did on a couple of occasions, but I was quite happy with what they were telling me. **[Patient10_L2]**
		I: Right, okay. So I suppose to follow that up then, do you, you know, things have been explained to you but you feel that you don't want to be included in that, is it, you know..,?<br>R: Oh, if they...I: Yeah.<br>R: No, no, if they asked me, I would give my opinion, like I am with you, but they just seem to come and say, do you want some anti-sickness, do you want some IV paracetamol? We're just going to top up your fluids, like, and I just accept it.<br>**[Patient08_L2]**<br><br>I: The other thing is, or a couple of things. One of those is, thinking about the decisions around your mum's medicines, do you feel you've been included and involved in that decision-making?<br>R: I don't really think so, but again, I don't really think there's been much need for me to be involved, because like I said before, you just put trust in people that know what they're doing. Anything we've asked, we've always been told, but decision-making, the nurses and doctors are just giving her what she needs, I suppose. They've not really said, is it okay if she has this, is it okay if she has that? Because obviously I'd have just said, yeah, whatever you think is best. I'm no doctor, I'm no nurse, so yeah. **[Family15_L3]**

		So they did give us information what they would be stopping and how they would stop it and what they would do again to re-sedate him sort of thing. So I think there was the information but I don’t think it was very… I think they just do it, basically. Obviously I was of the understanding that that’s just what they do, they do whatever they think. If they did the blood test and the infection markers were showing something deranged still, they need to change an antibiotic, they’d just go ahead and change it. When a conversation, I think it was more just they do what they do to sort him out, basically.<br>I: Yeah. So they react to the situation and then maybe…<br>R: Acutely, yeah.<br>I: Yeah. And then explain afterwards. I think, as you say, because it’s that acute environment, isn’t it, maybe? **[Family18_L3]**<br><br>R: No, no, they've not given us any indication of the plan going forward. We literally get it day by day and that's if we're lucky enough to get the sister on the ward because the sister will explain it. If the sister is there, she will sit down and explain it. If the sister's not there, they'll literally explain to you what they're doing in that moment and they can't give you anything more than that. They just say, the consultants know what they're doing. **[Family25_L3]**<br><br>I: Firstly, do you feel you want to be involved in those decisions? And secondly, have you been involved in those decisions, if you like?<br>R: Well, I was uninvolved in the decisions. […] It's just, it might be, that I'm being too apathetic and saying, you know, leave me alone, just get on with it, I don't know. **[Patient28_L3]**<br><br>I think a lot of the decisions haven't really included us, or like me and my father, sorry, as such, really. It's more, especially on the critical care unit, it's like, as I touched on earlier, it was very much said, this is probably what's going to happen, and these are the medications, and gave us a bit of information about it. But with the nature of the unit itself, I think there's, kind of, the expectation of, with it being such a serious scenario, they're more giving you, like, this is probably what we're going to give her. Rather than being included, and you're deciding, or helping us decide. **[Family29_L3]**<br><br>I: Yeah, I do feel for you. You said there’s movement beyond renal and they’re going to try and get physiotherapy for him, but in terms of medicines, do you know if there’s a plan of what they’re going to do with his medicines?<br>R: I don’t know what they’re planning to go and do with his medicine, no. There’s a team trying to get things in place for him and how quick that goes, it depends as soon as a vacancy comes available, really.<br>I: Right. And this is the next step, is it, beyond…<br>R: Yeah, this is the next step for him is that he will be moved out of renal care and he will be going to what they call rehabilitation care. **[Family30_L3]**
		I: Right, okay, so it’s about when you get the information leaflet, they want you to look at that carefully and think about that. Do you feel that you’ve been involved in some of these decisions, say you’ve talked with your mum, and you’ve talked with the doctors, how do you feel included and involved in what’s happening with those?<br>R: Well, everyone has always spoken to me about everything that is happening around me and always made sure that I was aware of it, even in a confused state and that way, I was able to make executive decisions with them, but I wanted them to make them with me because of the confused state, if I’m honest.<br>I: Right, okay, that’s really helpful and so you feel that there’s been a discussion, you feel that…you said you feel you’ve been included in all the decision-making, has that been important to you then?<br>R: Yes, I like having a sense of control.

		I: Okay, so do you think it's important to have a sense of control around your medicines you're taking, is that because of what has happened to you?<br>R: Yes, because it shocked me so much when I got carried away, I'm way more strict on that side of things now. **[Patient06_L2]**<br><br>R: And there was also a conversation, a three-way conversation, no…a four way conversation between myself, the upper GI support nurse, the junior doctor and the consultant on the day I was due to go out, and it was a question as to whether I…as far as the consultant was concerned, I was medically fit to discharge. The support nurse, I think, thought that I should stay another night and try and get this oedema sorted out a bit more and be happier before I went home. I wish I'd taken his advice, but in the end I went with the consultant saying, you know, there's no real reason for you to be here and the rest as they say is history. I wished I had stayed because I think the junior doctor was talking about, you know, putting a drip up and getting some diuretics into me, even there and then, but that's history and 28 hours in Northern General is not much fun I can tell you, especially when you spend nine hours lying on a trolley in A&E.<br>I: Right, so that's what happened when you went back?<br>R: Yes. **[Patient12_L2]**<br><br>I asked about what kind of drugs I needed to be on and they said that because I'd had all my procedures now it was just about keeping on top of the pain. And so I was very much involved in what pain relief I've had. I just stuck to what I was having, so paracetamol and I want to say Oxycontin, which is a bit stronger. They keep checking on me and making sure that that's doing the job. And if I'm honest it's not taking the pain away completely because I've got a broken clavicle, so that's really quite painful. But it's enough pain relief that I'm able to sleep and enough pain relief that I can still feel my injury, so I know that I'm not doing too much in the daytime, because for me I wouldn't want to kind of be in no pain whatsoever and carry on with my day and not realise that I'm doing further injury to myself. I think together I feel like I've been involved in the decision, we've come up with a really good balance that I can sit pain free, I can do most things pain free, but I know when come to use this right clavicle, I know my limitations and I think that's a good thing because I don't want to be doing any further damage. **[Patient24_L3]**<br><br>So, everything's liquid form now. But, yeah, it feels like a choice, like it's a, you must take this. This is what it is…I also have an injection in my tummy once a day as well, I think that's a blood thinner because I'm not moving around as much. So, that's an option. There's also an option to have…oh what do you call them when you have the face masks with the…a ventilator, essentially like an inhaler. That's also my decision but they always try and give it me when I've got visitors, so I didn't have that for the first few days. But that was to clear stuff off my chest and every day I feel my voice is stronger. So I had this conversation with the nurses and I think they all tend to agree with me and we'll kind of like come to an agreement that it's not really necessary. So, yeah. **[Patient24_L3]**<br><br>R: Yeah, I feel like everybody's…we've all got one goal in mind and that's to get us better. They will listen if I've got concerns, if they don't have the answer they'll go and find somebody that does. Yeah, it's been a breath of fresh of air being on the ward. Yeah, everybody's very respectful, very mindful that you are an individual with feelings and thoughts. And if you've got questions and, like I say, if they don't have the answers they'll always find out for you. **[Patient24_L3]**<br><br>Yeah. (name)'s done the most part, he made the choice for the operation, he was conscious at that point. While (name) was not able to voice, his wife was his next of kin, so she took the control of the decisions. And as soon as (name)'s been conscious again, the decisions have fallen back to him. They have been good at, like, asking (name) for that and letting (name) make the choices on when he feels ready and things like that, you know, I will give them their dues in that, they have. **[Family25_L3]**
		I: Where has that support come from?

		R: Well, everyone, the doctors, my parents, my life partner. **[Patient06_L2]**<br><br>R: Well you're definitely not in the early stages are you when you're in the HDU or whatever it is, but as for the transfer between the epidural and everything else, I felt really hundred percent that I was given the option basically, you know, there was this thing called Tramadol and I…[inaudible 0.18.55] but I think I'd rather avoid it if I possibly could, you know, that [inaudible 0.18.59]. I've still come home with some oramorph that's, you know, I think that's par for the course isn't it, that's a standard thing. I mean, I haven't used it, I don't suppose I will use it. […] But I'm sticking with codeine and we had that complete conversation and I was quite happy with the result. Just codeine and paracetamol. **[Patient12_L2]**<br><br>And I wouldn't really expect them to have to discuss everything with us. For instance …because he was on a CPAP, so he was on like an oxygen hood, because he got really bad pneumonia which affected his lungs really badly so he was struggling breathing, when it was a conversation about putting him on a ventilator that was a very informed conversation. It wasn't they're just going to go ahead and do it, it was what do we think as a family about the idea of him going on a ventilator, and they explained it in a lot of detail, and they gave us time to have a think as a family what we thought would be the best option for him given the information that they told us. So I thought that was nice they actually included us in that. **[Family18_L3]**
	**Information exchange - flows between patient and health professionals**	The only problem I did have, and I had a bit of a fall out over it, when I went in for my pre-med, I had to take in a list of my medicines. Well, I take metformin for type 2 diabetes, I take a statin I've just started taking on recommendation, not because I need it but because of my age, I'm 69. It's to do with, like, coronary heart attacks, all of this lot. And the other one is an anti-reflux tablet which I take. Now, when I went in, I got a letter from the people. I took it into hospital with me. Now, all it had on there was do not take the metformin, only one that day. When I was in there, they were coming out and taking my sugar, they wouldn't give me metformin. And I asked why. And so they said, well, the doctor said so. I said, well, the doctor's not told me that, and I said, I want to know why. They said, we can't tell you. Well, I said, I'll be quite honest with you, the way I feel at the present time is, it's pointless me having my sugar being taken three times a day when whatever reading it is… Once I was 12.9. I says, it's quite pointless me having this procedure… Anyway, they did come back, talked to me, and they put me on it. **[Patient01_L2]**<br><br>R: One thing that I do notice is that when they do do their rounds, she likes me to be there and fortunately I have been there because they do their rounds in the morning. This is only what happens on this ward and they tend to be a team of people that come. So you've got your main consultant and you've got presumably your trainee doctors and so they all kind of like arrive on troupe because they're kind of like learning and she almost panics and says to me, (name) you will stay in the room. I think it's just…my mum's always been very fiercely independent, she's a retired teacher and she's very articulate and things like that, I think she just finds it a little bit overwhelming now and wants me to be there to listen to possibly ask the questions, you know. […] Yes, because they almost look when I…because initially they'll ask me to go out because there is a bay of four people and they obviously don't want me to hear what's being said for the others but I have to ask, well, my mum has to ask, and some of them almost look at me like it's an inconvenience, why would you possibly want to come back in? I'm thinking, well, actually, it's not me, my mum's just said to you, she wants me in. So it's not me being difficult, my mum says, can my daughter be in? It's not almost…I would have expected it to be, oh, yes, of course, and it's almost like, oh, well, yes, she could be. I thought, well, it's not quite… **[Family11_L3]**

		And there was also a conversation, a three-way conversation, no…a four way conversation between myself, the upper GI support nurse, the junior doctor and the consultant on the day I was due to go out, and it was a question as to whether I…as far as the consultant was concerned, I was medically fit to discharge. The support nurse, I think, thought that I should stay another night and try and get this oedema sorted out a bit more and be happier before I went home. I wish I'd taken his advice, but in the end I went with the consultant saying, you know, there's no real reason for you to be here and the rest as they say is history. I wished I had stayed because I think the junior doctor was talking about, you know, putting a drip up and getting some diuretics into me, even there and then, but that's history and 28 hours in Northern General is not much fun I can tell you, especially when you spend nine hours lying on a trolley in A&E. **[Patient12_L2]**<br><br>so I could actually recognise tablets just by looking at them, mostly, but not always of course. I noticed the ondansetron was missing. There was no conversation with me about why that was taken away. But I could see the logic for taking it away. I don't know whether they'd had a discussion with (name of patient/participant's spouse) about it, possible, possibly not, I don't know. **[Family16_L2]**<br><br>I: Right, okay. But was there any…<br>R: Nobody, there was no proper update, they just said they were pleased with him, and he could move onto a ward, that's all they said.<br>I: Right, so there was no conversation about anything that was going to be happening with the medicines, or anything like that, at that particular point?<br>R: No. **[Family17_L2]**<br><br>I: And do you think you're getting enough information then as a consequence?<br>R: You never get the full picture with doctors unfortunately. They can be very vague. But you have to ask the right questions otherwise they don't answer properly, which I have found. And there has to be more than one of you because obviously two people have got different points of view, different questions, different sides of questions to ask. So it's easier when there's a couple of you asking questions.<br>I: So you think that's a…yeah, that reluctance is because they think you won't understand or because they are being guarded?<br>R: Possibly. I definitely think a bit more transparency when it comes to these things is needed. I would certainly say that. **[Family20_L2]**<br><br>R: To be honest with you, I haven't had any conversations around any medication.<br>I: Right, okay.<br>R: Because I couldn't even…I know I'm on a heart tablet and I know I'm on a stomach tablet and I know I'm on a painkiller, but that's about it. **[Patient22_L3]**<br><br>R: [Brief interruption]. Yeah, I've not actually sat down and had a conversation with anybody regarding medication, because before I came in to hospital before I had my arrest, I was on Duloxetine, on an antidepressant of 60 mg a day, and I've been asking for the last two days where they are, and apparently, they've been stopped. We've only found that out because (name of nurse) asked them today for me.<br>I: Right, okay. So you've had medicines beforehand that you're on, this antidepressant […and that's been stopped you said, but no one's come to you and talked to you and discussed with you about why that's been stopped, have they? **[Patient22_L3]**

		R: I mean, it's been a bit tricky, they've not actually been that great at communicating unless we've been pushing them for information, to be honest. So they are very good at coming in and saying to (name), we have, for example, your blood thinner to give you, we're just going to do it in your right arm, as such. Not necessarily, (name), can we do it in your right arm? They will tell us what it is and things like that. However, most of the time, they don't really explain what it's for. […] Yeah, so they might say, I don't, (name), we've got your antibiotics but they don't specifically say these…or haven't specifically said that the antibiotics are for general infection, protection against infection, whether he is currently fighting an infection, you know, it's been a little bit like that. The pain relief…in terms of like, pain relief and medication on that side of things. We had a bit of an issue when he first went from the ITU down to the ward, where (name) had no pain relief, they'd not had the doctors re-prescribe it from the different system. So (name) went almost…I think it was about 15 hours without anything and he was in agony, absolute agony. And when we were asking and asking and asking regarding where is his medication and why has this occurred, all we got was, I don't know. **[Family25_L3]**<br><br>R: Yes. So I think the way that it was explained is that they have a different system in the ITU. So once (name) was moved from one ward to the other, it all needed re-prescribing onto this new system, but it hadn't been done before we left the ITU. However, nobody communicated that with us and I'm assuming between themselves as well, until (name)'s pain relief had ran out from the one that he currently went down with. And it was only when (name) started to experience pain and we asked what pain relief had he got and they said he'd not been prescribed any. So they were unable to give him anything, they couldn't even give him paracetamol […] And that took several hours it took for me to…and, you know, I'm not one for shouting unless I really have to but I really blew my top, to be honest with you. It got to a point where we'd asked several times, (name) had been so patient. He was, at this point, throwing up, he was in absolute agony. All the progress that he'd made in the ITU had gone. He'd, at one point, asked to switch the machines off and said I don't want to be here, I can't do it, he was in that much agony. And as a mother, to hear your son say that, as you can imagine, that was the point that I blew my top and I demanded to see the consultant. And finally, after hours and hours and hours of waiting, they finally prescribed him…the first thing was paracetamol but then got him something stronger, which was they put him on…I believe was oxycodone which, it was the button that was…and then that started making him sick. **[Family25_L3]**<br><br>I: When you talked about having to go to the consultant and be fairly forceful, as it were, you know, what was that like, what did you feel about having to do that?<br>R: To be fair, it was a mixed emotion, if I'm being honest. You know, inside I'm broken because my son is in agony and there is nothing that I can do. I'm angry because I know there's people that can do something. I know that they're stretched and I understand and totally appreciate that, however, I just never felt that they were on our side. So it always felt like a bit of a fight. I had a consultant's words to me, when I…because to me, they weren't checking (name), they weren't…I'm very clued up on healthcare, so I work in the healthcare industry. So, you know, they'd done no pressure care with (name), they'd not come in to check for anything. His feet were overhanging off the bottom of the bed, they'd not offered to take him up the bed. There were so many things going on that it just built up over this time period of (name) being in the state that he was in. I said to the consultant, it's institutional abuse, which it was. And he said, I completely agree. And that to me was just like the icing on the cake because then he said, however, we are short staffed. But, you know, for me, how is that the patient's fault? Not just my son, I don't doubt other patients are probably in the same boat, however, you know, it shouldn't be that way for anybody. So it was such a mixed emotion, to be honest. It's quite heartbreaking as a mother to watch your son in that much pain to the point that you feel like you have to blow your top just to be felt like you've been heard. **[Family25_L3]**<br><br>No, no, they've not given us any indication of the plan going forward. We literally get it day by day and that's if we're lucky enough to get the sister on the ward because the sister will explain it. If the sister is there, she will sit down and explain it. If the sister's not there, they'll literally explain to you what they're doing in that moment and they can't give you anything more than that. They just say, the consultants know what they're doing.<br>**[Family25_L3]**

		R: Yeah. We have, yeah. I mean, we go through everything so thoroughly anyway, it really is about taking…for me, I just always say take your time, even if it's an urgent decision, take your time. (name)'s made decisions throughout this whole process and come through to the ward and now does not remember making decisions. So we've had to reapproach them, you know. So everything that we do now, we, sort of, go through with a fine toothcomb. In terms of giving them the feedback, no one's ever really asked us anything about any of it, to be honest, other than just general, oh, was that okay, does that dressing look alright, okay, do you want anything else? No one's ever sat down and said, can I just ask how you're feeling, you know, how do you feel about making that decision, do you feel like you understand that decision? No one's ever done any of that. **[Family25_L3]**<br><br>So, she knows that she will have to be on loperamide again. She knows that she will have to, at least, have pain relief. But we haven't really discussed, like, what's going forward. It's more like, based off the initial assumptions of, this is what happened last time, this is, kind of, the standard of what's going to happen, or from the assumptions we can make, we're just seeing that, oh yes, she's on loperamide again, she's taking her inhalers again. And it's more, we're making just assumptions based on the information we've already had.<br>**[Family29_L3]**<br><br>R: But from what's been relayed from my mum, it seems very much, a lot of things end up getting debated a lot, and it just, I think, when you have such a mixture of people, with opposing outcomes, we're going to call it. Because, like obviously, everyone is wanting what their speciality is for, to come to the correct outcome. […] But sometimes, that obviously impacts what the outcome is of somebody else's speciality. And I think because of that, it just, I wouldn't say I'm particularly personally coordinating it, but I know that my mum sometimes does have issues in knowing exactly what she's meant to be doing. **[Family29_L3]**
		Just basically that, you know, it was for…they were for…they were the ones that I'd been prescribed to take and that it was okay now to actually have those. There were some iron tablets that I was also taking and I said, am I taking those? And they said, no, at this point we're not doing…we're not going to give you the iron tablets while you're actually in here and they will restart afterwards, but for the time being, no, we're not doing those. **[Patient04_L2]**<br><br>I: Right, okay. So in terms of then, you know, what's happening now on the ward, has anyone come to talk to you about medicines, you know, had any conversation about that, apart from saying, you know, about being on the paracetamol? Have there been any other conversations?<br>R: No, not really. They keep, you know, they come in the night and say, do you want some paracetamol and anti-sickness? I have had some problems keeping, well, what little bit of food I could have, but that seems to be dissipating now so I'm refusing it. But that's it really, I've got one of these drip machines next to me and I think there's something connected but I don't know what it's doing.<br>I: Oh, right. So you don't know what that's doing, okay.<br>R: No. **[Patient08_L2]**<br><br>Yes, and if I was there when the doctor was doing his round he'd say to me, have you got any questions, and things like that and that was fine but I wouldn't…and I guess, I didn't specifically ask anything about medication or anything specifically on those lines. **[Family11_L3]**<br><br>And they were brilliant, they explained everything, how everything had gone. Yeah, I can't honestly remember all the specific details as to what… With regards to what medicines she was on and stuff like that. I know she was on a PICC line and she'd been on that before her surgery, before Critical Care, she was still on that for a while after Critical Care because she couldn't eat, still. That got explained to us again. I mean, we already knew but they took the time to explain it all to us again. Yeah, they were really good. **[Family15_L3]**

		Well when we first get there my dad's chances weren't very good. So we were actually pulled into a room, well we were taken into a room, by the consultant and she explained what they were doing in detail. What they were going to try and do, what medications they were going to try and give him to try to stop him getting a chest infection, you know, the antibiotics and everything. She explained all that and she...and then, when he was in critical care, that was straight away, that was as soon as he was transferred to critical care, and then, from then on, we went every day. Me, my sister and my brother went every day and as questions came up, they answered us, they answered them. But they'd already been through the fact that, you know, what they were trying to achieve, you know, in critical [care 00:06:55]. But to be quite honest, they saved his life, to cut a long story short. Critical care saved his life without, you know, without any doubt. **[Family23_L2]**
		R: They were just talking about sorting out the medication before I leave and telling my mum what's going on with it.<br>I: Right, okay, so they talked to your mother as well, as well as you?<br>R: Yes.<br>I: And did you have to ask any questions around that, or did they come to you and say, what that involved?<br>R: Well, they kind of just said it straight up, but then my mum had concerns about it affecting my liver. So, she delved a bit deeper into that side of it and just straight-up asked the question. **[Patient06_L2]**<br><br>R: They were just talking about sorting out the medication before I leave and telling my mum what's going on with it. **[Patient06_L2]**<br><br>I: Right, okay, and in terms of that conversation then, around that, or at that time, who was it who talked to you?<br>R: I would say a lot of that was explained by a sister that was doing…when Mum was really poorly and she was, kind of like, working with her constantly there was a sister that worked with her and she explained that to us in quite some detail.<br>I: So it was basically around nursing staff then who would do that.<br>R: Yes, I would say most of my conversations were with the nursing staff because they were the ones that were doing the main care for her. I mean, had I requested a conversation with a doctor that would have been…you know, that was possible but I found that I was happy with the information that I was getting from the nursing staff, particularly this…this particular sister was really good.<br>**[Family11_L3]**<br><br>R: I think it's because what happens is the doctors do their rounds but, to be honest, I guess the thing that interested me as family was, how is my mum on the…and they were the ones that were caring for her on the 12 hour shifts so I was talking to them about her…how she'd been in the night time and how she'd been in the day time. I guess that was more…and I guess I trusted the drugs and med… Had Mum obviously not been responding to treatment or I thought that the sister's answers weren't fulfilling what I wanted to know, then I would have gone to a doctor but I didn't feel that I needed to, if that makes sense. **[Family11_L3]**<br><br>R: Yes, and if I was there when the doctor was doing his round he'd say to me, have you got any questions, and things like that and that was fine but I wouldn't…and I guess, I didn't specifically ask anything about medication or anything specifically on those lines **[Family11_L3]**<br><br>The nursing staff in Critical Care, they informed. Everything we asked, they had an answer, or they'd try and find out the answer. And since she's been back on the ward, just everybody involved, so all the nurses and… We spoke to…well, I've not, they've been there, but my mum's spoke to the physiotherapist and the dietitian, just to talk her through about getting a bit of exercise and saying it's important to sit up every so often rather than just lying in bed. Trying to get her to walk around a bit if she can. Oh yeah, another thing that they did, she had an epidural for the surgery and that got taken out and she did start to feel the pain quite a bit, so then she went on some other medication for pain. I think it might have been codeine. That's something that happened on the ward that we got told. **[Family15_L3]**

		They did ask…they rang me one evening and asked for my help to talk to her because she was…this was when she was in the HDU, talk to her because she was getting paranoid delusions, so…but they hoped I could calm her down. But I actually found it quite difficult because she…it was full blown. And I offered to go in and…this was about ten o'clock at night, I offered to go in and speak to her in person, but they said they'd ring me back if that was necessary. Anyway, they rang me back at eleven…and said, she'd calmed down, she'd got to sleep and then they'd reduced her dose…I can't quite remember what she was on at that time, the infusion level down. And then she was okay. I mean, I did know what it was at the time but…and I'd not had a lot of sleep myself for the last two days, so…but I am beginning to get things [voices overlap 27:00]… **[Family16_L2]**<br><br>**R:** But the staff in the intensive care unit were very, very helpful and tried to explain, like this is for blood pressure, this is for this. So they were really good in explaining stuff.<br>I: Right. Okay. So you spoke about the staff in ICU, was it particularly those staff that talked to you or were there other staff?<br>R: So it was mainly the nurses, because obviously when a patient's in ICU, they have one to one care, and we got to know them quite well, because my partner was in intensive care for three weeks. But the consultants used to call… **[Family18_L3]**<br><br>R: I think, to be fair, the nurses on the intensive care unit were very explanatory, but I don't know if that is what they're normally like or if that's just because, like I said, I have a bit of an understanding I would ask a lot of questions, so what is this for and why are we giving him this, is this a long term thing, will this affect this, is this good for…so I used to ask a lot of questions. So I'd ask all the blood results, I would ask about his medicine. So if I went one day and there was new medicine added I'd ask what that was for. And before even the nurses had told me in the morning, the first thing I did I would look at the syringe drivers and I'd look at the milligrams and I'd see if any of them had changed because I used to remember them. So if I noticed, I don't know, his noradrenalin had been put down two points, that would be positive. She would say oh, that's good, the reason we've done it is because he's tolerated this more, his blood pressure hasn't gone back up. They would explain what they were doing and why they'd done it. But again, like I say, I don't know if that's just because I would ask a lot or that's what they'd usually do, I'm not too sure. From my experience, I do feel like they did explain it a lot to me. **[Family18_L3]**<br><br>But I think as a family when someone is in intensive care you are basically entrusting your loved one's life to them. I feel like you have the reassurance that although they are really critically ill they are in the best place possible and you entrust whatever they are doing is to help. So I think it wasn't really a discussion that they were doing this, but it would be… If, for instance, they planned to do a sedation hold, they'd say oh, tomorrow we're going to reduce his… I think it's Propofol, I think it's called, we're going to decrease that to try and see if he'll wake up a little bit. So they did give us information what they would be stopping and how they would stop it and what they would do again to re-sedate him sort of thing. So I think there was the information but I don't think it was very… I think they just do it, basically. Obviously I was of the understanding that that's just what they do, they do whatever they think. If they did the blood test and the infection markers were showing something deranged still, they need to change an antibiotic, they'd just go ahead and change it. […] And I wouldn't really expect them to have to discuss everything with us. For instance …because he was on a CPAP, so he was on like an oxygen hood, because he got really bad pneumonia which affected his lungs really badly so he was struggling breathing, when it was a conversation about putting him on a ventilator that was a very informed conversation. It wasn't they're just going to go ahead and do it, it was what do we think as a family about the idea of him going on a ventilator, and they explained it in a lot of detail, and they gave us time to have a think as a family what we thought would be the best option for him given the information that they told us. So I thought that was nice they actually included us in that. **[Family18_L3]**<br><br>And the physio was great, because they'd speak to all of us. Rather than just speak to my dad, they'd speak to all of us. The doctor not so much tried to speak directly to my dad, me and my brother or my partner have to actively involve ourselves in that conversation, ask some questions, which I don't mind doing. But I'd prefer to be told to me. So we know what's going off. Even though I think they already know that my dad's… his mental health state is not stable. So they should be talking to us anyway. **[Family20_L2]**

		I was going to say, on a couple of occasions they mentioned something that I didn't understand, you know, they mentioned a name of the medication. And I said, well what does that do? And they explained it, that was, well we're giving him that because of this. And even down to his oxygen, he had to go in, like an oxygen thing, like a bubble really, which he hated. And they explained to me, it was like a CPAP thing, and they explained to me why he had to be in that, because the oxygen levels, they were pumping the oxygen levels into his lungs. And then, gradually, as his lungs improved they took him off that and put him on different types of oxygen, and all that was explained as well. Because, you know, obviously, when you see my dad in, like, a plastic bubble, you want to know why he's in there. And it was all explained why, why he had to be there. And, you know, every question we asked they answered, if I wasn't sure if he was being given a medication for something that the consultant had mentioned, and I said, well what was that for? The critical care nurse explained it, what it was for. **[Family23_L2]**<br><br>I: Yeah. Who has, you know, thinking about that, can you talk me through any, sort of, conversations you've had with any health care staff about those?<br>R: Yes, of course. When I was in the critical care unit, in Northern General, I was able to talk every single day, since I was visiting normally around, like, the evening, kind of, time. But every time that I did go in, they'd always give me an update on what she was on, and just her health update, as well, on top of that […] I was always kept up to date with it. I know that, during the stay, it was quite, with the situation that she was in, there was quite a few different medications that were on and off, yeah. But every single time, they were putting her on something, or they were taking her off something, I was informed. And I was allowed to, like, just ask, any time, really. I was up to date with any progress that was made. As far as like the medication wise, and the general health wise, and I can't really say any more than that, because I mean, they were brilliant, I was just able to ask any time. **[Family29_L3]**
		Now, when I went in, I got a letter from the people. I took it into hospital with me. Now, all it had on there was do not take the metformin, only one that day. When I was in there, they were coming out and taking my sugar, they wouldn't give me metformin. And I asked why. And so they said, well, the doctor said so. I said, well, the doctor's not told me that, and I said, I want to know why. They said, we can't tell you. Well, I said, I'll be quite honest with you, the way I feel at the present time is, it's pointless me having my sugar being taken three times a day when whatever reading it is… Once I was 12.9. I says, it's quite pointless me having this procedure… Anyway, they did come back, talked to me, and they put me on it. **[Patient01_L2]**<br><br>R: Well basically when they came to give them me I said, you know, what is it you're giving me? So, they told me what it was and why they were doing it. And I said, well I just want to…you know, I'm not being nosey, I just want to know. And they said, no, it's your body, you're entitled to know what's going into it. So, they did tell me what they were giving me, yes. I can't remember what they were giving me but, yes, they did tell me what they were giving me and why they were giving it to me. **[Patient04_L2]**<br><br>R: They were just talking about sorting out the medication before I leave and telling my mum what's going on with it.<br>I: Right, okay, so they talked to your mother as well, as well as you?<br>R: Yes.<br>I: And did you have to ask any questions around that, or did they come to you and say, what that involved?<br>R: Well, they kind of just said it straight up, but then my mum had concerns about it affecting my liver. So, she delved a bit deeper into that side of it and just straight-up asked the question.<br>I: Right, okay, so your mum prompted and asked some questions about that, did she then?<br>**[Patient06_L2]**

		R: That's [inaudible 16:05]. No, it can be a two-way conversation if you want to talk. 'Cause if you want to talk, he'll talk to you, the doctor. But the consultants, all of them will talk to you and it's not just the doctor who comes, it's all the consultants as well. They'll wing in as well.<br>I: Right, okay. So, there is a, sort of, like, exchange of…R: Exchange of banter, if that makes some sense?<br>I: Yeah, yeah. I was going to say views, but banter's fine.<br>R: Views, yeah, you're right, views, yeah. **[Patient07_L2]**<br><br>I: Has there been any, sort of, well, I mean, actually, one of the things to think about is, were you on any medication before you went into hospital?<br>R: Nothing major, no, nothing major.<br>I: Right, okay. So, and is that, if you were, has any of that continued or has that been stopped?<br>R: Well, the only thing that I was on was Simvastatin, and that's a tablet and they said they can't give me a tablet when I'm being sick.<br>I: Yes, quite. So they've stopped that?<br>R: Yeah.<br>I: Right. And did you have a…were you, were you just told that's why and you went, okay, fine, or did you…?<br>R: Yeah, I did ask where my Simvastatin was because it's one of those regular night-time things for me. You know, I've been doing it since my mid-forties and the nurse very, very nicely explained, we can't give you a tablet while you're likely to bring it up, it'd just come straight back. **[Patient08_L2]**<br><br>R: I think while I was on intensive care everything was explained telling me that, you know, we've got to do things differently, obviously intravenously rather than tablet form, so I think that was all basically explained quite well.<br>I: Right. And did you get a chance in that to give your opinion, or did you want to give your opinion or do you want to be involved in those decisions or did you just leave it to them?<br>R: I was involved because they did give me the opportunity to ask any questions or queries if I wasn't sure, which I did on a couple of occasions, but I was quite happy with what they were telling me. **[Patient10_L2]**<br><br>I: Yes, okay. And did you have the opportunity to ask any questions or anything like that?<br>R: Yes, definitely. I remember her saying that.<br>I: Right, okay. And how were those questions answered, you know, was it satisfactory, was it good?<br>R: I felt quite happy, yes, I think we discussed the options on various opioids and what have you, and I decided definitely to go with the lower strength options, given the drawbacks of some of the stronger ones.<br>I: Right, okay. So, you would talk…so side effects and stuff like that were talked through to with you?<br>R: Yes, I kind of have got a bit of a…well, I was married to a nurse for 27 years, so I've got a bit of sort of medical knowledge, and I do know the facts anyway of… **[Patient12_L2]**<br><br>R: But I haven't seen a doctor since.<br>I: Right, okay. So, no one has come back to you and said, well you know…but obviously, you know, he's off critical care, and he's…<br>R: Oh, yeah. We do ask every time we go, if we see a medical staff, we do say, how's he doing, has he improved, and things like that. **[Family17_L2]**<br><br>R: They've not, like I said, they've not really told us what they're doing, unless we ask.<br>I: No, no.<br>R: We don't know if they've changed an antibiotic, or anything.

		I: Right, unless you ask them what's going on?<br>R: They haven't even told us the name of the antibiotic, they just said, he's on an antibiotic, and that's it. **[Family17_L2]**<br><br>I: Do you have to ask more questions, or have you had to ask more questions since he's been on the ward?<br>R: Yeah. I do ask, because when I visit him in the morning the first thing I do as soon as I see a nurse I'll ask about his CRP levels, his pain levels and your blood results and what's going on at the minute. That's the first thing that I ask usually. And, to be fair, they will tell me what the levels are. But again in intensive care when I used to go and ask a level they'd say oh, but it's been trending from this and this is a good incline, they'd give me a bit more information, whereas they'll just tell me a number, basically. So I think it's harder to get the information because you do have to look for it, you have to ask a lot. Like today, for instance, I've been asking…because basically he's been in intensive care for all this time and then he's been on the ward, but now he's only having IV antibiotics, he's looking after himself, he's got a lot steadier on his feet, he's eating and drinking fine, kidney function's a lot better. So he is a lot better. So it's just trying to find out what the plan is now. Because obviously he's been in hospital nearly a month now, he's desperate to go home. **[Family18_L3]**<br>I: And do you think you're getting enough information then as a consequence?<br>R: You never get the full picture with doctors unfortunately. They can be very vague. But you have to ask the right questions otherwise they don't answer properly, which I have found. And there has to be more than one of you because obviously two people have got different points of view, different questions, different sides of questions to ask. So it's easier when there's a couple of you asking questions.<br>I: So you think that's a…yeah, that reluctance is because they think you won't understand or because they are being guarded?<br>R: Possibly. I definitely think a bit more transparency when it comes to these things is needed. I would certainly say that. **[Family20_L2]**<br><br>I: And when you've been having these conversations with people, you said about the nurses there, and the Nursing Sister, have you initiated anything, or have they come to you?<br>R: I think it was a bit of both really. They've come to us at times, and then if I've seen the nurse sat at the desk and I've got a question, I would approach them.<br>I: Yeah, so, and have you been happy to do that, do you feel confident to do that?<br>R: Yeah. I'm happy to do that, I've had a lot of involvement with hospitals with our mum over the years, and my dad, so yeah, I'm quite happy to approach any member of staff and say, do you know what's happening, or, can you answer me this question?<br>I: And do they answer the questions, do they answer them…?<br>R: Generally, they do their best to answer the questions. Yeah, they've been great. **[Family21_L3]**<br><br>So I've got absolutely no complaints at all, every question my brother asked or my sister asked, it was all explained by the critical care nurse why they were doing it. We asked about his oxygen levels, were they improving and they explained all that. We could see on screens, numbers, and they even explained, because we weren't sure what were his oxygen levels and what was something else because they can be a bit complicated, those. **[Family23_L2]**<br><br>So in terms of his eating and things like that, you know, they've not been the best at communicating whether (name) can eat and drink. They stopped his fluids the other day. And she just went to walk out and said, oh, (name), we're going to stop fluids now and we'll see how we go. And she just, sort of, went to walk out and I had to stop her and say, can I ask, but why are we stopping the fluids and does this now mean that (name) can drink? You know, before that, you were telling him just enough to wet his mouth, is he now supposed to drink independently, is that the plan? And then what happens if (name) starts to be sick again, what's our next action then, can we plan for both positive and negative so we're prepared? And she said, oh, we'll have to look at that if (name) starts to be sick again. And in my head, I'm thinking, well, that means he's going to be sat there suffering, can we have something in place that's ready, so if it happens, we can act. **[Family25_L3]**

		So for example, the other day they were talking about his…well, it was his diet again. They said that (name) could potentially look at starting solids again soon. So I said, right, well, in terms of that, you know, how…what's the plan in terms of timeline and are we doing a little bit, are we doing breakfast and then skipping dinner and tea, is it, you know, breakfast, dinner and tea, what's the plan with it? And they were, like, we're just going to see what happens and, sort of, where (name)'s feeling. Which okay, I get that, he might not want something straightaway but he might want something in the afternoon, I can understand that. But then, you know, I'd say to them, in terms of, like, his nutrition between then, can I ask what the plan is regarding that? Because at this point, (name) had gone three days without any sort of nutrition. And yeah, she just said, oh, you don't have to worry about that, he can cope with just the fluids. And there was no real depth of explanation in that, that was pretty much what she said word for word. **[Family25_L3]**<br><br>I: That's good, that's really helpful, thank you for that. Did you initiate some of those conversations, you said you asked questions, did you start off the conversations, or did they, you know, come to you?<br>R: You see, sometimes it was a mixture. Because a lot of the time, I do, kind of, seek out answers, as and when, that's just the nature of me, I think. But, even when not prompted, they would at least give me a general overview of the medications she was on, and any progress that was made within that day, when I'd arrived. It was just, sometimes, I ended up going to ask myself, I think that's just, as I say, I think that's just me as well […] Because I think, from what I gathered, when I was going to the critical care unit, they would have just let me know anyway. It was just, sometimes, if I had a question, anyway, I'd just go up to them and ask, yeah, before they got to me, really. **[Family29_L3]**<br><br>You have to actively seek out and ask. Because I know, I am aware, with it being a general ward, it's not to, the only way I can really put it is, a bit more hands off. Or you end up, there's specific times that people do come to check in on you and give you the medication. But otherwise, a lot of it is down to the patient, and obviously, the patient's family, like me, to go and ask questions, and ask for things like, with my mum's stoma at the moment, I have obviously raised that she is having a few issues with it, like leakages. And due to the nature of her skin at the moment, the adhesive isn't sticking quite right. And a lot of it is having to just know when to go and ask a nurse about it. **IN029_anonymised**
	**Information provision from health professionals to patient**	I: And you said that you sort of asked questions there. So, how did that discussion go? Did you feel you needed to initiate that, or was that…were they very forthcoming?<br>R: It was both, it went both ways. I mean they did explain about the epidural and how it works and what…that it was running all the time and if I wanted extra, I just had to press this little button, and so on and so forth. And then there were other times when they sort of came and said, it's time to take your…whatever pills they were giving me. And then I might say, well I'm not sure, why are you going me those? So, they explained that at that point. **[Patient04_L2]**<br><br>I: And basically, you're just going to be on painkillers, going forward now?<br>R: Not for too long because the injury is only supposed to be for about three weeks.<br>I: Right, okay, so you had injured yourself and that's why you were on the painkillers, do you know whether that…so you said that's not going to be for long. I'm trying to think of what they've talked to you about your plan for your medicines, has anyone come up and said, right this is the plan, what we've got going forward, for your medicines, has that been explained to you at all?<br>R: I've just had a basic outline so far. **[Patient06_L2]**<br><br>I: Has there been any, sort of, well, I mean, actually, one of the things to think about is, were you on any medication before you went into hospital?<br>R: Nothing major, no, nothing major.<br>I: Right, okay. So, and is that, if you were, has any of that continued or has that been stopped?

		R: Well, the only thing that I was on was Simvastatin, and that's a tablet and they said they can't give me a tablet when I'm being sick.<br>I: Yes, quite. So they've stopped that?<br>R: Yeah.<br>I: Right. And did you have a...were you, were you just told that's why and you went, okay, fine, or did you...?<br>R: Yeah, I did ask where my Simvastatin was because it's one of those regular night-time things for me. You know, I've been doing it since my mid-forties and the nurse very, very nicely explained, we can't give you a tablet while you're likely to bring it up, it'd just come straight back. **[Patient08_L2]**<br><br>I: Right, okay. And thinking around that as well then, have you been given any sort of general information about those medicines, you know, like, I don't know, what they're for and side-effects, how you take them, that sort of thing, have you had any of that sort of information?<br>R: Yes, the nursing team passed on the…they've given me a lot of anti-sickness drugs intravenously since the operation and they kept me informed at every stage what has changed with my medication. The medication I took into hospital, I couldn't take, although I didn't question it, I was still under the anaesthetic waiting for it to wear off, I should have asked these kinds of questions, but I just took it as standard that that's how it works. **[Patient10_L2]**<br><br>I: Yes, so I'm with you, so when you left intensive care, no one said, oh, why you're not now on it now, but you said you have had information from the nurses around the anti-sickness medicines?<br>R: Yes, yesterday [inaudible 05:08].<br>I: Yes, right. And do you think…. is it clear, is it understandable, the information?<br>R: Again, a little vague, some of the questions that could…I don't know whether they were reluctant to answer or they couldn't answer.<br>I: Right, okay, so you did ask them questions about that?<br>R: Yes.<br>I: What sort of questions did you ask?<br>R: Why I wasn't getting my medication that was prescribed for me to…and some of their answers were, well, this is what's been prescribed, and this is what's been given to you today. **[Patient10_L2]**<br><br>I: Brilliant, so the first question is really can you just tell me, whilst your mum's been in hospital, what sort of conversations have you had with anybody, you know, any healthcare professional, doctor, nurse, pharmacist, whatever, about the medicines?<br>R: Very little really about the medicines. It was more my conversations were more about how my mum was. I think probably there was…I know when there was a mention, when Mum was very poorly right at the beginning, they were explaining to me why she'd got a lot of water retention and they explained that the medication that they were giving her, I think, to help her heart was affecting the kidney function which was the water retention. Then they talked again about medication that they were giving to try and activate the kidneys again.**[Family11_L3]**<br><br>In terms of the medication, that was all transferred over satisfactorily. And she was taking everything that I expected her to be taking. I'm not a hundred per cent sure they gave her one of her multivitamins one day, but that's neither here nor there. And they seemed to have discontinued the ondansetron, which she'd been taking before she went in. But I think that was probably…well I asked her and it was unnecessary 'cause she hadn't any nausea. But there was something introduced in to her medication which I was not aware of 'til I saw it and they seemed to have put her on a lansoprazole 30 milligram which I thought I recognised but I didn't know what it was. I think they told her what it was or when I asked they told me, so that was a stomach protection. I don't know when she started that, whether it was in the HDU ward and they just carried it forward and I just didn't know **[Family16_L2]**

R: They didn’t actually tell us the names of the medicines, they just said that, when they came to put a drip, a new drip up, they'd say, this is the antibiotics he's on.
I: Right.R: And then, there was another one what was saline, you know, to keep his fluids up.
I: Yeah.
R: And they just explained that, they didn’t tell us any names of drugs he was on. **[Family17_L2]**

I: Indeed, yeah. So, so far, no one has come to you and said, right, what we're going to do is, when he leaves hospital, is we've got, we're going to put him on these medicines, and this medicine, and we're not going to have him on that one he used to be on, nothing like that has happened, at all?
R: No. All they did say was, when he does come home, he'll probably have a couple of weeks of antibiotics to take. They haven't said anything about any alternations, or his medication, or anything, yet.
I: Or his current medication, yeah…
R: No.
I: …the one that he was on before, yeah.
R: No, they’ve never mentioned that.[…] They just said, he'd be on antibiotics for a couple of weeks. **[Family17_L2]**

They’ve not, like I said, they’ve not really told us what they're doing, unless we ask.
I: No, no.
R: We don't know if they’ve changed an antibiotic, or anything.
I: Right, unless you ask them what's going on.
R: They haven't even told us the name of the antibiotic, they just said, he's on an antibiotic, and that’s it.**[Family17_L2]**

R: I think, to be fair, the nurses on the intensive care unit were very explanatory, but I don’t know if that is what they’re normally like or if that’s just because, like I said, I have a bit of an understanding I would ask a lot of questions, so what is this for and why are we giving him this, is this a long term thing, will this affect this, is this good for…so I used to ask a lot of questions. So I’d ask all the blood results, I would ask about his medicine. So if I went one day and there was new medicine added I’d ask what that was for. And before even the nurses had told me in the morning, the first thing I did I would look at the syringe drivers and I’d look at the milligrams and I’d see if any of them had changed because I used to remember them.So if I noticed, I don’t know, his noradrenalin had been put down two points, that would be positive. She would say oh, that’s good, the reason we’ve done it is because he’s tolerated this more, his blood pressure hasn’t gone back up. They would explain what they were doing and why they’d done it. But again, like I say, I don’t know if that’s just because I would ask a lot or that’s what they’d usually do, I’m not too sure. From my experience, I do feel like they did explain it a lot to me. **[Family18_L3]**

R: Yeah, so basically when he first came in last Friday, and they realised how serious his condition was, the conversation I had with the lead consultant in the resus department was the fact that he'd got a rather large or very life threatening large clot on his lung. And they'd given him this particular medicine, which is…it has its risks, like a bleed on the brain. And the name of the medicine was never mentioned, the severity of the clot was never particularly mentioned. I know it was a life threatening one, but how life threatening, don't know. So the only medicine I know that he was given at that time was thrombolysis, which we didn't find out until we'd come from resus to critical care and then to the respiratory ward. Other than that, when they originally went onto the critical care, they were having what they called clot busting medicine, is what they called it in the critical care. And then there were a couple of other medicines which they never really mentioned the names of. I did ask and they just vaguely said oh, this is for so and so, this is for so and so. **[Family20_L2]**

		I: In terms of the way people would talk to you then, how do you feel about how you were…how these things were discussed with you and how the conversations went?<br>R: In regards to in-depth information is what I wanted, pretty poorly up until the point of speaking to the doctor in respiratory, on the respiratory ward. I mean the guys in critical care were fairly vague with what kind of medicine they were giving. They didn't give any names really. **[Family20_L2]**<br><br>R: I mean, it's been a bit tricky, they've not actually been that great at communicating unless we've been pushing them for information, to be honest. So they are very good at coming in and saying to (name), we have, for example, your blood thinner to give you, we're just going to do it in your right arm, as such. Not necessarily, (name), can we do it in your right arm? They will tell us what it is and things like that. However, most of the time, they don't really explain what it's for. I: Right, yeah.<br>R: Yeah, so they might say, I don't, (name), we've got your antibiotics but they don't specifically say these…or haven't specifically said that the antibiotics are for general infection, protection against infection, whether he is currently fighting an infection, you know, it's been a little bit like that. **[Family25_L3]**<br>R: No, no, they've not given us any indication of the plan going forward. We literally get it day by day and that's if we're lucky enough to get the sister on the ward because the sister will explain it. If the sister is there, she will sit down and explain it. If the sister's not there, they'll literally explain to you what they're doing in that moment and they can't give you anything more than that. They just say, the consultants know what they're doing.<br>I: Right. So this is the nursing staff in the general ward?<br>R: The ward, yeah.<br>I: But you can…and in terms of that nursing sister, are you going to her then or how is that?<br>R: Yeah, we approach her. So for example, the other day they were talking about his…well, it was his diet again. They said that (name) could potentially look at starting solids again soon. So I said, right, well, in terms of that, you know, how…what's the plan in terms of timeline and are we doing a little bit, are we doing breakfast and then skipping dinner and tea, is it, you know, breakfast, dinner and tea, what's the plan with it? And they were, like, we're just going to see what happens and, sort of, where (name)'s feeling. Which okay, I get that, he might not want something straightaway but he might want something in the afternoon, I can understand that. But then, you know, I'd say to them, in terms of, like, his nutrition between then, can I ask what the plan is regarding that? Because at this point, (name) had gone three days without any sort of nutrition. And yeah, she just said, oh, you don't have to worry about that, he can cope with just the fluids. And there was no real depth of explanation in that, that was pretty much what she said word for word. **[Family25_L3]**<br><br>R: But from what's been relayed from my mum, it seems very much, a lot of things end up getting debated a lot, and it just, I think, when you have such a mixture of people, with opposing outcomes, we're going to call it. Because, like obviously, everyone is wanting what their speciality is for, to come to the correct outcome […] But sometimes, that obviously impacts what the outcome is of somebody else's speciality. And I think because of that, it just, I wouldn't say I'm particularly personally coordinating it, but I know that my mum sometimes does have issues in knowing exactly what she's meant to be doing […] like, the medication she's meant to be on. Because it's like, because everybody is having opposing opinions, it's very, it does get stressful, thinking that, oh, if I'm going to be taking this, it's going to upset this person, or if I'm going to be doing this a certain way, it's going to upset this person, because they've told me I can't do that. But, yeah, I think, sometimes, it does come down to, like, trying to get everybody together, and actually have a full on discussion with my mum there… **[Family29_L3]**
		They told me about the side effects of tramadol, and only use it to sustain me…you know, don't take it because it says up to three a day. It may say, take three a day. Go with one a day [inaudible 00:07:08] he said, it can have severe side effects. He did tell me quite positively. A lot of what they tell you goes in one ear and goes out the other, kind of thing, it does, you know. But I was told about the tramadol. **[Patient01_L2]**

		I: So, I mean, when it comes to decision making then, you know, they said they decided to change his antibiotic, they've decided to reintroduce some of the regular medicines that had been stopped when he was on critical care, are you involved and included in that decision making?<br>R: No, we weren't included, we were just, like, told what they'd done. Yeah. **[Family03_L2]**<br><br>I: Do you feel these explanations, if you've had explanations about medicines, they've been, as you say, you've been talked to as if you're a child. So, how would you like to be talked to? What would you like to be told when you're given these explanations?<br>R: Just, like, oh, okay,(name) your medicine, now we've changed it. We changed it because we feel that in hospital, right now, you're not eating as much. At home you'd be eating as much, you're not having as much sugar breaking down or these medicines that you're having now, you need to have more. More of these water tablets because it's not working for you or just, do you know, explaining, not just do it. So, they'll just change it without explaining it, yeah. You know when they try to explain it to you, they do it fast. It's really fast. It's confusing. **[Patient07_L2]**<br><br>I: Right, okay. So in terms of then, you know, what's happening now on the ward, has anyone come to talk to you about medicines, you know, had any conversation about that, apart from saying, you know, about being on the paracetamol? Have there been any other conversations?<br>R: No, not really. They keep, you know, they come in the night and say, do you want some paracetamol and anti-sickness? I have had some problems keeping, well, what little bit of food I could have, but that seems to be dissipating now so I'm refusing it. But that's it really, I've got one of these drip machines next to me and I think there's something connected but I don't know what it's doing.<br>I: Oh, right. So you don't know what that's doing, okay.<br>R: No. **[Patient08_L2]**<br><br>I: Yeah, right, okay. When he moved from critical care to the ward, was there any sort of further, you know, information given to you at that point?<br>R: No. They waited while we were there, before they moved him, so we could find out what ward he was on.<br>I: Right, okay.<br>R: And we went with him to the ward.<br>I: Right, okay. But was there any…R: Nobody, there was no proper update, they just said they were pleased with him, and he could move onto a ward, that's all they said.<br>I: Right, so there was no conversation about anything that was going to be happening with the medicines, or anything like that, at that particular point?<br>R: No. **[Family17_L2]**<br><br>R: They've not, like I said, they've not really told us what they're doing, unless we ask.<br>I: No, no.<br>R: We don't know if they've changed an antibiotic, or anything.<br>I: Right, unless you ask them what's going on.<br>R: They haven't even told us the name of the antibiotic, they just said, he's on an antibiotic, and that's it. **[Family17_L2]**<br><br>I: …one to one, as you say, to one to six or seven or eight or whatever. So in terms of then the information that's coming to you do you have to search for more? Do you have to ask more questions, or have you had to ask more questions since he's been on the ward?

		R: Yeah. I do ask, because when I visit him in the morning the first thing I do as soon as I see a nurse I'll ask about his CRP levels, his pain levels and your blood results and what's going on at the minute. That's the first thing that I ask usually. And, to be fair, they will tell me what the levels are. But again in intensive care when I used to go and ask a level they'd say oh, but it's been trending from this and this is a good incline, they'd give me a bit more information, whereas they'll just tell me a number, basically. So I think it's harder to get the information because you do have to look for it, you have to ask a lot. Like today, for instance, I've been asking…because basically he's been in intensive care for all this time and then he's been on the ward, but now he's only having IV antibiotics, he's looking after himself, he's got a lot steadier on his feet, he's eating and drinking fine, kidney function's a lot better. So he is a lot better. So it's just trying to find out what the plan is now. Because obviously he's been in hospital nearly a month now, he's desperate to go home. **[Family18_L3]**<br><br>R: Yeah, so basically when he first came in last Friday, and they realised how serious his condition was, the conversation I had with the lead consultant in the resus department was the fact that he'd got a rather large or very life threatening large clot on his lung. And they'd given him this particular medicine, which is…it has its risks, like a bleed on the brain. And the name of the medicine was never mentioned, the severity of the clot was never particularly mentioned. I know it was a life threatening one, but how life threatening, don't know. So the only medicine I know that he was given at that time was thrombolysis, which we didn't find out until we'd come from resus to critical care and then to the respiratory ward. Other than that, when they originally went onto the critical care, they were having what they called clot busting medicine, is what they called it in the critical care. And then there were a couple of other medicines which they never really mentioned the names of. I did ask and they just vaguely said oh, this is for so and so, this is for so and so. **[Family20_L2]**<br><br>I: But you do know what they are?<br>R: No, I don't know, I know there's a little heart-shaped one, there's couple of capsules, and that's about it. I don't know what they are. I'm pretty good […] with medicines as well, but I don't know. Nobody's actually come round and sat down and said, you're on this tablet, this tablet, this tablet, and this is why, nobody has done that.<br>I: Right, right. But you know that these are for what they're for, you know that they're for heart and painkiller and your stomach, is that what you said?<br>R: Yeah, that's what they've told me. That one's for your heart, that one's for your pain, that one's for your tummy. **[Patient22_L3]**
		They just explained that obviously the stronger pain relief in my back would be stopping so I'd probably need some other and explained that if I needed the Oramorph was if I asked for it, I wouldn't just get given it, it was if I was in pain I'd ask for it at any point and they explained how over many hours I could have it between that as well, so it was explained well to me to be fair. **[Patient02_L2]**<br><br>It's normally the nurse who dispenses, and of course that's under doctor's orders. So, I know this can do…you know, oh this does this, and it does this. And there's no…how can I put it? There's no situation which they didn't explain thoroughly. **[Patient05_L3]**<br><br>I: Right, okay, and in terms of that conversation then, around that, or at that time, who was it who talked to you?<br>R: I would say a lot of that was explained by a sister that was doing…when Mum was really poorly and she was, kind of like, working with her constantly there was a sister that worked with her and she explained that to us in quite some detail.<br>I: So it was basically around nursing staff then who would do that.<br>R: Yes, I would say most of my conversations were with the nursing staff because they were the ones that were doing the main care for her. I mean, had I requested a conversation with a doctor that would have been…you know, that was possible but I found that I was happy with the information that I was getting from the nursing staff, particularly this…this particular sister was really good. **[Family11_L3]**<br><br>I: Yes, okay. And did you have the opportunity to ask any questions or anything like that?

		R: Yes, definitely. I remember her saying that.<br>I: Right, okay. And how were those questions answered, you know, was it satisfactory, was it good?<br>R: I felt quite happy, yes, I think we discussed the options on various opioids and what have you, and I decided definitely to go with the lower strength options, given the drawbacks of some of the stronger ones. I: Right, okay. So, you would talk…so side effects and stuff like that were talked through to with you?R: Yes, I kind of have got a bit of a…well, I was married to a nurse for 27 years, so I've got a bit of sort of medical knowledge, and I do know the facts anyway of…**[Patient12_L2]**<br><br>And they were brilliant, they explained everything, how everything had gone. Yeah, I can't honestly remember all the specific details as to what… With regards to what medicines she was on and stuff like that. I know she was on a PICC line and she'd been on that before her surgery, before Critical Care, she was still on that for a while after Critical Care because she couldn't eat, still. That got explained to us again. I mean, we already knew but they took the time to explain it all to us again. Yeah, they were really good. **[Family15_L3]**<br><br>Oh yes, here it is. Yes. They sent me a complete list out of everything that she was supplied with, including sharps, containers, anti-embolic stockings, all the medications she went in with, of course some of them…we took the medications in as requested so some of them they brought out were ours, prior to the hospital visit and some were theirs. Was there anything extra? No, they gave her some Laxido, which they explained was for constipation should she get it, which would be quite a novelty because she went in taking 24 loperamide a day plus six codeine a day and she still got diarrhoea three/four times a day. **[Family16_L2]**<br><br>R: She just phoned up and explained what was happening, this woman […] And then she said, your doctor will be in touch, and they'll decide whether you need the antibiotics […] And it all happened within 24 hours<br>I: Right, right. Right, but you got, as you say, you got that email, yeah.<br>R: She did send us some information via email, to download and read<br>.I: Right, yeah, and that was helpful, was it?<br>R: Yes, it was. **[Family17_L2]**<br><br>I: And what were those conversations like? Were they useful or helpful or…?<br>R: I think they were helpful. I think obviously in my own experience working in that environment, I think nurses in general are a bit better at explaining things in layman's terms rather than doctors and consultants who use big words and stuff. But yeah, obviously at that time when things are quite up in the air, it's a lot to take in anyway, isn't it, for anyone. But I think the nurses did really do a good job in explaining what was going on in regards to medicine. **[Family18_L3]**<br><br>I asked a couple of times what he was being given, painkillers, and I think that Tramadol was mentioned at one point and, oh, he was given something to stop his lungs from, I don't know all the medical names for all these things, well he was given something to stop his lungs from filling up with fluid. He was given antibiotics, yes, it was all explained to me as they were giving it, you know. When we came, [inaudible 00:05:00] was, sort of, in the evening, the consultant saw us twice and the nurses gave us all the information we ever asked for, you know. So we were all aware of what he was, that he was getting his full medication. **[Family23_L2]**

		R: ...I was going to say, on a couple of occasions they mentioned something that I didn't understand, you know, they mentioned a name of the medication. And I said, well what does that do? And they explained it, that was, well we're giving him that because of this. And even down to his oxygen, he had to go in, like an oxygen thing, like a bubble really, which he hated. And they explained to me, it was like a CPAP thing, and they explained to me why he had to be in that, because the oxygen levels, they were pumping the oxygen levels into his lungs. And then, gradually, as his lungs improved they took him off that and put him on different types of oxygen, and all that was explained as well.<br>Because, you know, obviously, when you see my dad in, like, a plastic bubble, you want to know why he's in there. And it was all explained why, why he had to be there. And, you know, every question we asked they answered, if I wasn't sure if he was being given a medication for something that the consultant had mentioned, and I said, well what was that for? The critical care nurse explained it, what it was for. **[Family23_L2]**<br><br>We were even told when he was first admitted to A&E that his injuries were, well, they were similar to what you get in a car accident. And at the end of the day he was 86 and it was explained to us, I mean, they were quite honest about the fact that he was seriously ill. And, [inaudible 00:09:24] try and save his life. So I've got absolutely no complaints at all, every question my brother asked or my sister asked, it was all explained by the critical care nurse why they were doing it. We asked about his oxygen levels, were they improving and they explained all that. We could see on screens, numbers, and they even explained, because we weren't sure what were his oxygen levels and what was something else because they can be a bit complicated, those. **[Family23_L2]**<br><br>R: But if ever I wanted to ask a question, if ever I was concerned about something or I wasn't sure what was going on, the staff on critical care were amazing and would always take the time to sit down and go through it all with me again. And even though I couldn't communicate either because I had a trachea in that prevented me from speaking, even to the point where the staff they knew that I was getting frustrated because people couldn't lip read particularly well, so they got me a board so I could write on it and communicate that way. So, I feel like…yeah, it was a bit strange because obviously choices were out of my hand to begin with, but when I was communicative and once I had questions, people had all the time in the world to explain to me what was happening. So, I feel like I've had as much communication as I possibly could have.<br>I: And you said they facilitated even when you couldn't speak with the board as well, which is fascinating, isn't it? Very helpful obviously.<br>R: It was such a small gesture that was made by…I think…she wasn't a nurse she as one of the support workers, such a simple gesture, I just said to her before I left critical care unit, I know it took you minutes to go and find this thing but, I said, you made a world of a difference, because I didn't realise…obviously my role in a job and I'm also a volunteer, there's a lot of communication involved, and I didn't realise how restricted it is when you can't just talk. And that simple gesture of her taking two minutes to go and find that board to give to me, it made…well, it made my month basically, it gave me my month back and I just said to her, that was amazing and thank you so much, you have no idea the difference you've made. I definitely let everybody know on the ward that they did fantastic. **[Patient24_L3]**<br><br>I: Yes, that's something which a lot of people have said to me as well. Do you think patients…how do you think patients can be involved in decisions around their medicines? What's the best way for them to be involved in that?<br>R: I think it's just telling people what it is, what it does, what it will enable them to do, what side effects there might be, what alternatives there might be. So, it's just having all the information to hand I think and maybe also, especially for the likes of me that wasn't amazingly compos mentis to begin with when I was coming around from the strong medication, having somebody there with them, whether it be somebody from the medical staff, a nurse that could then maybe explain it in slightly different terms or better still a member of family that will retain that information and be able to remind them of the conversations you had. Yeah, having somebody other than just the patient, because I think sometimes we're not always there, sometimes our brains not on […] Sometimes we're sleep deprived. On the wards I think we're all just so very sleep deprived. So, it might take a bit more time to get information into our heads than on a regular day anyway. **[Patient24_L3]**<br><br>I: What were things like on critical care, just to, you know…?

		R: Completely different. And I mean completely different. So he had a nurse called (name) who I met on the first day of (name) being in critical care, straight after his surgery. And she was fantastic. Everything she did, she explained with detail about what they were doing, why they were doing that, how they were going to do that, what to expect when that happened, don't be alarmed. And she was, at any point saying, you know, if you have any questions, just ask me. If she did something on one of (name)'s machines, she thoroughly explained it and then again, you know, explained why. It was a completely different atmosphere. Everything, you felt so relaxed all the time because you fully understood everything that was happening. **[Family25_L3]**
		I: …because you're looking for that in terms of how it's all being explained going forward. So, I mean, when it comes to decision making then, you know, they said they decided to change his antibiotic, they've decided to reintroduce some of the regular medicines that had been stopped when he was on critical care, are you involved and included in that decision making?<br>R: No, we weren't included, we were just, like, told what they'd done. Yeah.<br>**[Family03_L2]**<br><br>R: Well, they've not mentioned it. I found it in the bottom of the bag.<br>I: Right, okay. But no-ones told you any reason why you're on this.<br>R: Nobody told me they were going to put it in, and no, I've no idea what it's for, but it has got my name on the packet.<br>I: Right. So, it's for you, it's the right one. What do you feel about the fact that no-ones talked to you about that?<br>R: I would have preferred if they had.<br>I: Right. And do you think you might try and find out from someone or…?<br>R: I'll try and find out what it's for before I use it.<br>I: Yeah, okay. There should be the information leaflet in there, shouldn't there, which might help, I suppose.<br>R: Yeah.<br>I: So, you would've preferred…?<br>R: I'd forgotten all about it.<br>I: Yeah. You would have preferred to have known why…?<br>R: Yeah. Well, it's slightly disturbing just to find this thing at the bottom of the bag and wonder what it's for, but I did check it and it was for me. **[Patient14_L3]**<br><br>In terms of the medication, that was all transferred over satisfactorily. And she was taking everything that I expected her to be taking. I'm not a hundred per cent sure they gave her one of her multivitamins one day, but that's neither here nor there. And they seemed to have discontinued the ondansetron, which she'd been taking before she went in. But I think that was probably…well I asked her and it was unnecessary 'cause she hadn't any nausea. But there was something introduced in to her medication which I was not aware of 'til I saw it and they seemed to have put her on a lansoprazole 30 milligram which I thought I recognised but I didn't know what it was. I think they told her what it was or when I asked they told me, so that was a stomach protection. I don't know when she started that, whether it was in the HDU ward and they just carried it forward and I just didn't know [voices overlap 03:49]. **[Family16_L2]**<br><br>R: But…yes, if you're trying to pick out a…an area of slight lack of communication, I didn't know why it had been started. I still haven't been told why it was started. I've been given a pack of 28 which I presume we're going to be taking for that time and I don't know when it's going to stop. Well it will obviously stop whenever I run out of that pack and if the doctor says…GP when say…well I will request should she need it. Another member of the public might just ask for it again. The only difference being I will ask, does she need this anymore, can someone check how long she's supposed to be on it. But that's the only difference between me and, say, you know, a non-pharmacist. **[Family16_L2]**

R: They've not, like I said, they've not really told us what they're doing, unless we ask.
I: No, no.
R: We don't know if they've changed an antibiotic, or anything.
I: Right, unless you ask them what's going on.
R: They haven't even told us the name of the antibiotic, they just said, he's on an antibiotic, and that's it. **[Family17_L2]**

R: So yeah. So probably about four times a day I get a pot full of tablets.
I: Right. Okay.
R: So there's paracetamol, gabapentin, omeprazole-type…one of them, lansoprazole. Let me think, a couple of different types of blood pressure tablets, and no doubt there was a statin in there of some sort.
I: Yeah. Right. Okay. And again, in terms of you say four times a day you get a pot load of medicines; when they bring those along to you does someone talk you through that, or is it just here's your medicines, (name) , and, you know, crack on, as it were? Or is it a conversation about, all right, we're changing that, or doing that, whatever?
R: The majority of time it's literally, here's your tablets, and there you go. **[Family19_L3]**

Yeah, so basically when he first came in last Friday, and they realised how serious his condition was, the conversation I had with the lead consultant in the resus department was the fact that he'd got a rather large or very life threatening large clot on his lung. And they'd given him this particular medicine, which is…it has its risks, like a bleed on the brain. And the name of the medicine was never mentioned, the severity of the clot was never particularly mentioned. I know it was a life threatening one, but how life threatening, don't know. So the only medicine I know that he was given at that time was thrombolysis, which we didn't find out until we'd come from resus to critical care and then to the respiratory ward. **[Family20_L2]**

R: So, I mean, when she was on Critical Care, the initial stages we weren't really involved in what they were giving her. It was only really when she'd come round a little bit, that we found out that, I knew she was having paracetamol, antibiotics for a chest infection, and some heart medication.
I: Right, okay.
R: And so they were given to her without really, now whether they discussed it with her husband, I don't know, but initially I don't think we were aware of what she was being given, we just wanted to know that she was alive basically.
I: Right, yeah, absolutely.
R: And then later down the line in Critical Care, she was given oramorph for her broken breastbone, sternum, and well, we didn't, I didn't get asked about that, and whether her husband did I don't know. **[Family21_L3]**

I: But you do know what they are?
R: No, I don't know, I know there's a little heart-shaped one, there's couple of capsules, and that's about it. I don't know what they are. I'm pretty good…
I: But you are…
R: …with medicines as well, but I don't know. Nobody's actually come round and sat down and said, you're on this tablet, this tablet, this tablet, and this is why, nobody has done that. **[Patient22_L3]**

I: So it's not…they were saying, oh, this is your antibiotics but they weren't providing you with further information like exactly why they were doing that and for what reasons they were?

		R: Yeah, that was pretty much for a lot of it. A lot of it I had to say, so the blood thinners, for example, (name) is currently on blood thinners. Now I know in the healthcare profession that blood thinners, if someone's laid on the bed, it's quite common to be given, especially in hospital after big surgery, to avoid blood clots. However, his wife doesn't know that. So as soon as they left, the first thing she asked me was, why is (name) having blood thinners? She has no idea. So in her head, she thinks now (name)'s, you know, potentially a high risk of clots but don't know what or why, is that because of the surgery, is that because of all this? But she had no…so I had to sit down and explain it. And then it's just watching that fear go away that…you know, so I know why they're doing that but they didn't explain why they were doing that, I had to be the one to tell his wife that. **[Family25_L3]**
		R: There was a leaflet, there was indeed a leaflet from the epidural team, I seem to remember, and I think I've still got it. She gave it to me…I remember somebody saying, here's some light reading. […] But I cannot tell you what was in it.<br>I: Right, okay. So, you did you read it?<br>R: I did, I think I did at least once.<br>I: Oh right, okay. And was that clear and understandable or was it…?<br>R: I seem to remember it was. **[Patient12_L2]**<br><br>Oh yes, here it is. Yes. They sent me a complete list out of everything that she was supplied with, including sharps, containers, anti-embolic stockings, all the medications she went in with, of course some of them…we took the medications in as requested so some of them they brought out were ours, prior to the hospital visit and some were theirs. Was there anything extra? No, they gave her some Laxido, which they explained was for constipation should she get it, which would be quite a novelty because she went in taking 24 loperamide a day plus six codeine a day and she still got diarrhoea three/four times a day. **[Family16_L2]**<br><br>R: Well, when he first got diagnosed, it was Strep A pneumonia.<br>I: Yeah.<br>R: And we did get somebody phone us up from the hospital, because they wanted my son, what still lives with us, and me, to go on antibiotics as well.<br>I: Oh, right, okay.<br>R: So, and they did explain, and they did give us some paperwork on it… I: Right, okay. R: …to read up on it, to understand what it was, so that…yeah, that was helpful.<br>I: Yes.<br>R: But apart from that, I don't think any other information would have made any difference.<br>I: No, okay. But that was really helpful to have that phone call?<br>R: Yeah, because we didn't understand it.<br>I: Yeah, precisely, yeah. Yeah, well that's good.<br>R: It's like, you go on the internet and look it up and it could be horrifying…I: I know, yeah.<br>R: …can't it, you know, reading it?<br>I: Yeah.<br>R: Whereas, they did send us some via email to read up on. **[Family17_L2]**<br><br>Have you been given any general information in terms of written information about any of these medicines? Is there anything sort of like a general information sheet, or a summary sheet, or anything like that?<br>R: I've had a… They did a diary for me which is being documented every day, you know, what nurses were looking after me, and what happened to me that day, you know, and did I have any ups and downs, that sort of thing.<br>I: Right.

		R: And that's…[inaudible 0:21:22], kind of thing, gives you quite a lot of information and insights, what was going off on that particular day. **[Family19_L3]**
